# Nanomaterial-based electrochemical sensing of neurological drugs and neurotransmitters

**DOI:** 10.1007/s00604-014-1308-4

**Published:** 2014-07-08

**Authors:** Bankim J. Sanghavi, Otto S. Wolfbeis, Thomas Hirsch, Nathan S. Swami

**Affiliations:** 1 Department of Electrical and Computer Engineering, University of Virginia, Charlottesville, VA 22904 USA; 2 Institute of Analytical Chemistry, Chemo- and Biosensors, University of Regensburg, Regensburg, 93040 Germany

**Keywords:** Nanomaterials, Electrochemical sensors, Neurological drugs, Neurotransmitters, Voltammetry

## Abstract

Nanomaterial-modified detection systems represent a chief driver towards the adoption of electrochemical methods, since nanomaterials enable functional tunability, ability to self-assemble, and novel electrical, optical and catalytic properties that emerge at this scale. This results in tremendous gains in terms of sensitivity, selectivity and versatility. We review the electrochemical methods and mechanisms that may be applied to the detection of neurological drugs. We focus on understanding how specific nano-sized modifiers may be applied to influence the electron transfer event to result in gains in sensitivity, selectivity and versatility of the detection system. This critical review is structured on the basis of the Anatomical Therapeutic Chemical (ATC) Classification System, specifically ATC Code N (neurotransmitters). Specific sections are dedicated to the widely used electrodes based on the carbon materials, supporting electrolytes, and on electrochemical detection paradigms for neurological drugs and neurotransmitters within the groups referred to as ATC codes N01 to N07. We finally discuss emerging trends and future challenges such as the development of strategies for simultaneous detection of multiple targets with high spatial and temporal resolutions, the integration of microfluidic strategies for selective and localized analyte pre-concentration, the real-time monitoring of neurotransmitter secretions from active cell cultures under electro- and chemotactic cues, aptamer-based biosensors, and the miniaturization of the sensing system for detection in small sample volumes and for enabling cost savings due to manufacturing scale-up. The Electronic Supporting Material (ESM) includes review articles dealing with the review topic in last 40 years, as well as key properties of the analytes, viz., pK_a_ values, half-life of drugs and their electrochemical mechanisms. The ESM also defines analytical figures of merit of the drugs and neurotransmitters. The article contains 198 references in the main manuscript and 207 references in the Electronic Supporting Material.

**Electronic supplementary material:**

The online version of this article (doi:10.1007/s00604-014-1308-4) contains supplementary material, which is available to authorized users.

**Figure:**
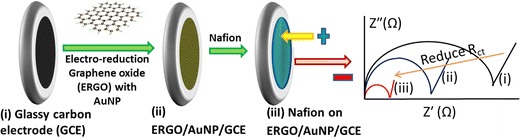
ᅟ

## Introduction

Quantitative analysis of pharmaceuticals is essential during various phases of drug development and during the fabrication stage, to ensure appropriate formulation, stability and quality. Other major fields include toxicology testing in pharmacology and in the clinical trial phase for monitoring bio-availability, pharmacokinetics and possible drug abuse. The analysis of neurological drugs is especially challenging due to the need for simultaneous detection of multiple analytes within highly complex sample matrices and at high levels of spatial and temporal resolution [1].

While the analysis of pharmaceuticals has historically been carried out by spectrophotometry, fluorometry and liquid or gas chromatographic methods [2], recent investigations have focused on electrochemical methods [3–5], especially for neurological drug analytes [6, 7]. Electrochemical methods enable several unique advantages [8–10]. These include (1) ease of analysis due to a lack of the need for excessive sample preparation; (2) selectivity due to electrical signals at characteristic formal potentials, which can enable detection of multiple analytes without separation steps; and (3) the ability to analyze within biological matrices, including sweat, urine, serum, and cell culture media. Electrochemical methods also have reasonably fast sampling times, can offer valuable insights on the metabolic fate of the drug at particular dosage levels inside the body, and allow for investigations of the interaction of drugs with living cells. Limitations posed to electrochemical analysis include the need for significant electroactivity of the analyte of interest, complications from interfering species that may be present at far higher concentration levels than the analyte of interest, signal and background drifts due to electrode fouling and charging, and, finally, the lack of a strategy for simultaneous detection of multiple targets, which is especially essential for monitoring neurological processes.

**Fig. 1:**
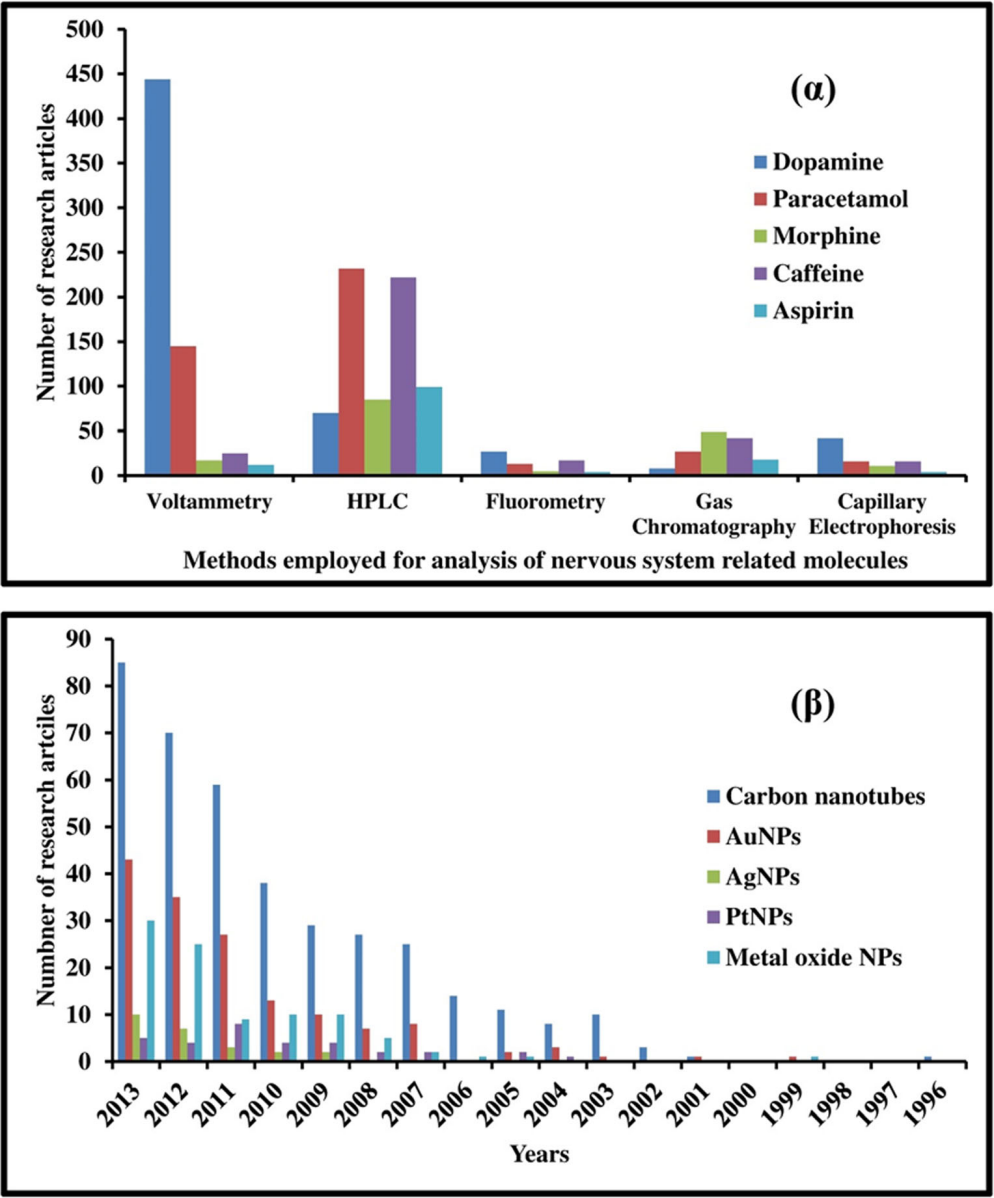
(α) Number of publications for analysis of key neurological targets, classified based on type of analytical technique (from 2010 to 2013). Results obtained from SciFinder ^tm^. (β) Number of publications for dopamine analysis using different nanomaterials (from 1996 to 2013). Results obtained from Scifinder^tm^

Notwithstanding these challenges, a survey of the literature (see Fig. 1α) indicates the dominance of electrochemical over other methods for the analysis of dopamine [11], and the increasing importance of electrochemical analysis of paracetamol, morphine, caffeine and aspirin [4, 12–14]. Frequently applied electrodes include those made from carbon pastes, glassy carbon, glassy carbon pastes, diamond, carbon ceramic, edge plane pyrolytic, basal plane pyrolytic carbon pyrolytic graphite and carbon screen-printed electrodes. It is noted here that fiber optic sensors for drugs and neurotransmitters have recently also gained in popularity [15].

The synthesis of materials in the 1 – 100 nm size range is becoming increasingly important due to the gains offered by their functional tunability, versatility, ability of self-assembly [16, 17], and the emergence of novel properties of materials at this length scale [18–20]. Specifically for the case of electrochemical detection systems, nanostructuring of the working electrode through the application of nano-sized modifiers offers attractive new features [21, 22]: (1) Nanostructured electrodes, especially those using carbon nanotube and graphene-based materials, exhibit faster electron transfer kinetics due to their extremely high conductivity along particular directions [23]. (2) The high surface area of nanostructured electrode modifiers can enhance the adsorption kinetics of analyte species, which is especially useful in enhancing peak currents within DPV and SWV techniques that utilize adsorptive stripping to cause analyte pre-concentration. (3) Nanostructured materials can function as highly selective and tunable catalysts, due to their unique electronic or plasmonic structure. This is especially useful within detection systems utilizing electrocatalysis. (4) The surface chemistry of nanostructured systems can be tuned towards directing the assembly for particular capture probe or analyte species [24]. Such selective immobilization methods are enabled through nanomaterial modifiers and offer microarray-based pathways towards parallelized detection.

Focusing solely on the detection of dopamine, which is one of the most popular neurological drug analytes detected by electrochemical means, we show in Fig. 1 (β) the exponentially growing interest in using nanomaterial-based modifiers, led by carbon nanotubes and followed by metal and metal oxide nanomaterials. In many cases, surfactants, conducting polymers and room temperature ionic liquids are required to better disperse the nanomaterial systems. Hence, we have included them within the current review to illustrate how they may be applied to improve detection. Recent reviews cover general aspects of nanomaterial-modified electrodes [25–27] including biogenic amines [11]. Aspects of in-vivo biosensors using nanomaterials have been reviewed by Zhang et al. [28]. A list of review articles up from the year 1980 is given in Table S1 of the Electronic Supporting Material (ESM). This list also demonstrates how impressively the field has progressed in recent years.

This critical review, in contrast, is focused on the broad class of neurological drugs and neurotransmitters. We focus on an understanding how specific nanomaterial modifiers may be applied to influence the electron transfer events involved in the detection of key neurological drugs and the gains in sensitivity, selectivity and versatility that are obtained due to such modifications. We also decided to organize the review according to the type of analyte in order to enable a comparison of how nanomaterial modifiers may be coupled to the different voltammetric approaches. This review article initially offers a brief description of carbon based electrodes. This is followed by a section dealing with commonly used supporting electrolytes in voltammetry. The next section offers a brief description of the carbon based electrodes covered in this review. This section is followed by a description of the properties and electrochemical reactions of the code N drugs. Thereafter, we evaluate the detection of drugs belonging to Anatomical Therapeutic Classification (ATC) code N (groups N01 to N07) and neurotransmitters. The next section classifies the work for each code N drug in terms of the electrode modifiers, voltammetric techniques, biological matrices, and detection systems. Finally, some future challenges are discussed. Given the challenges in optimizing and implementing nanomaterials based sensors, we envision that this critical review can guide potential users towards exploiting the advantages offered by nanomaterials for electrochemical analysis.

Anatomical Therapeutic Chemical (ATC) Classification System has been used here for the classification of drugs (www.whocc.no/atc_ddd_index/). This classification is governed by the WHO Collaborating Center for Drug Statistics Methodology (WHOCC) and was first published in 1976. This system divides drugs into different subgroups according to the organ or system, on which they act and/or their therapeutic and chemical characteristics. Each bottom-level ATC code stands for a pharmaceutically used substance, or a combination of substances, in a single indication (or use). This means that one drug can have more than one code. Acetylsalicylic acid (aspirin), for example, has the code A01AD05, in being a drug for local oral treatment, the code B01AC06, in being a platelet inhibitor, and also the code N02BA01, because it acts as an analgesic and antipyretic. On the other hand, several different brands share the same code if they have the same active substance and indications.

## Types of carbon-based electrodes

This section gives a brief overview on the carbon based electrodes referred to in later sections and covers their manufacture, features, advantages, and disadvantages.

### Carbon paste electrodes

In the late 1950s, a new “dropping carbon electrode” (DCE) was introduced by Professor Ralph N. Adams (1924-2002) as an alternative to the dropping mercury electrode (DME). It was specifically designed for anodic oxidations of organic compounds, for cases where the DME failed or could not be used. Although this concept finally failed, a thick mixture of carbon paste (CP) was obtained [29]. This led to the birth of carbon paste electrodes (CPE). In this article, the CPE was prepared by employing 1 g of graphite and 7 mL of bromoform as the binder (which is a rather high quantity of binder to be used for making a carbon paste). Experience tells that if such a high amount of binder is used to make a paste, oil starts leaching into the electrochemical cell.

**Fig. 2:**
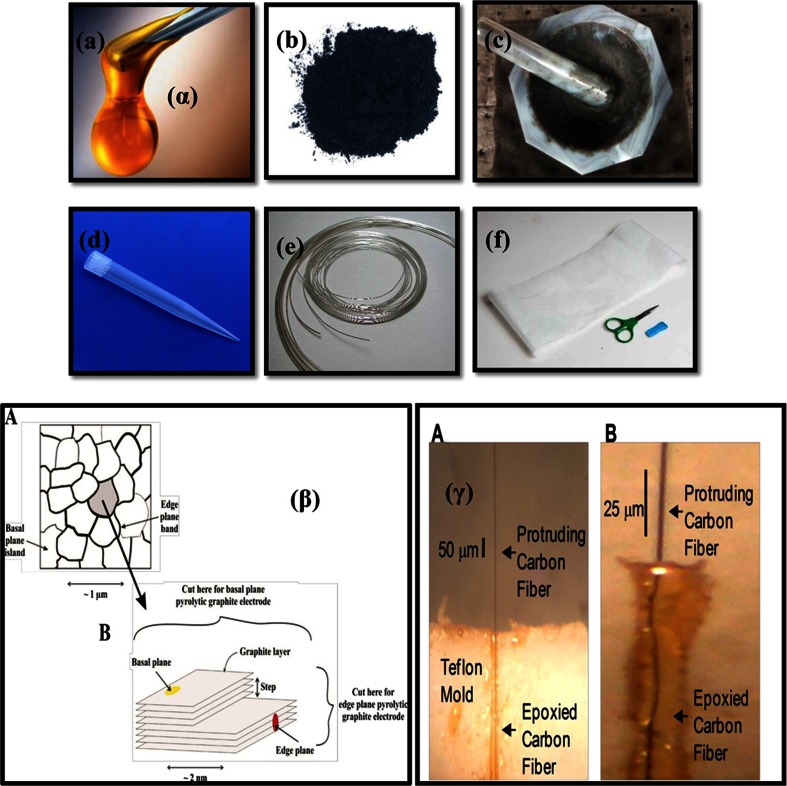
(α) Fabrication procedure of a carbon paste electrode: a) Binder, b) Graphite powder, c) Homogenized carbon paste, d) Teflon micropipette tip, e) platinum wire, f) weighing paper. (β) Surface defects present in basal plane pyrolytic graphite electrode and edge plane pyrolytic graphite electrode [18, 30]. (γ) Picture of epoxy coated electrodes. (A) Epoxide CFME in the Teflon mould, (B) Epoxide CFME after removal from the Teflon mould [31]

The following procedure is typically used for the preparation of a carbon paste electrode (CPE). First, a carbon paste is prepared by mixing graphite and binder in 60:40 or 70:30 (w/w) ratios. The binder [Fig. 2 α, (a)] can be paraffin oil or mineral oil (nujol), although ionic liquids have been employed in recent studies. This is added to the graphite powder [Fig. 2α, (b)], the paste is mixed thoroughly in a mortar and pestle and allowed to undergo self-homogenization for 24 h [Fig.2α, (c)], and the paste is then filled into a syringe or a micro-pipette [Fig. 2α, (d)]. A metallic wire [Fig. 2α, (e)] is then dissected through the paste to provide an electrical contact. Smooth and fresh electrode surfaces are obtained by squeezing out ~0.5 mm of paste from the syringe, scraping off the excess and polishing it against weighing paper [Fig. 2α, (f)] until the surface has a shiny appearance. A pictorial description of this whole process is provided in Fig. 2α.

CPEs have various advantages and limitations. They can be used to study electron transfer processes in the cathodic as well as the anodic range. However, beyond +1.4 V, a large background current is obtained and it becomes difficult to differentiate the analyte peak from the background signal. Moreover, they are easy to fabricate, and the cleaning of electrode surface is easy. The surface of the paste electrodes must be renewed after every run, else fouling of the electrode surface can cause considerable irreproducibility in the peak currents. The biggest disadvantage of CPEs, which limits their applicability in practical analysis, is that electrochemical analysis with carbon paste-based electrodes requires an adequate experience of the user. In contrast to commercially available solid electrodes (for which basic electrochemical characteristics are comparable across all products from each manufacturer), each carbon paste unit can have individual physical, chemical and electrochemical properties that may differ from one preparation to the other. For this reason, each probe must be calibrated individually. If not calibrated well, the results can be very erroneous. While this may not be a major issue in a research environment, it can represent considerable burden for a large scale industrial production. Accurate analytical treatment must be given to the data (viz., calculating % RSD, coefficient of variation, standard deviation). For enhancing the sensitivity of the paste electrode, researchers instead use glassy carbon paste electrode (GCPE). It performs better than CPEs, due to the uniform structure of graphite particles in GCPE versus CPE.

### Glassy carbon and pyrolytic graphite electrodes

Inspired by the work of Adams [29], glassy carbon was employed as an electrode material [32]. The glassy carbon electrode (GCE) has been employed widely ever since to determine various organic molecules and metal ions. To obtain well-defined surfaces, the electrodes have to be abraded with emery paper of increasing fineness, followed by polishing with, for instance, suspensions of alumina or chromium (III) oxide (particle size 0.3 μm) [33]. The good resistance of glassy carbon against chemical attack makes it possible to apply the electrode in highly acidic and alkaline media. It has many properties in common with pyrolytic graphite, except that it is isotropic. An electrode of pyrolytic graphite must be properly oriented, and the edges of the planes of the graphite must be sealed. These limitations do not exist for an electrode made of glassy carbon. GCE have a much higher sensitivity compared to the CPE. On the other hand, GCEs have to be polished thoroughly so to avoid fouling of the surface. By experience, the polishing involves taking around 50 mg of alumina powder on a weighing paper, followed by placing one drop of water in the powder so as to make a paste. The GCE is then held perpendicularly (holding it perpendicularly is extremely important otherwise, only the Teflon part will be renewed and not the electrode surface) onto the alumina paste and then polished. If the molecule has adsorbed very strongly onto the electrode surface, this cleaning process can be very time consuming and a tedious process. However, if polished well, drastic current changes are not seen in case of GCEs as compared to CPEs.

### Edge plane and basal plane pyrolytic graphite electrodes

Compton’s group [18, 34] has described edge plane and basal plane pyrolytic graphite electrodes made from highly ordered pyrolytic graphite. The basal plane surface of such an electrode consists of layers of graphite oriented in parallel to the surface and with an interlayer spacing of 3.35 Å (Fig. 2β). Surface defects occur in the form of steps exposing the edges of the graphite layers. Due to the nature of the chemical bonding in graphite, the two planes (edge and basal) exhibit completely different electrochemical properties. The edge plane exhibits considerably faster electrode kinetics in comparison to the basal plane. This implies that in many instances an electrode consisting entirely of edge plane viz. an edge plane pyrolytic graphite electrode will show a nearly reversible voltammogram while an electrode consisting mainly of basal plane graphite will show irreversible behavior. Even though these electrodes have a lower background current compared to CPEs, those available from vendors are extremely large in diameter. Thus, miniaturization of these electrodes is a necessity if these electrodes have to be employed for multi-target analysis or within in vivo experiments.

### Boron doped diamond electrodes

Boron is a widely used dopant to produce conducting diamond electrodes. This is because of boron has a low charge carrier activation energy (0.37 eV) [35]. Boron doping leads to a p-type semiconductor. At low doping levels, the diamond acts as an extrinsic semiconductor. At high doping levels, the material acts as a semi-metal. To introduce boron into the diamond material during film growth, a boron containing substance has to be added to the deposition gas mixture. Substances such as diborane or trimethyl borane can be used. The majority of studies with doped diamond electrodes use boron-doped diamond.

The use of boron-doped diamond (BDD) as an electrode substrate is now well established, mainly due to favorable properties such as a wide potential window in aqueous solutions, low background currents, long term stability, and low cross-sensitivity to dissolved oxygen [36]. These properties make BDD electrodes particularly suitable for electrochemical studies of analytes with a high oxidation potential [37, 38]. In BDDs, the replacement of approximately one carbon atom in a thousand by a boron atom yields a material with metallic conductivity. The properties of BDDs are known to be affected by the quantity and kind of doping agent, morphological factors and defects in the film, presence of impurities (sp^2^ carbon), crystallographic orientation, surface termination (hydrogen or oxygen), and electrochemical pre-treatments of its surface [39, 40].

Doped-diamond electrodes have been introduced into electrochemistry by Pleskov et al. [41]. They are mainly applied to aqueous electrolytes. The most striking feature here is their very high overpotential for both oxygen and hydrogen evolution [42, 43]. This enables a wide potential window (approx. 3.5 V), which can be used for monitoring other electrochemical reactions in aqueous electrolytes. Diamond electrodes have indeed the largest potential window measured thus far in aqueous electrolytes. This makes them totally different from common electrode materials such as gold, platinum or mixed metal oxide type electrodes. A recent review on BDDEs [44] describes its utility as an electrode material. The only disadvantage of BDDE-as compared to other carbon electrodes – is the need for being doped with boron, which is a time consuming process and requires sophisticated instrumentation.

### Carbon fiber micro electrodes (CFMEs)

Mark Wightman’s lab pioneered the use of carbon fiber microelectrodes for fast-scan cyclic voltammetry to measure changes in dopamine concentration and to correlate them with behavior. CFMEs have become an important type of electrode for neurotransmitter-based electrochemistry because their diameters are small enough to limit tissue damage during insertion. They also possess acceptably high sensitivity for small concentration changes and undergo fast electron transfer kinetics [45–48]. There are two common methods to fabricate CFME’s. The traditional tapered glass microelectrodes are made by inserting a single carbon fiber into a capillary, heating and pulling the capillary to form two tapered capillaries, to fuse the glass onto the fiber, and sealing any remaining space between the fiber and the glass with epoxy. At this point, the carbon fibers are trimmed and polished to be flushed with the glass/epoxy insulation for a disk microelectrode, or trimmed to extend beyond the glass-seal for a cylindrical microelectrode. Fibers that cannot withstand the force and/or the temperature applied during the capillary pulling phase can be fabricated into electrodes by a gentler but more time-consuming method. The fiber is manually inserted into a small inner-diameter and polymer-coated fused silica capillary as typically used for capillary electrophoresis. One end of the fiber is sealed to the capillary with epoxy, taking care to ensure that the carbon fiber extends to the desired length and that the exposed fiber is not coated with epoxy. If a disk electrode is preferred, the capillary can then be polished to ensure a smooth electrode surface [31]. A schematic is given in Fig. 2γ.

CFMEs allows for a variety of advantages. The small diameter of the carbon fiber (about 7 μm) makes them ideal for in vivo measurements with fast scan cyclic voltammetry (FSCV). When penetrated into a pulled glass capillary, stimulated neurotransmitter release in different brain regions and in brain slices (with very less tissue damage) can be detected. Also with FSCV, the carbon fibers of relatively high conductivity can measure fast (subsecond) transient changes of neurotransmitters in the brain. They have an extremely good temporal resolution with low sensitivities. However, the glass insulation of CMFEs can pose problems. A possible disadvantage could be related to the glass insulation of CFMEs. Though glass has been used for over 20 years in construction of CFMEs, it could potentially break or shatter, thereby causing damage to the animal or the tissue sample in question. Thus alternative insulations are being researched. Moreover, on using FSCV a high background capacitance is obtained. This reduces the signal to noise ratios, thereby requiring further data processing to distinguish between blank and analyte signals. Even though CFMEs are extremely sensitive, they are not very selective in nature.

### Graphene based electrodes

Graphene, a two-dimensional (2-D) nanomaterial with a hexagonal lattice of sp^2^ Carbon atoms, is well known for its outstanding properties including a large surface-to-volume area (2630 m^2^ · g^-1^, which is double that of single-walled carbon nanotubes (SWCNTs)), high electrical conductivity and fast adsorption kinetics, is an ideal material for analytical applications [25]. The subtle electronic properties of graphene suggest that it has the ability to promote electron transfer when used as the working electrode [27, 49, 50]. The synthesis of graphene can be performed by bottom-up or top down strategies leading to graphene materials of different quality. The bottom-up approach is mainly performed by chemical vapor deposition of methane on metal substrates. The resulting material can be described as single layer of graphene with low numbers on defects. Drawbacks of this method are the low amount of material which can be synthesized and the difficulties in further proceeding of the carbon nanomaterial. Both are overcome by a top-down synthesis by chemical oxidation or electrochemical exfoliation of bulk graphite. The product, graphene oxide, comprises of many defects in the sp^2^ lattice. The presence of defects within the graphene structure is not particularly deleterious, since it is well known that heterogeneous electron transfer in the electrochemistry of sp^2^ carbons occur at the edges and defects, rather than at the basal plane of graphene sheets. Graphene oxide has a structure that is not fully planar because the sp^2^ carbon network is heavily damaged. In fact, the oxygen-containing groups of graphene oxide may be applied towards functionalization of biomolecules for biorecognition events during biosensing. Graphene oxide can be chemically, thermally or electrochemically reduced. Reduced graphene oxide (rGO) has a partially restored sp^2^ lattice, while also containing oxygen-bearing groups [50, 51]. These nanostructed graphene materials are then mixed into a carbon paste or deposited onto the solid electrode as a modifier in order to enhance the sensitivity of the detection platform [23, 52–55].

### Chemically modified electrodes (CMEs)

All the electrodes mentioned in the aforementioned section (Types of carbon-based electrodes) may be easily modified so to obtain chemically modified electrodes (CMEs). These have modified surfaces that serve different electrochemical functions. At a CME, a redox-active analyte transfers electrons to the electrode or vice versa [56]. As suggested by Murray et al. [57], CMEs can be subdivided into four categories based on the nature of the modification process. (a) CMEs with monomolecular layers are fabricated by formation of self assembled monolayers (SAM) onto the electrode surface by chemical bonding; (b) CMEs also can be modified via formation of a covalent bond between electrode and electroactive reagent, either by a synthetic route or by controlling the oxidation/reduction potentials in a suitable medium. (c) CMEs also can be modified with films of redox polymers, ion exchange polymers (viz. Nafion^tm^), electrically conducting polymers, crown ethers, complexing agents, electrostatic modification etc. (d) Modified carbon paste electrodes can be obtained by coating them with nanomaterials such as metallic nanoparticles, carbon nanotubes, graphene, metal complexes, clay, or with macrocyclic chemical compounds [23, 58–61]. A review on CMEs was published in 2003 [62], and the group of Hao have reviewed the use of conducting polymer composites with graphene for use in chemical sensors and biosensors [63]. In general, modification of the electrode surface can improve the selectivity of electrodes, improve resistance to fouling, assist in the preconcentration of species, improve electrocatalytic properties, and limit the access of interfering agents. A list of review articles dealing with CMEs are provided in Table S1 of Electronic Supporting Material (ESM).

## Supporting electrolytes in voltammetry

A supporting electrolyte, in electrochemistry, according to an IUPAC definition, includes electrolyte containing chemical species that are not electroactive (within the range of potentials used for the analysis) and which have ionic strength and conductivity that are much larger than in the absence of an ionic additive. Supporting electrolyte is also sometimes referred to as inert electrolyte or inactive electrolyte. They are widely used in electrochemical measurements where control of electrode potentials is required. This is done to increase the conductivity of the solution (to eliminate the so-called IR drop), to eliminate the transport of electroactive species by ion migration under the electric field, to maintain a constant ionic strength, to maintain a constant pH, and most importantly to eliminate migration current. The usual concentration range for supporting electrolytes is 0.05-1.0 M. In aqueous solutions, supporting electrolytes are commonly solutions of inorganic salts, acids, buffers, etc., e.g., KCl, KNO_3_, HCl, NaOH etc. In organic media, tetraalkylammonium and perchlorate salts are often employed. For convenience, we have categorized supporting electrolytes (commonly used in voltammetry) according to the operating pH:

- pH 1.0 (or below): HCl; H_2_SO_4_; HNO_3_
- pH 2.0 : HCl/NaCl; HCl/KCl; citric acid/HCl/NaCl
- pH 3.0: Citric acid/NaOH/NaCl; potassium hydrogen phthalate/HCl
- pH 4.0: Acetate buffer; potassium hydrogen phthalate; citric acid/NaOH
- pH 5.0: Acetate buffer; potassium hydrogen phthalate/NaOH; citric acid/NaOH
- pH 6.0: Acetate buffer (up to pH 5.75); phosphate buffer; citrate/phosphate buffer; Tris buffer; citric acid/NaOH
- pH 7.0: Phosphate buffer; Tris buffer; HEPES buffer; citric acid/NaOH
- pH 8.0: Phosphate buffer (till pH 7.8); borate buffer; HEPES buffer
- pH 9.0: Borate buffer; sodium tetraborate/HCl
- pH 10.0: Borate buffer

In general, for buffers which contain citric acid, the pH range is from 2.1 to 7.4; dihydrogen phosphate (pH range: 5.8-7.8); acetic acid (pH range 3.75-5.75). Britton Robinson buffer (B.R. buffer) or ‘Universal Buffer’ can be employed from pH 2.0 to pH 12.0. Universal buffers consist of mixtures of acids of diminishing strength (increasing pK_a_) so that the change in pH is approximately proportional to the amount of alkali added. Two kinds of buffer were introduced by Britton and Robinson:

- A buffer being 40 mM in boric acid, 40 mM in phosphoric acid, and 40 mM in acetic acid and titrated to the desired pH with 0.2 M sodium hydroxide.
- A buffer being 28.6 mM in citric acid, 28.6 mM in KH_2_PO_4_, 28.6 mM in boric acid, 28.6 mM in veronal, and 28.6 mM hydrochloric acid and then titrated to the desired pH value with 0.2 M NaOH. This formulation gives an essentially linear pH response to added alkali from pH 2.5 to pH 9.2 (and in buffers to pH 12).

## Properties, electrochemical reaction mechanisms for ATC code N drugs and neurotransmitters

The key physicochemical properties of drugs and neurotransmitters within ATC code group N are summarized in Table S2 in the Electronic Supporting Material (ESM). All these drugs and neurotransmitters undergo electrochemical reaction that can be exploited to detect them by electroanalytical methods as outlined in the next section (Electrochemical detection of drugs and neurotransmitters based on ATC codes). Detailed electrochemical reaction mechanisms, as far as known, have been summarized in the ESM (Table S3).

## Electrochemical detection of drugs and neurotransmitters based on ATC codes

In this section we evaluate the detection of drugs belonging to Anatomical Therapeutic Classification (ATC) code N (groups N01 to N07) and neurotransmitters. This section classifies the work for each code N drug in terms of the electrode modifiers, voltammetric techniques, biological matrices, and detection systems.

### ATC code N01: anesthetics

ATC code N01 covers a therapeutic subgroup of the ATC classification system dealing with anesthetics. An anesthetic is a drug that causes a reversible loss of sensation. These are in contrast to analgesics (painkillers), which relieve pain without eliminating sensation. These drugs are generally administered to facilitate surgery. A wide variety of drugs are used in modern anesthetic practice. Many are rarely used outside of anesthesia, although a few others are used commonly by all disciplines. Anesthetics are categorized into two classes: general anesthetics, which cause a reversible loss of consciousness, and local anesthetics, which cause a reversible loss of sensation for a limited region of the body while maintaining consciousness. Combinations of anesthetics are sometimes used for their synergistic and additive therapeutic effects. A literature survey on electroanalytical methods for drugs of this code is given in Table S4 in the Electronic Supporting Material.

#### Procaine

**Fig. 3:**
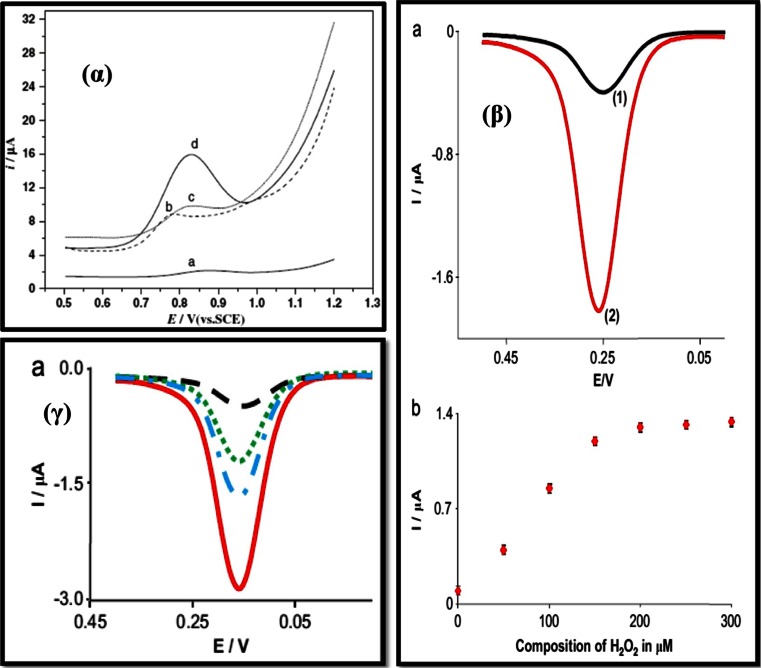
(α) Differential pulse voltammograms of procaine hydrochloride in pH 7.0 phosphate buffer solution at different electrodes: (a) GCE, (b) MWNTs/GCE, (c) PASA/GCE and (d) PASA/MWNTs/GCE. Pulse amplitude: 50 mV, pulse width: 50 ms, accumulation potential: 0.6 V and accumulation time: 120 s [65]. (β) (a) Square wave voltammetric response for dopamine obtained at 1-GCPE (1) without H_2_O_2_ and (2) with H_2_O_2_. (b) A plot for the effect of concentration of H_2_O_2_ on the peak current of DA at1-GCPE employing SWV [66] (γ) Adsorptive stripping square wave voltammograms of dopamine at GCPE (black); AgNPs-GCPE (green); 1-GCPE (blue) and 1-AgNPs-GCPE [66]

Procaine (PRO) [4-Amino-N-(2-diethylaminoethyl) benzamide], is a local anaesthetic derived from 4-aminobenzoic acid, which was first synthesized in 1905. PRO produces a state of local anesthesia by reversibly blocking the nerve conductance that transmits the feeling of pain and is widely used in the form of injections as well as a local application to the mucous membrane. Electrochemical determination of PRO using a carbon nanotube film coated electrode was successfully carried out [64]. Dihexadecyl hydrogen phosphate (DHP) was employed to disperse the MWCNTs and also to form a stable film onto the electrode surface. The limit of detection obtained by this method was 0.2 μM, which is not very sensitive. A glassy carbon electrode was coated [65] with a composite film containing electropolymerized poly (amidosulfonic acid) (PASA) and MWCNT. PASA was used to disperse the nanotubes, similar to that of DHP [64]. The electrochemical behavior of PRO was studied by DPV. Fig. 3α presents the typical DPVs of PRO in pH 7.0 solution at different electrodes. At the bare GCE (Fig. 3α, curve a), the oxidative peak current was relatively weak and occured at a peak potential of 0.876 V. PRO showed better electrochemical response and a high current at the MWCNTs/GCE (Fig. 3α, curve b) with the peak potential of 0.784 V and a current density of 41.4 μA cm^−2^. This indicates that MWCNTs have an electrocatalytic effect on the oxidation of PRO. At the PASA/GCE (Fig. 3α, curve c), the PRO also exhibited better electrochemical response than that on bare GCE. This was due to the presence of a high concentration of negatively charged SO_3_^−^ functional groups in the PASA film, which facilitate the electron transfer between electrode and PRO. The current density at the PASA/MWNTs/GCE (Fig. 3α, curve d) was the highest. This illustrated that the current response increment was caused not only by the increment of catalytic surface area but also by faster electron transfer kinetics. The LOD obtained by this method was 25 nM, which is much lower than that of ref. [64].

In both of these prior investigations, it is noteworthy that interferences by ascorbic and uric acid to the analysis of PRO were significant when interfering species were present at levels in excess of a few-fold higher concentration than PRO. This indicates a compromised selectivity within both these methods, which is likely to be especially significant if applied to the determination of PRO in biological fluids.

#### Capsaicin

Hot or spicy peppers and chilies, as well as herbaceous plants belonging to the genus *Capsicum* are popular food additives [capsaicin, CAP] that are widely utilized in many parts of the world. They are currently available as various topical pharmaceutical forms (ointments, high-dose dermal patches, creams, large bandages) for a number of diverse clinical conditions such as pain relief for peripheral neuropathy, symptomatic treatment of arthritis, muscle and joint pains, or *Herpes zoster*. They are used in the treatment of obesity and ulcer in the forms of oral herbal supplements. They are also the active ingredient in riot control and personal defense pepper spray chemical agents. Furthermore, it was demonstrated that CAP induces sapoptosis in various cancer cells. However, studies are available showing that CAP can cause a 2-5 fold increase in the incidence of stomach, pharynx, esophagus and larynx cancer in countries where it is highly consumed. Total capsaicinoid content of heat-producing chemicals is one of the main parameters that determine its commercial quality, and it is directly related to the heat (pungency) of the level of a pepper. This level often is expressed in so-called Scoville heat units (SHU) that is determined by an organoleptic method referred to as Scoville organoleptic Test [67]. Few electrochemical methods do exist.

A CNT-based sensor was developed [68] for quantifying the heat of chili peppers by adsorptive stripping voltammetry using a planar pyrolytic graphite electrode. The LOD was 0.31 μM. This method offered advantages such as precision and objectivity over the well-known, but potentially subjective Scoville method. It was also facile and inexpensive, in comparison to the existing HPLC methods. AdSDPV-based determination of CAP also was demonstrated [69] with a disposable pencil graphite electrode in buffer of pH 9. The LOD obtained was 3.7 nM, which is much lower than the one of ref. [68]. This method was employed for the analysis of CAP in pepper flakes. However, it has to be noted that the pH values of the supporting electrolyte in these methods are almost at two extremes of pH scale viz., pH 1.0 [68] and pH 9.0 [69]. Since these pH values are away from the physiological pH value, the analysis of extremely low concentration of CAP in biological fluids will be difficult by these methods.

### ATC code N2: analgesics

Analgesic drugs act in various ways on the peripheral and central nervous systems. They are distinct from anesthetics, which reversibly eliminate sensation. Analgesics are widely used and include paracetamol (known in the US as acetaminophen), the non-steroidal anti-inflammatory drugs such as the salicylates, and opioid drugs such as morphine and opium. A brief review of this class of drugs is covered in Table S5 in the Electronic Supporting Material. The methods for determination of the single analgetics are then treated in some detail.

#### Paracetamol

Paracetamol (acetaminophen, N-acetyl-p-aminophenol, PCT) is widely used as an antipyretic and analgesic drug. It is remarkably safe in standard doses; however, due to its wide availability, deliberate or accidental overdoses are not uncommon. Recent studies have shown that PCT is associated with hepatic toxicity and renal failure despite its apparent innocuous character [70]. Representative examples of methods for their electrochemical determination are reviewed below.

##### Carbon nanotubes and graphene

**Fig. 4:**
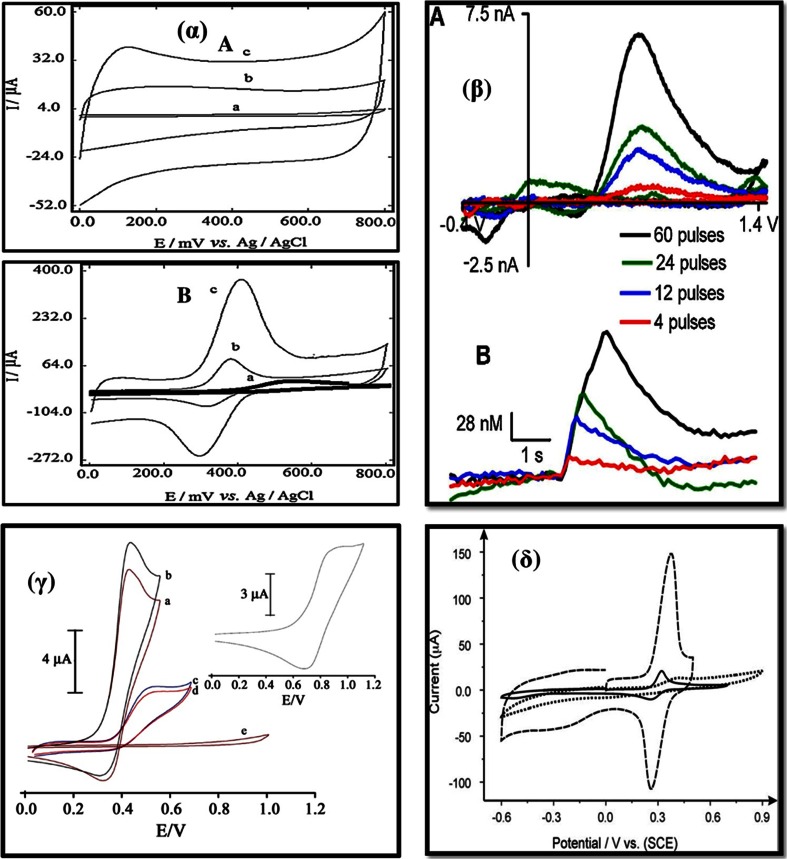
(α) CV obtained for the (A) blank (pH 7.0 phosphate buffer) and (B) acetaminophen: (a) bare GCE, (b) MWCNT and (c) P4VP/MWCNT GCE in 0.1 M phosphate buffer (pH 7.0) with a scan rate 20 mVs^−1^ [74]. (β) Dopamine detection in vivo at an epoxy-insulated microelectrode. (A) CVs depicting stimulated dopamine release detected from an Armstrong epoxy-insulated CFME placed in the caudate putamen with a stimulation pulse train of 60, 24, 12, and 4 pulses, respectively. (B) The associated current vs. concentration plots. The electrode was scanned from -0.4 to 1.45 V and back at 400 V/s at 10 Hz [31]. (γ) Cyclic voltammograms of phosphate buffer (pH 7.0) at a scan rate of 20 mVs^−1^ in the presence of morphine at VFc/CPE (a). (b) is as (a) at VFc/MWCNT/CPE. (c) is as (b) and (d) is as (a) at CNT/CPE and at CPE, respectively. (e) is as (a) without mediator. Inset: cyclic voltammogram of VFc/MWCNT/CPE in 0.1 M phosphate buffer (pH 7.0) at a scan rate of 20 mV s^−^[79]. (δ) Cyclic voltammograms for paracetamol in phosphate buffer of pH 7.5 on MWCNT-BPPGE (dashed line), bare BPPGE (dotted line) and EPPGE (solid line). Scan rate: 100 mVs^−1^ [71]

**Fig. 5:**
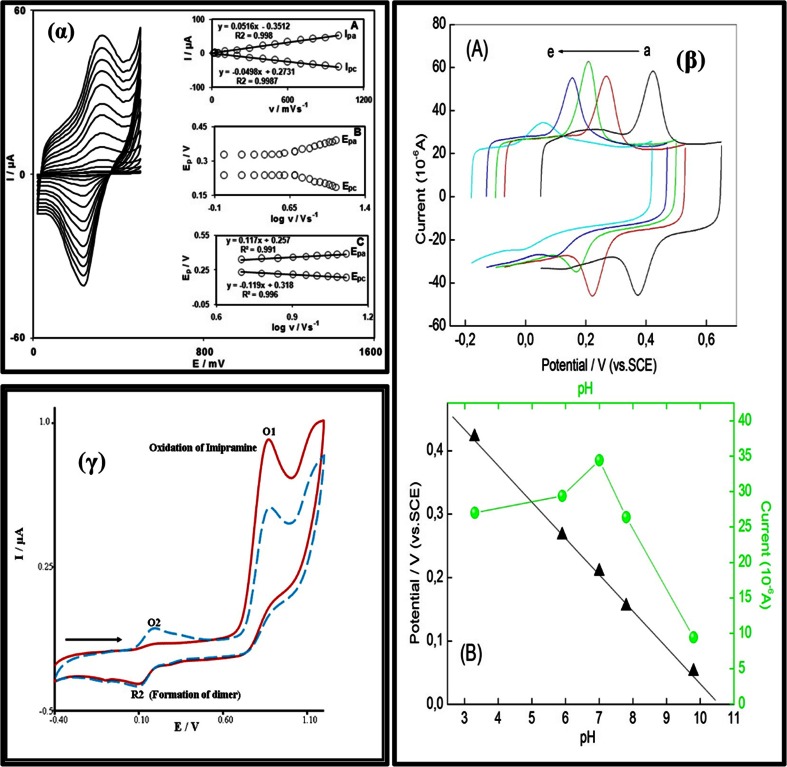
(α) Cyclic voltammograms of MC-CNPE in phosphate buffer (pH 7.0), at various scan rates, from inner to outer, 10-1000 mV s^-1^. Insets: variation of (A) I_p_ vs. scan rate; (B) Variation of E_p_ vs. the logarithm of scan rates; (C) Variation of E_p_ vs. the logarithm of the high scan rates [81]. (β) (A) CV curves of CNT/PGE in phosphate buffer containing L-dopa at different pH: a → e, 3.3, 5.9, 7.0, 7.8, and 9.8; (B) dependence of anodic peak potential (black triangle) and anodic peak current (green globular) as a function of solution pH in phosphate buffer containing L-dopa [82]. (γ) Two repeated CV scans for IMI at GCPE in phosphate buffer (pH 6.0). First scan (—) and second scan (- - -) at a scan rate of 100 mV s^-1^ [83]

Several articles discuss the employment of MWCNT composite electrodes for analysis of PCT [71–77]. Compton’s group [71] developed a sensitive AdSSWV method for PCT at a MWCNT modified basal planar pyrolytic graphite electrode. As can be seen from the CV plot [Fig. 4δ], the ratio of the faradaic peak current for oxidation and reduction of paracetamol to the background capacitive current is much larger than in case of a bare electrode. This suggests that the adsorption of paracetamol on MWCNTs is much stronger and results in a detection limit of 45 nM. The authors further analyzed PCT in tablets containing paracetamol, aspirin and caffeine, however, a simultaneous determination of these three molecules was not carried out. Also, the size of the electrode is 4.9 mm in diameter, and thus this electrode cannot be used in real time analysis of PCT in cerebral tissues. The effect of pH and scan rates are shown in Fig. 5 and discussed subsequently.

**Fig. 6:**
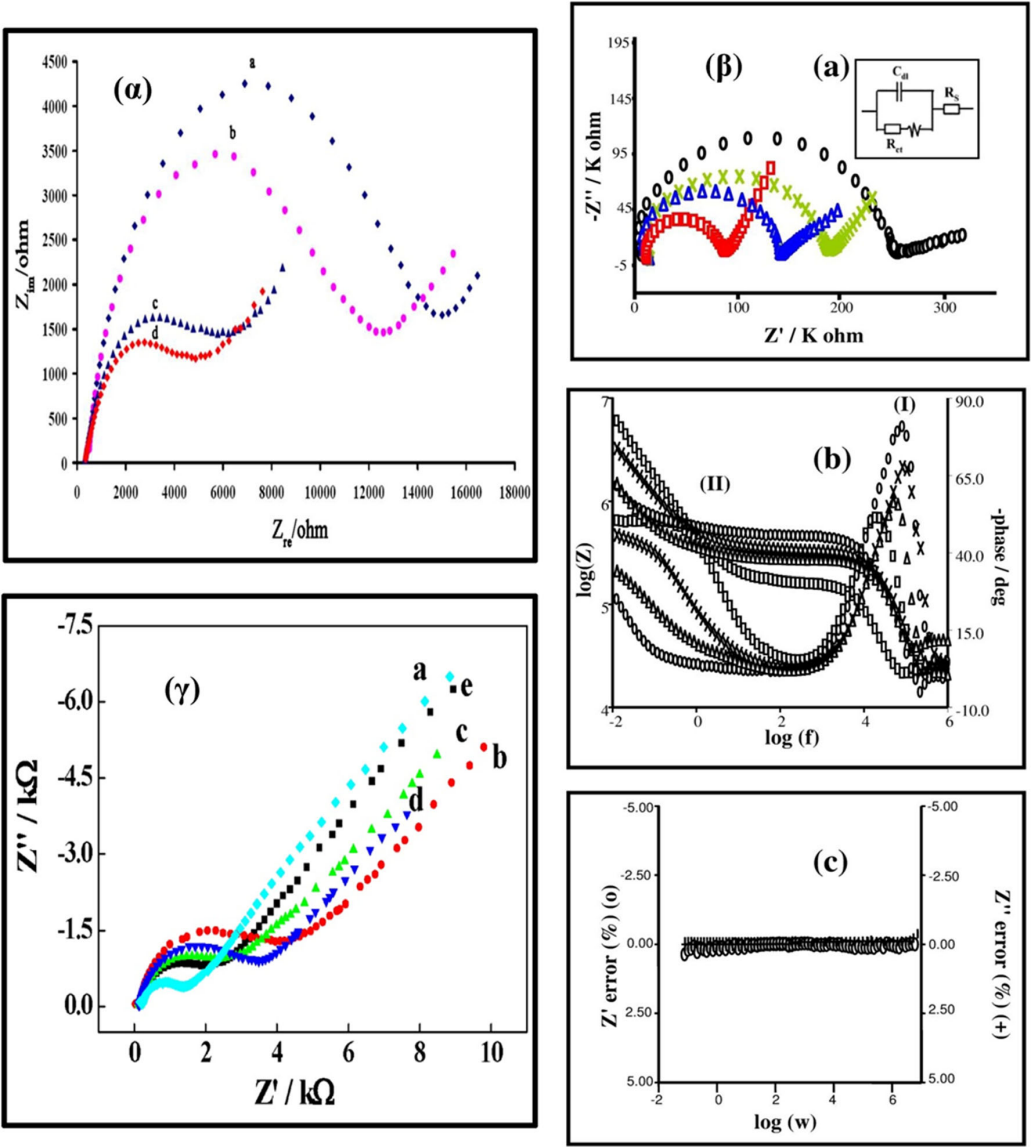
(α) Nyquist plots of CPE (a), MWCNTPE (b), CPILE (c), and MWCNT-CILE in the presence of morphine [84]. (β) (a) Nyquist plots for EIS measurements at PCPE (ooo), ISSM − PE (× × ×), CNT − PE (ΔΔΔ) and ISSM − CNT − PE (□□□). In the box on the right upper side is the equivalent circuit used for data fitting. (b) Bode plots: (i) logarithmic plot of frequency vs. impedance and (ii) logarithmic plot of frequency vs. phase angle at the PCPE (ooo), the ISSM − PE (× × ×), the CNT − PE (ΔΔΔ) and the ISSM − CNT − PE (000). (c) Kramers -Kronig transformation test (plot of log ω vs. error in Z^1^ or Z^11^) for paracetamol at the ISSM – CNT-PE in phosphate buffer solution (pH 7.0) containing Triton × 100. The amplitude of the perturbation was 10 mV and the frequency range was from 10^−2^ Hz to 10^6^ Hz [72]. (γ) Complex impedance plots measured in Fe (CN)_6_^3−^/Fe (CN)_6_^4−^ + KCl + phosphate buffer solution (pH 7.0) at formal potential of 0.14 V for (a) the bare GC electrode; (b) the poly (SFR)/GC electrode; (c) the nano-Au/poly (SFR)/GC electrode; (d) the DNA/nano-Au/poly (SFR)/GC electrode; and (e) the nano-Au/DNA/nano-Au/poly (SFR)/GC electrode. The frequency range is between 10^−2^ Hz and 10^6^ Hz with signal amplitude of 50 mV [85]

**Fig. 7:**
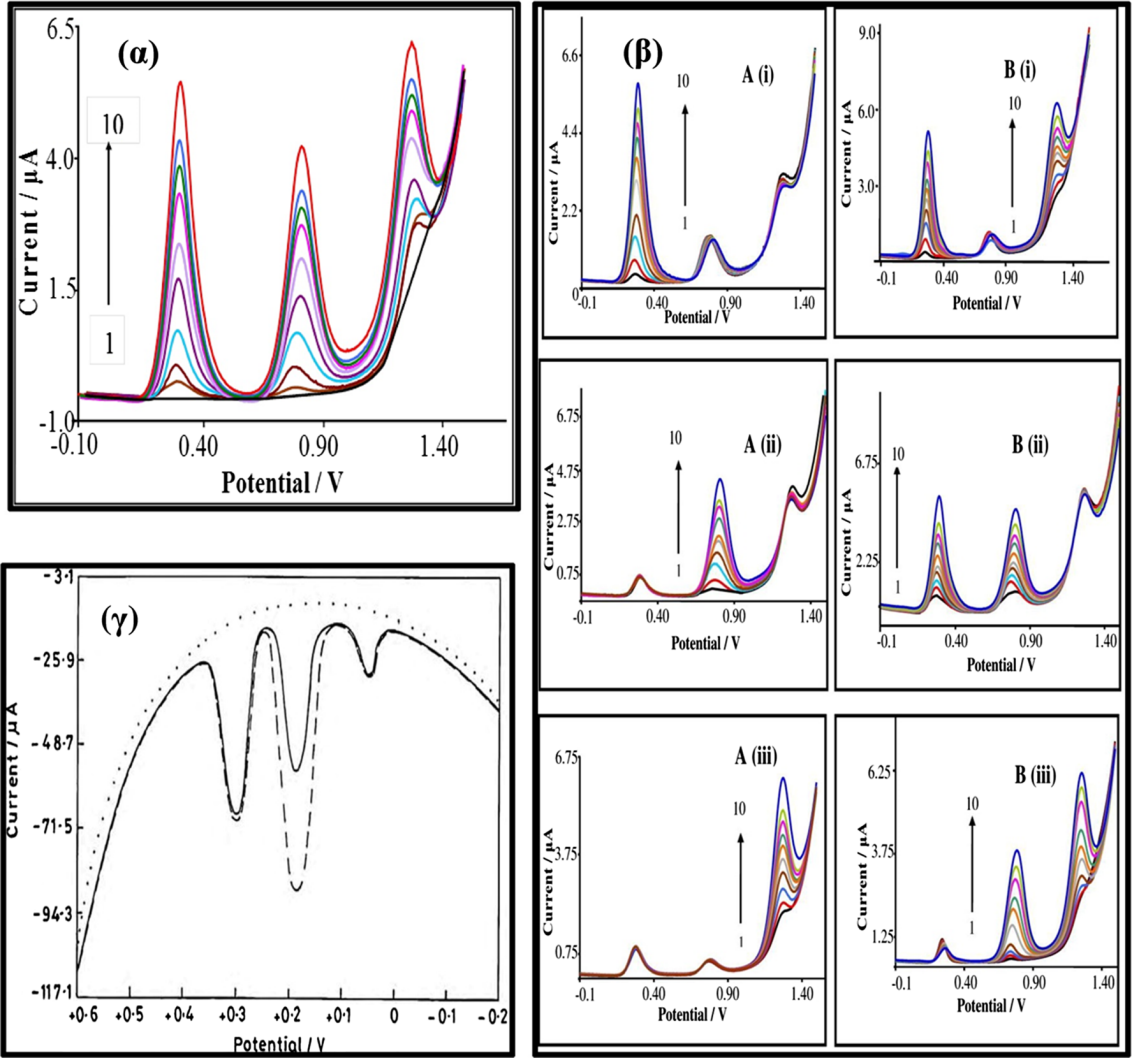
(α) AdSDPV curves obtained for the oxidation of paracetamol, aspirin and caffeine at equal concentrations in the range of (1) blank to (10) 62.7 μM for each molecule [72]. (β) (A) (i) AdSDPV curves obtained at the ISSM-CNT-PE for paracetamol at different concentrations in the presence of 50 μM aspirin and caffeine: (1) 0.143 to (10) 65.3 μM. (ii) AdSDPV curves obtained at the ISSM-CNT-PE for aspirin at different concentrations in the presence of 4.14 μM paracetamol and caffeine: (1) 0.28 to (10) 64.2 μM. (iii) AdSDPV curves obtained at the ISSM-CNT-PE for caffeine at different concentrations in the presence of 42 μM paracetamol and aspirin: (1) 0.27 to (10) 65 μM. (β) (B) (i) AdSDPV curves obtained at the ISSM-CNT-PE for paracetamol and caffeine at different concentrations in the presence of 3.9 μM aspirin: (1) 0.285 to (10) 64.3 μM. (ii) AdSDPV curves obtained at the ISSM-CNT-PE for paracetamol and aspirin at different concentrations in the presence of 25 μM caffeine: (1) 0.277 to (10) 64 μM. (iii) AdSDPV curves obtained at the ISSM-CNT-PE for aspirin and caffeine at different concentrations in the presence of 5 μM paracetamol: (1) 0.281to (10) 64.8 μM [72]. (γ) A comparison of voltammograms observed for urine sample of patient being treated with paracetamol (—) and the patient sample spiked with paracetamol (- - -) at pH 7.2 at SWNT modified EPPGE. Background is represented as (. . .) [78]

Single walled carbon nanotubes (SWCNT) modified edge plane pyrolytic graphite electrode (EPPGE) has been used by Gupta’s group [78] for voltammetric determination of PCT. The sensitivity at the SWCNT modified EPPGE used was 2 times better than that of a MWCNT-modified EPPGE. The method was then applied to the determination of the drug in human urine samples obtained after 4 h of administration of PCT. As can be seen from Fig. 7γ, the blank gave no signal. However, when the urine sample of a patient who was treated with PCT was taken for analysis, three distinct peaks were obtained. A typical square wave voltammogram of urine sample at SWCNT/EPPGE is represented in Fig. 7γ. A well-defined peak of paracetamol was noticed at E_p_ ca. 187 mV. Authors further state that the other voltammetric peaks at ca. 300 mV and ca. 50 mV may be estimated to be due to the presence of uric acid and ascorbic acid respectively in the urine sample. The urine sample of the patient was then spiked with a known concentration of paracetamol. The voltammogram clearly depicts that the peak current increases significantly for the peak at E_p_ ca. 187 mV, thereby confirming that it corresponds to the oxidation of paracetamol. This figure also shows that this method [78] can be employed for simultaneous determination of ascorbic acid, PCT and uric acid. A very low LOD (2.9 nM) was obtained for PCT.

Surfactants have also been employed to modify carbon nanotube paste electrodes. Sanghavi and Srivastava [72] have carried out simultaneous voltammetric determination of acetaminophen, aspirin and caffeine using an in situ surfactant-modified multiwalled carbon nanotube paste electrode. Triton × 100 (Triton 100) was employed as a surfactant facilitated adsorption of PCT onto the electrode surface. A high AdSDPV signal was obtained for PCT as compared to a bare carbon paste electrode. The observed increase in peak current was attributed to the presence of Triton 100, which has hydrophobic C-H chains and hydrophilic head groups that adsorbed strongly at the surface of the carbon paste, thereby enabling the adsorption of PCT. Consequently, the oxidation current of PCT was increased. Moreover, PCT was solubilized by Triton 100, thereby increasing its residence time near the electrode surface, since TX-100 forms a thin layer on the electrode surface, into which PCT is pre-concentrated. This phenomenon facilitated the enhanced kinetics of electron transfer between the electrode and PCT. As a result, the oxidation peak potential is shifted to a less positive value. Triton 100 was employed at an optimal concentration of 50 μM. This was well below the critical micellar concentration CMC (300 μM) of Triton 100. Beyond this concentration and due to the micelle effect [80] the peak current is decreased.

Four electrodes were investigated in an attempt to clarify the differences between their electrochemical performance. These comprised (a) a plain carbon paste electrode (PCPE), (b) an in-situ surfactant modified carbon paste electrode (ISSM/CPE), (c) a CNT/CPE, and (d) an ISSM/CNT/CPE. Electrochemical impedance spectroscopy (EIS) was employed as a technique for the electrochemical characterization of each electrode surface [Fig. 6]. As such, the Nyquist plots for PCT showed significant differences in the responses of the four electrodes, as shown in Fig. 6β (a). In addition, a semicircle with a large diameter was observed for the PCPE in the frequency range 10^−2^-10^6^ Hz. However, the diameter of the semicircle diminished when the ISSM-CNT-PE was employed. This observation implied that the charge transfer resistance of the electrode surface decreased and that the charge transfer rate increased upon employing the ISSM-CNT-PE. A Warburg impedance profile at 45° was also observed for all the electrodes of interest. The Warburg diffusion element is a common diffusion circuit element that can be used to model semi-infinite linear diffusion, that is, unrestricted diffusion to a large planar electrode. A Warburg impedance element can be difficult to recognize because it is nearly always associated with a charge-transfer resistance and a double layer capacitance, but is common in many systems. The Warburg diffusion element (Z_W_) is a constant phase element (CPE), with a constant phase of 45° (phase independent of frequency) and with a magnitude inversely proportional to the square root of the frequency.

The authors further carried out electrochemical characterization of the electrode surface using a Bode plot [Fig. 6β (b)]. In the absence of Triton 100, only a single, symmetrical peak existed on the Bode plot [phase angle (˚) vs. log frequency (f)], which corresponded to the relaxation process of the electrode/solution interface, as given in Fig. 6β (b). However, the Bode plot was very different when 5 × 10^−5^ M Triton 100 was present, showing two well-defined peaks that correspond to the two relaxation processes, (I) and (II). The adsorption of surfactants at high concentrations on hydrophobic surface yielded a monolayer. Due to the formation of this monolayer, the smoothness of the surface of the surfactant modified electrodes increased in comparison to the unmodified ones. As the relaxation of the electrolyte solution usually occurred at high frequencies, relaxation (I) on the curve was attributed to the electrolyte solution, and relaxation (II) to the monolayer of Triton 100 on the electrode surface. It was noted that for those electrodes, in which there was no surfactant present, the second relaxation was not observed. In addition, the ISSM-CNT-PE showed a lower relaxation than the ISSM-PE electrode, implying that the reaction was more facile on the former electrode.

This conclusion was supported by another type of Bode plot that compared log Z vs. log f. In the frequency range of relaxation (I) a plot of log Z vs. log f gave a slope of -0.5, which corresponded to the Warburg impedance associated with the electrolyte solution. As for relaxation (II), the slope for log Z vs. log f was -0.55, which indicated the presence of a constant phase element in the relaxation of the Triton 100 monolayer. The dependence of log Z on log f in the absence of Triton 100 had a slope of -0.8, corresponding to the capacitance of the electric double layer. A slope of almost zero at the two extremes represented the capacitance of the electric double layer as well as the two resistances associated with the electrolyte solution and the charge transfer between the solution and the electrode. Finally, the Kramers–Kronig transformation test was carried out on PCT to test the validity of the impedance data [Fig. 6β (c)]. The Kramers–Kronig transformation gave a *χ* ^2^ (chi square) of 3.48 × 10^−6^ for PCT. Therefore, the system satisfied all the conditions for a good impedance data (i.e., linearity, causality, stability and finiteness of the system).

The authors then carried out simultaneous determination of PCT, aspirin and caffeine in three stages [Fig. 7 (α) and (β)] (a) Keeping the concentration of two molecules constant and varying the concentration of the third; (b) Keeping the concentration of one molecule constant and simultaneously varying the concentration of the other two and (c) varying the concentration of all the three molecules simultaneously. PCT, aspirin and caffeine were successfully quantified, but the LODs were not lower than Compton’s electrode [71]. We have mentioned this study of electrochemical impedance spectroscopy in more details because in almost all papers dealing with chemically modified electrodes, only Nyquist plots have been studied by authors. Bode plots and Kramer Kronig transformation tests are also very important and thus should more often be performed to understand special electrode processes. However, it is observed that both these important tests have been avoided.

Dispersing CNTs is a challenge in itself. Some authors have overcome this problem by wrapping CNTs with conducting polymers which improves the electrical, physical, and conductive properties of the electrodes. For example, a GCE was modified [74] with a composite film of poly (4-vinylpyridine) (P4VP) and multiwalled carbon nanotubes (P4VP/MWCNT/GCE) for the determination of PCT. The anodic peak currents of PCT on the P4VP/MWCNT/GCE were about 300-fold higher than that of the un-modified GCE. The bare GCE [Fig. 4α A (a)], in the absence of PCT, gave a very low capacitive current. However, this current increased for the MWCNT/GCE [Fig. 4α A (b)] and even more so for the P4VP/MWCNT/GCE [Fig. 4α A (c)]. This indicates that the effective surface area of the P4VP/MWCNT/GCE is larger than that of the MWNT GCE. The electrochemical response to PCT at three different electrodes was studied by using cyclic voltammetry (Fig. 4 αB). The broad redox peaks of PCT at the bare GCE and the MWCNT modified GCE [Fig. 4α B (a) and (b)] indicated a sluggish rate for electron transfer. However, the P4VP/MWCNT-glassy carbon electrode displayed well-defined redox peaks with the peak separation (ΔE_p_) of 97 mV which indicated a favorable quasi-reversible electrode process [Fig. 4α B (c)].

Electrochemical determination of PCT also was demonstrated [75] by using a GCE modified with multi-wall carbon nanotubes dispersed in polyhistidine. Similar to the P4VP work done by Ghani [74], the authors employed polyhistidine as a conducting polymer to disperse CNTs. Simultaneous determination of paracetamol with ascorbic acid was demonstrated. Such polymer-coated electrodes hold much promise towards detecting nM levels of PCT. However, the polymer coating is likely to slow down the diffusion towards the electrode surface, thereby lowering the temporal resolution.

Graphene (GR) has attracted intensive interests in recent years since its discovery by Geim and coworkers in 2004 [86], owing to its large specific surface area, high thermal and electrical conductivities, great mechanical strength, and potentially low manufacturing cost. GR-based nanocomposites as materials for fabricating electrochemical and other sensors have received increased attention, because these kinds of nanocomposites increase the electrocatalytic activity and thus enhance the sensitivity of the sensors [23, 87–89]. A graphene-modified MWCNT/GCE has been employed [76] for the voltammetric determination of PCT. This method was further extended towards a simultaneous determination of PCT and tyrosine in tablets and serum samples. The LOD obtained for PCT was 0.1 μM, which indicates that the method is not sensitive. Also, ascorbic and uric acid interfered in a few-fold excess in concentration, which means that there will be selectivity problems if this sensor has to be employed for analysis of PCT in biological fluids. Recently, Luo et al. [90] developed an electrochemical sensor for PCT based on a composite consisting of graphene oxide and a molecularly imprinted sol–gel polymer (GO/MIPs) through one-pot room temperature polymerization. AA, UA and 4-NP did not interfere with the analysis of PCT which implies that the proposed method is selective in nature. However, LOD of 20 nM is obtained which indicates that the method is not as sensitive as compared to Gupta [78] electrode which has a 10 fold lower LOD as compared to Luo electrode [90].

##### Metallic nanoparticles

Metal oxide and metal hydroxide nanoparticle composite electrodes have also been employed for determination of PCT [91–95]. For example, a composite made from TiO_2_, graphene and nafion (Nafion/TiO_2_-GR/GCE served [92] for the determination of PCT. Incorporation of TiO_2_ nanoparticles along with graphene enhanced the electrochemical reactivity and voltammetric response to PCT. In addition, Nafion acted as an effective solubilizing agent and antifouling coating in the fabrication of the modified electrode. On the bare GCE, PCT showed an irreversible redox behavior with small and undefined redox peaks. The enhanced voltammetric response to PCT on the Nafion/TiO_2_-GR/GCE was attributed to the large specific surface area and electrocatalytic activity of graphene, which improved the adsorption efficiency and electrochemical reactivity of PCT. The adsorbed TiO_2_ nanoparticles modified the surface chemistry of graphene sheets, thus providing an efficient interface and microenvironment for the electrochemical reaction of PCT. The LOD using this method was 0.21 μM, which is moderate.

Ding et al.[93] developed an electrochemical sensor based on a GCE modified with LaNi_0.5_-Ti_0.5_O_3_/CoFe_2_O_4_ nanoparticles (LNT–CFO/GCE) for determination of PCT. Lower overvoltage for oxidation of PCT and a larger faradic current was obtained at the LNT-CFO/GCE, which indicated that LNT–CFO nanoparticles promoted the oxidation of PCT. The oxidation of PCT to N-acetyl-p-benzoquinone-imine was catalyzed by the La[Ni^III^Ni^II^] x [Ti^V^Ti^III^] _1−x_O_3−y_ in phosphate-buffered saline. In the heterogeneous reactions that occur with electron transfer on perovskite-type oxides, the active sites are the transition metal ions with partially occupied d orbitals. The perovskite-type oxides LNT–CFO may be considered as catalysts with transition metals in mixed oxidation states and with the general formula La [Ni^III^Ni^II^] x[-Ti^V^Ti^III^] _1−x_O_3−y_, where y stands for oxygen vacancies. A moderate LOD of 0.19 μM was obtained, which is poorer than that of other electrochemical sensors. Moreover, the authors have carried out amperometry at a potential of +0.5 V, which implies that ascorbic and uric acid will interfere in the analysis of PCT, thereby making this method less selective.

It can be seen that MWCNT-modified electrodes have been employed vastly for determination of PCT. Moreover, the polymer modification of MWCNT electrode generally gives lower LODs for PCT as compared to other modifications. Recently, Pumera et al.[96] state that CNT-based electrodes are better for p-aminophenol (metabolite of PCT) oxidation as compared to other electrodes because of the carbonaceous impurities (consisting of amorphous carbon and nanographite) present within them. The authors showed the metallic impurities to have no influence to the electrochemistry of p-aminophenol. Instead, its electrochemical behavior at CNTs was governed by the carbonaceous impurities (nanographite, amorphous carbon) contained in the CNTs. The intrinsic nature of CNTs generated no electrocatalytic effect towards the redox behaviour of p-aminophenol.

#### Aspirin

Aspirin, the common name for acetylsalicylic acid (ASA), is one of the oldest medicines but still plays an important role in therapeutics. It is widely employed for the relief of headaches, fever, muscular pain, and inflammation due to arthritis or injury. Due to its high consumption there is a need to develop analytical methods to assess not only the quality but also the authenticity of a given product.

It is known that ASA can be indirectly determined after its conversion to salicylic and acetic acid. The voltammetric peak obtained thereby is due to the presence of salicylic acid [72, 95]. The mechanism for the reaction is given in Table S3 in the Supporting Electronic Material. These methods are time consuming since they require prior hydrolysis. The advantage of these methods is that once hydrolysis of ASA occurs, its determination can be easily carried out at physiological pH values.

Literature also reports a few methods where the electrochemical determination has been performed without prior hydrolysis of aspirin to salicylic acid. Square wave voltammetric determination of ASA in pharmaceutical formulations using a boron-doped diamond (BDD) electrode was carried out [97] in 10 mM sulfuric acid as the supporting electrolyte. The rate of decomposition of ASA to salicylic acid and acetic acid is dependent on solution pH. In the range of pH 11-12, ASA is quickly hydrolyzed, whereas in the range of pH 4-8, its hydrolysis is better controlled and maximum stability is attained at pH 2-3 [98]. BDD electrodes are preferred over glassy-carbon or platinum electrodes, from the viewpoint of greater stability to corrosion in the acidic and alkaline media. BDD electrodes also enable low and stable background currents, high response sensitivity, and a very wide working potential window of ~3.5 V. A detection limit of 2 μM was obtained by this method, which indicates that the method is not very sensitive. Moreover, since authors have worked at pH 2 this method will have limited utility for analysis of ASA directly biological fluids.

A multiwalled carbon nanotube-alumina-coated silica (MWCNT-ACS) nanocomposite was used [99] to modify a glassy carbon electrode (GCE) for determination of ASA. The study reports that the electrocatalytic activity of this electrode causes a higher current response and lower oxidation potential for detection of ASA. ACS assisted in the dispersion of the MWCTs on the GCE. This resulted in a 100-fold higher sensitivity in comparison to a bare GCE. The limit of detection for ASA is 3.77 μM which however is not really low. Similar to the BDD electrode [97], the authors employed dilute sulfuric acid as a supporting electrolyte. Even though these methods are very fast and do not require prior hydrolysis of aspirin to salicylic acid, a supporting electrolyte of extremely low pH has to be employed, which makes the use of these methods unlikely for determination of ASA in biological fluids. However, the mechanism by which acetyl salicylic acid gives a voltammetric peak (at extremely acidic pH values) needs investigation.

#### Morphine

Morphine (MOR) is a potent opiate analgesic medication and considered to be the prototypical opioid, which can cause disruption in the central nervous system. It is used to relieve severe pain in patients, especially those undergoing a surgical procedure and works by dulling the pain perception center in the brain. Morphine is a precursor in the manufacture of a large number of opioids such as dihydromorphine, hydromorphone, nicomorphine, heroin and codeine. MOR is in a group of drugs called narcotic pain relievers. It is a benzylisoquinoline alkaloid with two additional ring closures. Short-acting formulations are taken as needed for pain. To date, many analytical methods have been developed. A comparison of some of the voltammetric methods is presented below.

Atta’s group [7] developed an AuNP and Nafion-modified CPE for voltammetric determination of MOR. AuNPs enhanced the peak current of MOR due to a higher electroactive surface area, and by increasing the electron transfer kinetics. MOR has a pK_a_ value of 8.08. Nafion is a cation exchange polymer. The supporting electrolyte employed in this work was pH 7.4, where MOR is present as a cation and thereby binds to Nafion. This causes an enhancement in peak current due to more accumulation of MOR on the electrodes surface. A rather low LOD of 1.33 nM was obtained in standard solution. However, the LOD of MOR in urine sample was 87.2 nM, which indicates that this method is not selective due to the interferences present in urine matrix.

The ionic liquid (IL), 1-butyl-3-methylimidazolium hexafluoro phosphate and MWCNTs on a CPE were employed [84] for the determination of MOR. The IL plays the dual role of a binder as well as a modifier. EIS studies indicated that the charge transfer resistance decreased drastically at the IL-MWCNT-modified CPE. Fig. 6α shows the EIS of different modified electrodes in the presence of MOR. On the traditional CPE, the value of R_ct_ was higher (curve a), than that at MWCNT/CPE (Fig. 6α, curve b). Addition of IL to form carbon IL electrode (CILE) further reduced the charge transfer resistance (Fig. 6α, curve c). The lowest R_ct_ value was obtained at MWCNT and CILE modifiers (Fig. 6α, curve d). As a result, the LOD for MO was 0.14 μM, with a wide LWR from 0.45 to 450 μM. However, interference studies within biological fluids were not carried out. In another method [100], an N-hexyl-3-methylimidazolium/MWCNT-modified GCE was employed for the voltammetric determination of MOR in urine and injection sample. This sensor had an LOD of 20 nM. However, authors had spiked μM concentration of MOR in biological fluids, which suggests that probably this method is not sensitive to MOR at nM concentration levels in real biological samples.

GCEs were also modified [101] with MWCNT and a chitosan composite to enable simultaneous determination of L-dopa and MOR. Chitosan, a biocompatible polymer, was employed for improving the adherence of the MWCNTs onto the GCE. The detection limit for L-dopa is 0.86 μM, while that for MOR is 0.65 μM. This sensor was further employed for analysis of both these molecules in blood serum and urine samples. However, an 11 % decrease in the peak current was obtained after 36 h of operation.

A GCE modified with graphene nanosheets (GNS) was applied [102] to the simultaneous determination of MOR, noscapine and heroin. The DPVs obtained for a mixture of these three molecules gave rather low signal levels, with the peaks for noscapine and heroin, as a broad unresolved hump. However, upon modification of the GCE with GNS, the peak current was enhanced and a clear cut separation was seen between the peaks for MOR, noscapine and heroin. Furthermore, the electrochemical response of the modified electrode towards morphine, heroine and noscapine was independent of each other. Simultaneous detection of heroine, morphine and noscapine without any separation or pretreatment was carried out. Since the electro-oxidation peak at 0.85 V corresponds to both morphine and heroin, the authors determined the amount of heroin by calculating morphine concentration from its relevant peak at 0.37 V and then by deducing the obtained value from the value calculated for the peak observed at 0.85 V. The sensitivity was not very high, as indicated by LODs of 0.4, 0.2 and 0.5 μM for MOR, noscapine and heroin, respectively.

**Fig. 8:**
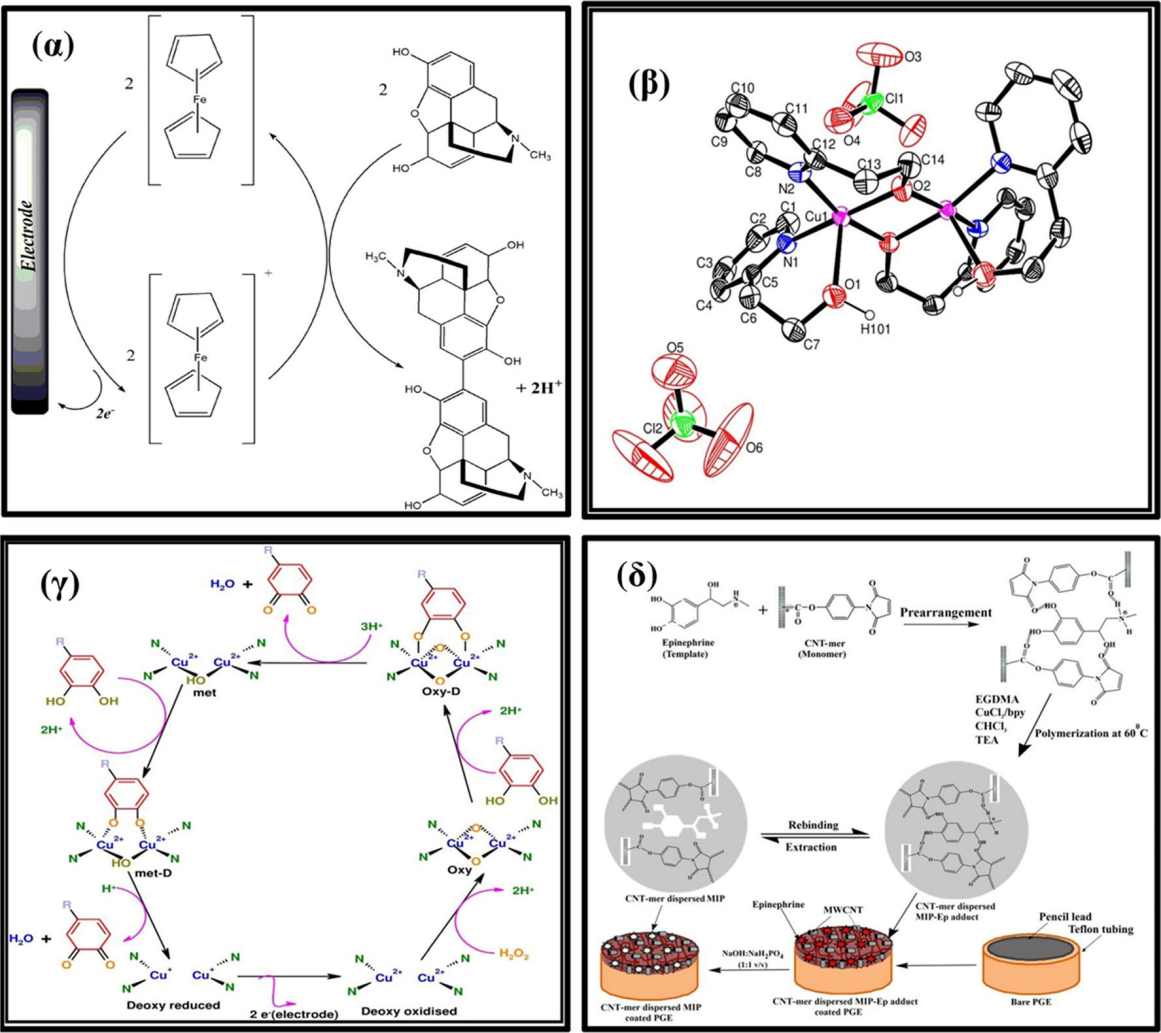
(α) Suggested response mechanism of the sensor based on vinylferrocene, for the catalytic electro-oxidation of morphine [79] (β) Single crystal XRD of the biomimetic copper complex [66]. (γ) Mechanism for Tyrosinase in which the oxy species is formed from Cu_2_^4+^ and H_2_O_2_. Here, D implies DOPA bound forms [66]. (δ) Suggested binding mechanism of Epinephrine in their respective MIP cavities via weak non-covalent interactions [132]

A carbon paste electrode modified with vinylferrocene and multiwalled carbon nanotubes (VFc/MWCNT/CPE) was fabricated [79] for determination of MOR. The vinylferrocene acts as a mediator that facilitates the oxidation process [Fig. 8α]. In this process, the electrochemically formed Fc^+^ reacts with MOR diffusing toward the electrode surface. At the same time, simultaneous oxidation of the regenerated Fc caused an increase in the anodic current. Fig. 4γ depicts the cyclic voltammetric response for the electrochemical oxidation of MOR at VFc/CPE (curve a), VFc/MWCNT/CPE (curve b), CNT/CPE (curve c), and CPE (curve d). The anodic peak potential for the VFc/MWCNT/CPE (curve b) and VFc/CPE (curve a) were about 420 mV, while at the CNT/CPE (curve c) the peak potential is about 500 mV. It was concluded that the best electrocatalytic effect for morphine oxidation was observed at the VFc/MWCNT/CPE (curve b). The LOD was 90 nM, indicating that the method is adequately sensitive for MOR determination. However, cysteine, acetaminophen and cystine interfered, not very good selective nature of the electrode.

#### Tramadol

Tramadol (TRA) is a centrally acting analgesic, which possesses opioid properties and activates mono-aminoergic spinal inhibition of pain. Like morphine, TRA binds to receptors in the brain (opioid receptors) and inhibits reuptake of norepinephrine and serotonin, which appears to contribute to the drug’s analgesic effect. Many clinical studies have evaluated the therapeutic efficacy (analgesic effects) of TRA in comparison with morphine and other analgesics and found Tramadol to be effective for relief of postoperative pain (patient-controlled analgesic, PCA), moderate surgical pain, surgical pain in children, cancer pain control, obstetric pain, osteosynthesis and chronic pain. Numerous electroanalytical methods are known for its determination.

A CNT-modified GCE was further modified [103] with mono-dispersed gold nanoparticles (AuNP/CNT/GCE). Fig. 8α shows a schematic illustration of the sensor fabrication process. Prior to modification, the GCE was polished with alumina water slurry, sonicated in ethanol, washing with distilled water, and dried on air. For the preparation of the CNTs/GCE the CNTs were dispersed in ethanol and then dropcast onto the surface of a GCE. For preparation of AuNPs/CNTs/GCE the CNTs/GCE was placed in a solution containing HAuCl_4_, and the AuNPs were electrochemically deposited by applying a potential of -0.4 V (vs. Ag/AgCl) for 4 min. The AuNP/CNT hybrid increased the electroactive surface area and enhanced the electron-transfer between the electrode and the analyte. The modified electrode gave an LOD of 68 nM for TRA. A GCE was modified [104] with carbon nanoparticles for simultaneous voltammetric determination of TRA and acetaminophen. An LOD of 1 μM was obtained by this method which is moderate. Interference by ascorbic and uric acids on the peak currents of TRA was not investigated and thus selectivity cannot be commented upon.

Sanghavi and Srivastava [105] employed a GCP modified with Dowex and gold nanoparticles for a simultaneous voltammetric determination of acetaminophen and TRA in pH 6 buffer using AdSSWV. Acetaminophen and TRA have pK_a_ values of 9.50 and 9.41 respectively, and both exist in cationic form at pH 6. Due to this, they bind to the Dowex resin, causing their accumulation onto the electrode surface. The method allowed their simultaneous determination in the working range of 33.4 nM to 42.2 nM with detection limits of 4.71 nM and 11.2 nM respectively, indicating that this method is more sensitive in comparison to the method of Shahrokhian et al. [104]. Ascorbic acid (pK_a_ 4.17) and uric acid (pK_a_ 5.75) exist in anionic form at pH 6.0 and were hence repelled from the electrode surface. Thus, this method exhibits both sensitivity and selectivity. The field of employing resins as modifiers has not been explored widely, though, despite the beneficial effect on selectivity.

#### Sumatriptan

Sumatriptan (SUM) is a highly selective 5-hydroxytryptamine (5-HT) receptor agonist. It is a triptan drug, which is effectively used in the treatment of migraine and cluster headache attacks. There are two models that explain the mechanism of action of this agonist. One theory suggests that SUM selectively binds to serotonin type-1 day receptors and activates them, which results in vasoconstriction of extensively dilated cranial blood vessels and subsequent relief of headache. The other theory suggests that SUM exerts its effect by inhibiting the release of neurogenic inflammatory mediators. SUM is administered in several dosage forms including products for nasal, oral and rectal delivery. Numerous electro-analytical methods have been reported.

The response to SUM at a bare pyrolytic graphite electrode (PGE) is poor and only a weak oxidation peak was observed at 0.75 V. If, however, voltammetric studies are performed [106] with a PGM modified with MWCNT and silver nanoparticles (AgNPs-MWCNT/PGE), the current obtained at the AgNPs-MWCNT/PGE is significantly larger than that obtained for the bare PGE, probably reflecting the larger electroactive surface area of the modified electrode. A detection limit of 40 nM was obtained by this method. However, no interference studies with either ascorbic acid or uric acid were carried out. In other work [107], a GCE modified with MWCNT and polypyrrole (PPy) doped with new coccine (NC) was employed for sensitive electrochemical determination of SUM. During the growth of PPy on the electrode surface by electropolymerization, NC anions participate in the polymerization to balance out positive charges produced by the oxidation process. The use of NC as a dopant caused homogeneous polymer growth with increased current on sequential scans to yield an efficient and stable film on the electrode. In this manner, the aromatic structure of NC and its effective π–π interactions with the polymer, as well as with the MWCNTs backbone, served as an anionic dopant during the electro-deposition process, thereby improving polymer loading on the surface of CNT/GCE. The LOD for SUM is 6 nM, which indicates the high-sensitivity of the method.

Recently, Srivastava’s group [108] developed a sensor for SUM based on nano-modification. The authors electrochemically deposited AuNP and graphene onto the glassy carbon electrode surface after which they further carried out a nafion modification. These modifiers enhanced the electrochemical properties of the sensor. The LOD for SUM obtained by the Srivastava electrode was 0.7 nM which is 10 fold lower than the Shahrokhian electrode [107] which implies that the method is extremely sensitive as compared to any other electrochemical method for SUM determination. Authors carried out the selectivity studies for SUM in presence of AA (pKa = 4.17), UA (pKa = 5.7), indole-3-acetic acid, indole-3-pyruvic acid, indole-3-lactic acid and 5-hydroxy indole-3-acetic acid (pKa between 3.0 to 4.7) which are most common interfering agents present along with SUM in biological samples. At pH 7.0 PBS (supporting electrolyte), all these molecules were repelled from the electrode surface by nafion and thus did not interfere with the analysis of SUM. Thus this method having a better sensitive as well as good selective as compared to other methods for SUM determination. A common drawback for all the SUM electrodes is a relatively large size of the electrode which would restrict applications for in-vivo measurements.

#### Codeine

Codeine (3-methylmorphine; COD) is an analgesic and antitussive agent that belongs to the family of opiates naturally found in the poppy plant. COD is widely available as a single agent or in combinations with other analgesics or miscellaneous drugs such as acetylsalicylic acid, ibuprofen and caffeine in pharmaceutical tablets to reduce pain and pyrexia. Moreover, COD is extensively used in cough and cold syrup. It also can cause drug addiction and mental damage if abused, and then may give rise to social problem.

Electro-analytical methods for COD are scarce. A sensor was reported [109] for the determination of COD at MWCNT/GCE. Simultaneous determination of COD was carried out with paracetamol. The detection limit was 0.19 μM. A rapid and sensitive electrochemical method for determination of COD in pharmaceutical formulations and human urine [110] is making use of a BDD film electrode. A very low detection limit (80 nM) was obtained, which is lower than that reported in ref. [109]. Ascorbic acid, uric acid and glucose did not interfere with the analysis of COD, hinting towards a good selectivity of the method.

Voltammetric determination of COD using single-walled carbon nanotubes modified carbon-ceramic electrode was carried out [111] and showed an LOD of 110 nM. This is not better than that of ref.[110]. However, simultaneous determination of COD and caffeine in pepsi, cola and tea samples was demonstrated.[111]

#### Benorilate

Benorilate (BEN) is an esterification product of paracetamol and acetylsalicylic acid in order to put two different compounds together possessing the same activity. It belongs to a class of medicines called non-steroidal anti-inflammatory drugs. It works by blocking the production of chemicals (prostaglandins), which the body produces in response to injury or certain diseases. These prostaglandins would otherwise go on to cause swelling, pain and inflammation. The drug has also analgesic and antipyretic properties, and helps reduce fever and discomfort in viral illnesses like flu. Benorilate is probably absorbed as the intact molecule, which accounts for its good gastric tolerance. After absorption, benorilate is hydrolyzed into its components, salicylate and acetaminophen, which then follows the usual routes of metabolism.

Differential pulse voltammetry for determination of BEN in pharmaceutical formulations was carried out with a CPE [112] in pH 6.9 solution with a CPE at a level of 50 nM. The analysis of BEN was also carried out in pharmaceutical formulations. In the same year, determination of BEN in pharmaceutical formulations and in urine was carried out by the same group [113] using a CPE modified with silver nanoparticles. The AgNPs enhance the surface area of the electrode and the electron transfer kinetics, and this resulted in an LOD of 10 nM (versus 50 nM with an un-modified CPE). This method was extended further towards the determination of salicylic acid, a metabolite of BEN.

### ATC code N03: anti-epileptics

The anti-convulsants (also known as anti-epileptic drugs) form a diverse group of pharmaceuticals that fall under ATC code N03 and are used in the treatment of epileptic seizures. Anticonvulsants are also increasingly being used in the treatment of bipolar disorder, since many seem to act as mood stabilizers, and for the treatment of neuropathic pain. The goal of an anticonvulsant is to suppress the rapid and excessive firing of neurons that start a seizure. Anticonvulsants are more accurately called antiepileptic drugs (abbreviated “AEDs”), and are sometimes referred to as antiseizure drugs. Several antiepileptic drugs have multiple or uncertain mechanisms of action. The methods for electrochemical analysis are summarized in Table S6 in the Electronic Supporting Material.

#### Carbamazepine

Carbamazepine (CBZ) is a highly lipophilic neutral tricyclic compound. CBZ, as one of the most widely used antiepileptic drugs, is successfully prescribed in the treatment of psychomotor, generalized tonic–clonic seizures and complex partial seizures. In a voltammetric method [114] for analysis of CBZ, dihexadecyl hydrogen phosphate was used as a dispersing agent to obtain a homogeneous dispersion of MWCNTs and to form a uniform and stable MWCNT film on the surface of a GCE. The LOD of the method was 40 nM. Diclofenac and clofibric acid were the strongest interfering agents for determination of CBZ. An electrochemical sensor based on fullerene-C_60_-modified GCE was fabricated [115] for the determination of CBZ. The fullerene acts as an electron mediator. The electro oxidation and reduction of CBZ occurred at a lower potential and with enhanced peak current at the fullerene-modified GCE compared to a bare GCE. Moreover, the oxidation peaks were sharper and well defined compared to those observed at a bare GCE. The LOD was 16.2 nM which makes it more sensitive than that of ref.[114].

#### Gabapentin

Gabapentin [GBP; 1-(aminomethyl) cyclohexane acetic acid; trade name Neurontin] is a new antiepileptic drug that is used for the treatment of partial onset seizures with or without secondary generalized tonic–clonic convulsions. Gabapentin is also effective in the prevention of frequent migraine headaches, neuropathic pain, and nystagmus and treatment of nerve pain caused by herpes virus or shingles.

GBP was determined [116] with a silver nanoparticles-modified MWCNT paste electrode in pH 10 solution. The AgNPs acted as a mediator. The anodic peak current increased after addition of increasing amounts of GBP, which indicated the suitability of the electrode for determination of GBP in the solution. The reaction occurred as follows:

$$ \begin{array}{l} A g\to A{g}^{+}+{e}^{-}\hfill \\ {} A{g}^{+}+ nGBP\to A g{(GBP)_n}^{+}\hfill \end{array} $$

After addition of GBP, the reaction led to the formation of an Ag^+^/GBP-complex. The LOD obtained for GBP was 0.56 nM, which indicates excellent sensitivity. However, the need for pH 10 reduces the applicability of this method for analysis of GBP in biological fluids.

Determination of GBP with a CPE modified with nanotubes of nickel oxide was carried out [117] using amperometry at +0.67 V and employing 0.1 M NaOH as the supporting electrolyte. Nickel oxide (NiO) nanotubes were found to enhance the surface area and thus to increase sensitivity. They also act as a mediator in the electron transfer process. NiO nanotubes were transformed to Ni(OH)_2_ by hydration on exposure of the electrode surface to the solution. Then, the modifier was oxidized back and forth from Ni(OH)_2_ to NiOOH by potential cycling. In the presence of GBP, the anodic current and the associated anodic charge increased dramatically, whereas the cathodic current and the corresponding charge decreased. The LOD was 0.3 μM, which is less than in Yari’s method [116]. Also, since authors have employed a potential of +0.67 V to carry out amperometry, it is likely that ascorbic acid, uric acid and dopamine will interfere with the analysis of GBP, since their oxidation potentials are lower than the applied potential.

#### Lamotrigine

Lamotrigine (lamictal, LMT) is a well-known anticonvulsant (a medication for treating epilepsy). In addition, LMT has been approved by the U.S. food and drug administration (FDA) for the treatment of bipolar disorder. By decreasing electrical conduction or neurotransmitter activity in unstable brain cells, anticonvulsants play an effective role in controlling seizures and bipolar illness. In the case of overdoses, the most serious side effect of LMT is to cause life-threatening skin rashes including a form called Stevens–Johnson syndrome, which is characterized by painful blistering of the skin and mucous membranes and often is fatal. Owing to the dangerous side effect of LMT, the pharmaceutical quality control of LMT is vital.

Determination of LMT by adsorptive stripping voltammetry was carried out [118] by using an AgNP-modified carbon screen-printed electrode, and the LOD obtained was 372 nM after 200 s of accumulation. LMT also was determined [119] with a pyrolytic graphite electrode and obtained a LOD of 90 nM (after 100 s of accumulation), which makes this method more sensitive. It was observed that AgNP modification gave extremely low sensitivity for this class of drugs, thus this option can be explored deeply for future research which involves the analysis of LMT not only by voltammetry but even by UV–Vis, HPLC etc.

### ATC code N04: anti-parkinsons and neurotransmitters

The antiparkinson, or antiparkinsonian drugs are intended to relieve the symptoms of Parkinson's disease or Parkinsonism. Most of these agents act by either increasing dopamine activity or by reducing acetylcholine activity in the central nervous system. We have included other neurotransmitters in this code (even though they do not fall directly under ATC Code N) since they are directly related to the nervous system. These are dopamine, epinephrine, norepinephrine and serototonin. The electrochemical methods employed for the analysis of this class of drugs are summarized in Table S7 in the Electronic Supporting Material.

#### Levodopa and carbidopa

Patients suffering from Parkinson’s disease have a significant depletion of dopamine (DA) in their brains. DA cannot be administered directly because this neurotransmitter cannot cross the blood–brain barrier into the central nervous system and cannot be employed to restore its normal level. Levodopa (L-dopa) (a precursor of DA) is an important neurotransmitter that is used for the medication of Parkinson’s disease. After administration, L-dopa is converted into DA through enzymatic reaction catalyzed by dopadecarboxylase.

For better therapeutic effect and lower toxicity, carbidopa (C-dopa) is administered in association with L-dopa. This catecholamine has an inhibitory effect on decarboxylase activity. Therefore, by administering L-dopa combined with C-dopa, the concentration of DA is adjusted to an appropriate level and this combination also results in reduction of side effects. The half-life of L-dopa is 50 min. However, if C-dopa is present along with it, its half-life increases to 1.5 h.

L-dopa was determined [120] with a glassy carbon electrode (GCE) whose surface was modified with multi-walled carbon nanotubes and polypyrrole (PPy) doped with tiron. The modified electrode showed a 7-fold enhancement in the peak current as compared to bare a GCE. Since polypyrrole was doped with tiron (an anionic species), it repels other anionic species, viz. ascorbic and uric acid from the electrode surface. The LOD for L-dopa is 0.1 μM which is moderate. While ascorbic and uric acid do not interfere, dopamine, the amines, epinephrine and norepinephrine which exist in cationic state at pH 7, are likely to interfere in the analysis of L-dopa. Hence, this sensor is unlikely to be applicable to the determination of L-dopa in cerebrospinal fluid samples.

A plain graphite electrode was covered with MWCNTs that were modified with gold nanoparticles.[82] Cyclic voltammetry was then carried out to characterize the effect of solution pH on redox peak potentials of L-dopa at this AuNP-MWCNTs/PGE. As shown in Fig. 5β, plot A, the redox peak potential of L-dopa shifted negatively with increasing solution pH, which indicated protons were involved in the electrode reaction process. The anodic peak potential of L-dopa was proportional to pH in the range of 3-10 [Fig. 5β, plot B]. The linear regression equation is E_pa_(V) = 0.606 − 0.057pH, and the slope is 57 mV which is almost Nernstian. The slope of the potential-pH equation demonstrated that an equal number of electrons and protons are involved in the reaction mechanism. pH study is an extremely crucial tool towards predicting the electrochemical redox process. Table S3 in the Electronic Supporting Material gives the electrochemical redox process of L-dopa. Using the differential pulse voltammetry for the accurate determination of LD, the oxidation current was proportional to concentration in the range of 0.1-150 μM, with a detection limit of about 50 nM.

A CPE also was modified with a bis (nitriloethylidyne)-bis-hydroquinone and carbon nanotubes to carry out the simultaneous determination of L-dopa, C-dopa, and tryptophan.[121] The LOD of the method is 94 nM. However, ascorbic acid, dopamine, epinephrine and norepinephrine all interfere. This renders the electrode less useful for analysis of biological fluids. Similarly, Khalilzadeh et al. [122] studied the electrochemical behavior of C-dopa at the surface of a CNT paste electrode modified with an ionic liquid. The oxidation peak potential appeared at 555 mV, which was about 68 mV lower than the oxidation peak potential at the surface of the conventional CPE. On the other hand, the oxidation peak current was increased to about three times. The LOD is around the same as that for basal C-dopa in biological fluids. However, the electrode needs to be miniaturized if it is to be employed for analysis of C-dopa. Finally, C-dopa was determined [123] with a ferrocene-modified carbon nanotube paste electrode. Here, enhancement in peak current was obtained by the dual effect of the nanotubes and ferrocene. The MWCNTs increases the surface area and improved electrical conductivity, while ferrocene acts as a redox mediator. Both L-dopa and ascorbate interfere and the limit of detection for CD is only 3.6 μM.

#### Dopamine

Dopamine (DA) is a neurotransmitter that belongs to the catecholamine group and plays an important role in the central nervous, renal, hormonal and cardiovascular systems. Neurotransmitters (NTs) are chemical messengers that transmit a message from one neuron to the next. This transmission proceeds by secretion of the NTs from one neuron, which then binds to the specific receptor that is placed on the membrane of the target cell. One of the important ways that neurons communicate is by interaction between the NT and the receptor. Consequently, some neurological disorders such as schizophrenia and Parkinson’s disease can be due to the abnormality of the dopaminergic system in the central nervous system. The first paper on DA detection using a MWCNT paste electrode was published by Ajayan et al. [124] as early as in 1996. Rivas group [125] was among the first groups to employ MWCNT in CPEs for determination of dopamine. We deeply thank authors of both these works for carving a new path for the future research. These articles have inspired many researchers to employ MWCNTs as a modifier in paste electrodes.

Fast scan cyclic voltammetry (FSCV) is an electrochemical technique that can be used to monitor the release and uptake dynamics of endogenous monoamine levels both in vitro and in vivo. Subsecond measurements of naturally occurring changes in the concentration of DA have been reviewed [126]. Venton’s group [31] fabricated a carbon-fiber microelectrode (CFME) using epoxy insulation for the quantitation of in-vivo stimulated dopamine release in anesthetized rats. The working electrode, an epoxy-insulated CFME, was placed in the caudate putamen. The electrode was allowed to equilibrate before stimulated dopamine release was measured. A biphasic stimulating electrode was placed in the substantia nigra to stimulate the dopaminergic cell bodies. Stimulation pulse trains were applied and the dopamine response recorded. Fig. 4β shows the response to DA in vivo for a variety of pulse trains. Current increased during stimulation as DA was released and decreased after the stimulation, due to uptake. The LOD was 24 nM. However, since the electrode had to be equilibrated for 1 hour, this method is time consuming. In other work by this group [127], an aligned CNT forest was prepared by a chemical self-assembly method, which resulted in more exposed CNT. Shortened, carboxy-functionalized single-walled CNTs were assembled from a dimethylformamide suspension onto a carbon-fiber disk microelectrode modified with an iron hydroxide-decorated Nafion film. The modified electrodes were highly sensitive, with 36-fold higher oxidation currents for DA using the FSCV rather than a bare electrode. The LOD for DA was 17 nM. Even though the FSCV method is sensitive, a high background capacitance is obtained. This reduces the signal to noise ratios, thereby requiring data processing to distinguish between blank and analyte signals.

Metallic nanoparticles have also been employed for determination of DA. A gold nanocluster (nano-Au) was incorporated into a GCE modified with a 3-amino-5-mercapto-1,2,4-triazole film and used [128] for the simultaneous DPV determination of ascorbic acid, dopamine, uric acid and nitrite at pH 4.0. The compound 3-amino-5-mercapto-1,2,4-triazole (TA) has five potential coordination sites, viz. three cyclic nitrogen atoms, one exocyclic amino group, and one exocyclic thiol group. A layer of poly-TA contains numerous thiol groups, which facilitate the modification of a gold film. A sensor was constructed by electropolymerizing TA and doping it with nano-Au. It was used for the simultaneous determination of AA, DA, UA and NO_2_^−^ at low levels. The nano-Au/p-TA modified sensor exhibits high electrocatalytic activities towards the oxidation of these molecules. This method was applied for the analysis of these molecules in spiked urine and serum samples.

A Pt/reduced graphene oxide (Pt/RGO) modified glassy carbon electrode was developed [129] for the detection of DA and uric acid in the presence of high concentration of ascorbic acid. A detection limit of 0.25 μM for DA indicates that the method is not sensitive as compared to other recent voltammetric methods for determination of DA (Table S7). Simultaneous determination of dopamine, uric acid and ascorbic acid was carried out [130] using LaFeO_3_ nanoparticles. The nanoparticles associated via hydrogen bonding to the hydroxy group of DA. This weakens the O-H bond energy and improves the electron transfer rate. At the same time, the high surface area of the LaFeO_3_ nanoparticles improves the response current of DA. An LOD of 30 nM was obtained, not a very sensitive method.

**Fig. 9:**
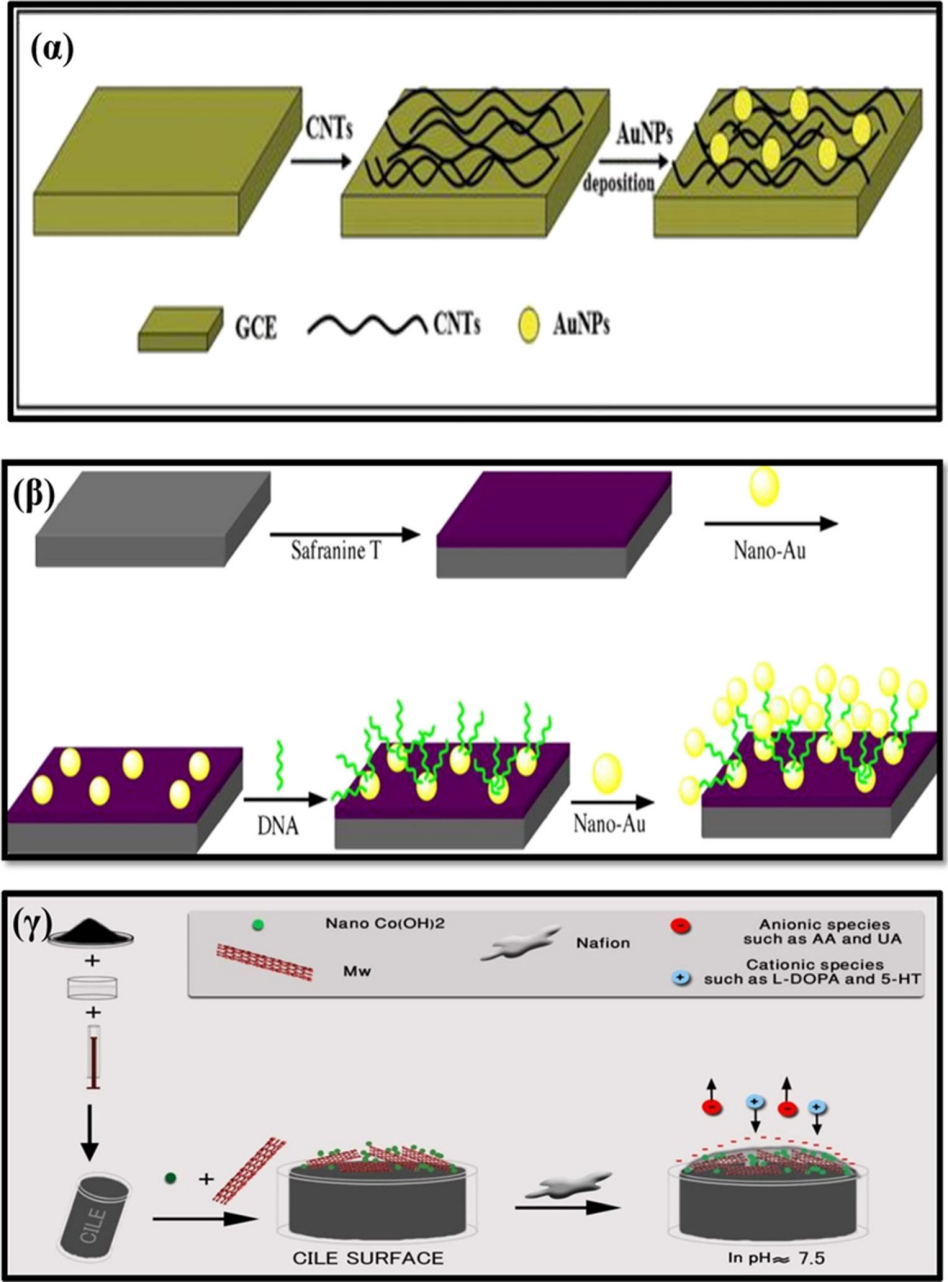
(α) Schematic illustration of the stepwise sensor fabrication process (electrodeposition) [103]. (β) Schematic illustration of the stepwise sensor fabrication process (immobilization) [85]. (γ) Schematic illustration of the stepwise sensor fabrication process (nafion coated electrode) [143]

DNA has also been employed for DA detection in synergism with AuNPs. A sensor based on a nano-Au/DNA/nano-Au/poly (SFR) composite for simultaneous determination of dopamine, uric acid, guanine, and adenine was reported [85]. A film of poly-safranine T (SFR) was deposited on a GCE surface by electropolymerization. The electrode was immersed into colloidal solutions of AuNPs which was then transferred into a ds-DNA solution, immersed and then rinsed with water and tris-buffer to remove non-adsorbed DNA. Finally, the electrode modified with three-dimensionally distributed AuNPs was obtained by re-immerse the DNA-modified electrode into a colloidal gold solution. The nano-Au/DNA/nano-Au/poly (SFR)/GCE is illustrated in Fig. 9β. The poly (SFR) monolayer not only produced a catalytic effect but also provided a template, onto which AuNPs were anchored, generating a 2D array of nano-gold-modified electrodes. The second layer of AuNPs on the electrode formed a 3D distribution via DNA linkages. Reaction rates were improved because of the combination of catalytic effects of poly (SFR) and sandwiched AuNPs. Impedance was measured at frequencies ranging from 10^-2^ to 10^6^ Hz and a formal potential of 0.14 V. A small semicircle was observed in the Nyquist plot of the bare GCE, as shown in Fig. 6γ (curve a). The interfacial R_ct_ corresponding to the semicircle diameters increased because the poly (SFR) perturbed the interfacial electron transfer rates between the electrode and the electrolyte solution (curve b). In the next step, electron transfer occurred more easily because the nanometer-sized AuNPs acted as conducting wires or electron-conducting tunnels. As a result, R_ct_ decreased after the adsorption of nanogold on the surface of the electrode (curve c). The increased R_ct_ observed after DNA immobilization was ascribed to the repulsion of negatively charged DNA phosphate skeletons. R_ct_ obviously decreased again after immersion into the colloidal gold solution for a second time, yielding a value lower than that of the AuNP monolayer-modified electrode. The LOD for DA was 0.2 nM which indicates that the method is sensitive. However, the electrode preparation was tedious and took almost 12 h.

The shapes of nanoparticles also have an effect on the electrochemical signal. Swamy et al. [131] developed a sensor for electrochemical determination of DA employing CuO nanoparticles that were either rod shaped or flake shaped. Both exhibited good electrocatalytic activity as compared to a bare CPE. Between the two types of CuO nanoparticles, the flake-shaped gave stronger current response (with a slight reduction of overpotential) than the rod-shaped. The electron transfer rate constants (k_s_) for DA at a GCE modified with the flake shaped and rod shaped CuO NPs were 0.55 and 0.43 s^-1^, respectively. The method was extended towards simultaneous determination of ascorbic acid and dopamine. However, authors did not study the effect of uric acid, which is often present in biological fluids and thus it is difficult to interpret whether the method is selective or no.

In a different approach, Srivastava’s group [66] developed a biomimetic sensor for dopamine employing a copper (II) complex (1) and a silver nanoparticle-modified GCPE (1-SNP-GCPE). The Cu (II) complex (1) [Fig. 8β], in the presence of hydrogen peroxide, initiates a cyclic process mimicking the enzyme tyrosinase, thereby enhancing the peak current of DA. Silver nanoparticles, on the other hand, exhibit high electrocatalytic activity towards the DA as compared to a bare electrode [Fig. 3γ]. Addition of hydrogen peroxide, prior to the addition of dopamine, can be explained considering the catalytic mechanism of tyrosinase from Cu_2_^4+^ plus H_2_O_2_ [Fig. 8γ]. Fig. 3β (a) shows the effect of presence and absence of H_2_O_2_ at 1-GCPE. In the absence of H_2_O_2_, a weak electrochemical signal was observed, while in the presence of H_2_O_2_ a good response for DA was obtained, suggesting that the peroxide is essential in the sensor response. The effect of peroxide concentration in pH 6 solution on the sensors’ response was examined [Fig. 3β(b)]. This indicates that peroxide is important chemical for the performance of this biomimetic sensor.

The mechanism for the interaction between 1 and catecholamines in the presence of H_2_O_2_ is presented in Fig. 8γ. In the first step, the hydrogen peroxide reacted directly with two Cu^2+^ ions, forming the oxy species necessary for catecholamine oxidation to form the quinone. In this stage, the copper centers are reduced to Cu^+^. A molecule of catecholamine is attached to two Cu^2+^ centers by losing two H^+^. This form was called the oxy-D form. The coordination of the catecholic substrate to the oxy-cupric form results in the breakdown of the complex with electron transfer from the catecholamine to oxygen, giving the corresponding ortho-quinone and water. This left the copper atoms at the active site in the oxidized state (Cu^2+^). In this state, they cannot bind H_2_O_2_ and therefore are inactive to catecholic substrates. This form was called the met form. However, this form was able to coordinate another molecule of catecholic substrate and oxidize it to the o-quinone by donation of electrons to copper atoms, generating the deoxy-reduced form (Cu_2_^2+^). Copper becomes electrochemically oxidized to complete the catalytic cycle. The two electrons left in the copper oxidation then can be used for oxygen reduction in the oxy species to form H_2_O and ortho-quinone. The two electrons thus generated, reduced the o-quinone back to catecholamine. This resulted in the completion of the cycle. A detection limit as low as 35 nM was obtained. This method was further extended towards determination of other neuro-transmitters viz., L-dopa, EPI and nor-EPI. Finally, the method was employed to the analysis of neurotransmitters in formulations, urine and blood serum samples. Another biomimetic sensor for DA was developed recently by Khan’s group [133] using a carbon fiber microbiosensor modified with copper-graphene oxide composite. Authors carried out the interference studies of DA in presence of AA and UA. However, the detection limit was 33 nM which indicates that the method is comparable in sensitivity as Srivastava electrode [66]. However, unlike [66], Khan’s group did not challenge the proposed biomimetic sensor for real sample analysis and thus it is difficult to say if the method can be employed in complex matrices. Even though both these biomimetic sensors are sensitive and the electrode is stable, the method involves addition of H_2_O_2_ to the electrochemical cell for carrying out catalysis. Thus, this sensor cannot be employed directly in living tissues for determination of catecholamines since H_2_O_2_ is a disinfectant and if comes in contact with body tissues; it can prove to be hazardous.

#### Epinephrine

Epinephrine (EPI; also known as adrenaline or adrenalin) is a hormone and a neurotransmitter. EPI has many functions in the body, regulating heart rate, blood vessel and air passage diameters, and metabolic shifts; EPI release is a crucial component of the fight-or-flight response of the sympathetic nervous system. In chemical terms, EPI belongs to a group of monoamines called the catecholamines. It is produced in certain neurons of the central nervous system and in the chromaffin cells of the adrenal medulla from the amino acids phenylalanine and tyrosine. Changes in the EPI concentration in nervous tissue and body fluids are diagnostic symptoms of several diseases. The amount of EPI present in blood, plasma or serum is considered as a diagnostic aid to monitor therapeutic administration or to identify the causative agent in potential poisoning victims. The quantitative determination of EPI concentration is also quite helpful for developing nerve physiology, diagnosis of certain diseases and in pharmacological research. Therefore, it is important to examine its electrochemical behavior and to develop a quantitative method for studying its concentration in body fluids. The various molecules which are present along with EP include dopamine, nor epinephrine, ascorbic acid and uric acid.

A MWCNT-modified EPPGE was demonstrated [134] to enable selective determination of EPI in smokers using SWV. The modified EPPGE exhibited catalytic activity towards the oxidation of EPI that leads to an increase in the current response and a shift of the oxidation potential to lower values as compared to a bare electrode. Simultaneous analysis of EPI was carried out with dopamine and nor-EPI. Ascorbic acid and uric acid did not have any effect. The LOD was 0.15 nM. This method was further employed for the analysis of EPI in blood plasma samples of smokers. However, the size of the electrode surface is very large and thus miniaturization is desirable.

Functionalized multiwalled carbon nanotubes were obtained [135] via in-situ electropolymerization of brilliant cresyl blue, and the resulting composite (PBCB-MWNTs-DHP) was applied in an electrode for the determination of EPI. The electrochemical response was improved due to the enhanced adsorption of EPI at the PBCB-MWNTs-DHP film, perhaps through the hydrogen bonding and π–π interactions between EPI and the PBCBMWNTs-DHP. Moreover, the PBCB-MWNTs-DHP film has plenty of nanopores that allow the free entry of substrates and thus results in an increased accumulation of the molecule onto the surface of the electrode. The limit of detection obtained by this method is 10 nM, which is almost 10-fold higher than the Prasad [132] and Goyal [134] electrodes. The method was employed for the analysis of EPI in injection samples.

Alternatively, a MWCNT-bearing terminal monomeric unit may be applied [132] to fabricate an EPI-imprinted polymer-based electrochemical sensor. The procedure for the fabrication of the sensor is described in Fig. 8β. As shown in the figure, EPI (the template) was dispersed into CNT-mer (a monomer). This dispersion was then polymerized at 60 ºC in presence of Ethylene glycol dimethylacryalte, cupric chloride and 2, 2’-bipyridyl, chloroform and triethylamine, to form an EPI-imprinted polymer. After polymerization, the obtained MIP-adduct formed was completely freed from template molecules by treating with a mixture of NaOH and NaH_2_PO_4_. A vacant site was thus created. This molecularly imprinted polymer (MIP) is selective for EPI. The LOD obtained was 0.1 nM. AA, UA, DA did not interfere with the analysis of EPI. However, the synthesis of the MIP is an extremely time consuming process and thus will not be feasible for industrial usage where bulk production of electrodes is needed.

#### Norepinephrine

Norepinephrine (nor-EPI) is an important catecholamine neurotransmitter in the mammalian central nervous system. Nor-EPI is secreted and released by the adrenal glands and the noradrenergic neurons during synaptic transmission. It can be used for treating myocardial infarction hypertension, bronchial asthma and organic heart disease. Extreme abnormalities of nor-EPI levels may lead to diseases such as ganglia neuroblastoma, ganglion neuronal, paraganglioma, Parkinson’ disease. Nor-EPI is a drug belonging to the stimulants that are on the World Anti-Doping Agency's 2005 Prohibited List. It is also critical in mental disease, heart failure and diabetes. Therefore, it is very essential to develop fast, accurate and sensitive methods for its direct determination. The electrochemistry of nor-EPI was studied [136] with an electrode modified with carbon-coated nickel magnetic nanoparticles. Carbon-coated nickel nanoparticles have a large specific surface area, which makes them better in absorbing nor-EPI. The modified electrode reduces the oxidation potential and improves the electrochemical reaction rate. The LOD obtained by the present method was 60 nM. EPI and dopamine interfere in the analysis of nor-EPI. Hence, this method will not be useful if analysis of nor-EPI has to be carried out in real time, since EPI and dopamine always coexist with nor-EPI in biological fluid samples.

Simultaneous determination of nor-EPI, UA, and AA was demonstrated [137] by using a screen printed carbon electrode modified with poly (acrylic acid)-coated multi-walled carbon nanotubes (PAA-MWCNT-SPCE). The significantly higher electrocatalytic activities of PAA-MWCNTs/SPCE for nor-EPI and UA was due to the netlike structure, which provided a larger surface area as well as the affinity adsorption of nor-EPI and UA onto PAA via ion exchange and hydrogen bonding mechanisms, respectively. This led to faster electron transfer kinetics for the oxidation process. The LOD obtained for nor-EPI was 0.13 μM, which indicates that the method is not very sensitive.

Electrochemical determination of nor-EPI on a poly-glycine (pGly) membrane containing silver nanoparticles also was carried out.[138] The peak currents increased nearly five times compared to that of the pGly/GCE. This was ascribed to the large area of the three dimensional structure of the composite membrane, good electron transferring and catalytic properties for nor-EPI. Ascorbic acid did not interfere. The LOD is 0.12 μM, which is higher than the usual levels in biological fluids.

In an electrochemical sensor for nor-EPI based on a carbon paste electrode modified with carbon nanotubes (CNT-CPE) and a molybdenum (VI) complex (MC),[81] the MC acted as a mediator that facilitated the electron transfer process of nor-EPI at the electrode surface. The effect of the potential scan rate (ν) on electrochemical properties of the MC-CNT-PE was studied by CV. Plots of both the anodic and cathodic peak currents (I_p_) were linearly dependent on ν in the range of 10-1000 mV s^-1^ [Fig. 5α (A)]. This indicates that the redox process of MC at the modified electrode was adsorption-controlled. The apparent charge transfer rate constant, k_s_, and the charge transfer coefficient, α, of a surface-confined redox couple was evaluated from CV experiments by using the variation of anodic and cathodic peak potentials with the logarithm of the scan rate and according to the procedure of Laviron [139]. Fig. 5α (B). The results indicated that E_p_ values were proportional to the logarithm of scan rate for ν values. The slopes of the plots in Fig. 5α (C) were used to extract the kinetic parameters α_c_ and α_a_ (cathodic and anodic transfer coefficients, respectively). The slope of the linear segments were equal to -2.303 RT/αnF and 2.303 RT/(1-α) nF for the cathodic and anodic peaks, respectively. The value for α is 0.5.

The following equation was employed to determine the electron transfer rate constant at the modified electrode:

$$ \log {k}_s=\alpha \log \left(1-\alpha \right)+\left(1-\alpha \right) \log \alpha - \log \left( RT/ nF\nu \right)-\alpha \left(1-\alpha \right) nF\varDelta {E}_p/2.3 RT $$

where (1 - α) n_a_ = 0.5, ν is the sweep rate and all other symbols having their conventional meanings. The value of k_s_ was evaluated to be 24.6 s^-1^ by using the above equation. The LOD obtained by this method was 45 nM, which makes this electrode less sensitive than the electrode reported by Sanghavi [66] which has an LOD of 0.354 nM. The authors employed this method for simultaneous determination of nor-EPI, acetaminophen and folic acid. However, ascorbic acid, dopamine and EPI interfere.

#### Serotonin

Serotonin (5-hydroxytryptamine; SER) is a monoamine neurotransmitter synthesized in serotonergic neurons in the central nervous system and plays a crucial role in the emotional system together with other monoamine transmitters such as regulation of mood, sleep, emesis (vomiting), sexuality and appetite. Low levels of SER have been associated with several disorders, notably depression, migraine, bipolar disorder and anxiety. In addition, neurodegeneration of SER containing neurons contributes to late-onset neurological diseases, including Parkinson’s and Alzheimer’s diseases, and possibly to normal ageing of the brain.

A range of nanomaterial-based electroanalytical techniques are reported in the literature for SER. A first example [130] includes an electrochemical sensor based on MWCNTs-ionic liquid (IL) composite for simultaneous determination of SER and dopamine. The LOD for SER is very low (8 nM). The method was employed for the simultaneous determination of both analytes in serum samples. However, UA causes interferences at levels in excess of 10-fold, which is a serious limitation since UA is generally present at high concentrations in biological fluids. In another version,[140] the SER-electrode employs electrochemically reduced graphene oxide in a porphyrine-modified GCE. The LOD for SER is as low as 4.9 nM. Even though this method is sensitive, authors have not carried out analysis of SER in real samples and it remains to be seen whether this method will be applicable to clinical samples.

A GCE modified with MWCNT/chitosan (MWCNTs-CHT/GCE) was used for voltammetric determination of SER [141]. The CHT-CNT composite improved the stability of CNTs in aqueous CHT solution. SER was determined simultaneously with L-dopa at lower oxidation potentials as compared to bare electrode. The LOD obtained for SER was 80 nM. The peak response for the electrode, dropped by 7.5 % for SER after 36 h of fabrication, which indicates limited long-term stability.

Nafion/Ni(OH)_2_ nanoparticles and MWCNTs modified GCE was employed [142] for simultaneous determination of SER and dopamine in the presence of ascorbic acid and uric acid. At pH 7.0 both are present in their anionic form, but dopamine and SER are cationic. Nafion on the GCE surface attracts cationic species and allows them to penetrate to the electrode surface. In contrast, anionic species are repelled and cannot reach the electrode. Owing to the permeation selectivity of the Nafion film, AA and UA cannot undergo electron transfer. The LOD obtained by this method is 3 nM, which is much lower than in the author’s pervious work [141]. However, no interference studies were carried out for EPI and nor-EPI. In related work [143], a carbon ionic liquid electrode was prepared as an electrochemical sensor for simultaneous determination of L-dopa and SER. The preparation of the electrode is schematically shown in Fig. 9γ. The carbon ionic liquid electrode (CILE) was prepared by mixing graphite powder and the ion liquid thoroughly in a mortar to form a carbon paste. A portion of the carbon paste was filled into one end of a glass tube and a copper wire was inserted through the opposite end to establish an electrical contact. After smoothening its surface, the CILE was used as the basic solid electrode. A stock solution of MWCNTs and Co (OH)_2_ in DMF was prepared and pipetted onto the CILE surface with a micro syringe and dried at room temperature. Finally, the electrode surface was coated with Nafion. During these procedures a small bottle was fitted tightly over the electrode so that the solvent could evaporate slowly and a uniform film was formed.

Neurotransmitters have been studied widely with nanomaterial-based sensors because of their efficient electrochemistry and biological significance. Direct electrochemistry will continue to be the powerful strategy for the determination of these neurotransmitters. While sensing neurotransmitters, there is a tradeoff between sensitivity, size of electrode, and speed. The Cu-biomimetic method shows great sensitivity, but because H_2_O_2_ has been employed for catalyzing the reaction, this method is harmful if it has to be used directly in vivo. Overall, a nano-Au/DNA/nano-Au and safranine T modified GCE has provided the highest sensitivity for the determination of dopamine. Recent methods make use of differential pulse voltammetry to discriminate ascorbic acid, uric acid, and dopamine, but they are relatively slow. Future studies should focus on faster electrochemical techniques based on direct electron transfer reactions without using mediators. These methods shall also allow real time measurements in vivo.

### ATC code N05: psycholeptics

A psycholeptic is a medication that produces a calming effect upon the patient. Psycholeptics are classified under N05 in the ATCC. Significant details and figures of merit for code N05 drugs along with references are given in Table S8 in the Electronic Supporting Material.

#### Buspirone

Buspirone (BUS) is an anxiolytic psychoactive drug of the azapirone chemical class. It is primarily used to treat generalized anxiety disorder (GAD). It exhibits an anxiolytic effect similar to that of diazepam, without sedative, muscle relaxing or anticonvulsant properties. The most commonly reported symptom is epileptic seizures; other common symptoms include heart rhythm abnormalities, increased blood pressure, agitation, and hallucinations. It has dopaminergic, noradrenergic, and serotonin-modulating properties and its anxiolytic effects appear to be related to its action on serotonin neurotransmission.

A DNA-templated AgNP-based sensor for BUS was constructed [144] where the nanoparticles were deposited on the surface of a glassy carbon electrode by an electrochemical method in presence of DNA as a scaffold. The presence of DNA made the scaffold to arrange and order the nano-structures, which inhibited aggregation of silver nanoparticles and provided a better catalytic ability. Better sensitivity for detection of BUS was obtained as a result, with a linear range of 2.0 – 70.0 nM and a detection limit of 1.1 nM. A polarographic method was reported [145] for analysis of BUS, which has an LOD of 0.52 nM, about two fold lower than the other electrode [144]. However, mercury is used here as an electrode material which is highly toxic and does not warrant good selectivity. The sensor is not interfered by ascorbic acid, acetaminophen, uric acid and ranitidine. BUS is a probe drug that is often administered along with fexofenadine and omeprazole. There is one HPLC method to detect them simultaneously [146]. A voltammetric method for simultaneous determination of BUS, fexofenadine and omneprazone is needed since, if selective, it will have the advantage of no need for prior sample preparation steps.

#### Chlorpromazine

Chlorpromazine (CPZ) is a phenothiazine drug with an aliphatic side chain, used in the management of psychotic conditions. It controls excitement, agitation and other psychomotor disturbances in schizophrenic patients and reduces the manic phase of manic-depressive conditions. It is used to control hyperkinetic states and aggression and is sometimes given in other psychiatric conditions for the control of anxiety and tension. Thus its determination is important in biological fluids. Among the other electrochemical methods [147], a voltammetric sensor appears to be quite useful for the determination of CPZ. It is based on a GCE that was modified with a multiwalled carbon nanotube-polyethyleneimine (MWCNT-PEI) composite. PEI is electrically conducting and also serves as an agent to disperse the CNTs. The electrode showed a 15.7-fold increase in peak current as compared to a bare GCE. This increase is due to the high conductivity and electroactive surface area of the MWCNT-PEI electrode. Furthermore, simultaneous determination of CPZ was carried out with acetaminophen, uric acid, dopamine and ascorbic acid. The LOD obtained for CPZ by this method is 10 nM. However, authors report RSD from 3.15 % to 15.2 %, which indicates that the method is moderately reproducible.

A CPE was modified with cobalt nanoparticles and then employed for the electrocatalytic determination of chlorpromazine. [148] The use of this CoNP/CPE not only increases the separation of the peaks but also enhances the current response. The anodic and cathodic peak potentials of CPZ shifted toward less negative and less positive values relative to those obtained by a bare CPE. This may be due to faster electron transfer at the surface of the modified electrode. The results also indicate that the electrocatalytic activity of the CoNP/CPE is due to the presence of CoNPs, which not only enhanced the conductivity, surface area, and the electron transfer rate of CPZ at the electrode surface but also facilitated the electron transfer between the biomolecules and the electrode surface. A very low detection limit (0.6 nM) was obtained. This is better than the electrode of ref.[147] in terms of LOD, but a simultaneous determination of CPZ and other molecules generally present along with it in biological fluids was not carried out. The working pH of 4.0 is below the physiological pH, which implies that this CPE cannot be used for routine testing of CPZ in biological fluids.

#### Clozapine

Clozapine (CLO) is an atypical antipsychotic medication used in the treatment of schizophrenia, and is also sometimes used off-label for the treatment of bipolar disorder. Clozapine has numerous severe side effects including agranulocytosis, bowel infarction, and seizures, and has been associated with myocarditis and diabetes though those relationships have not been confirmed. Additionally, it also often causes less serious side effects such as hypersalivation and weight gain. It is an effective antipsychotic drug for treating positive and negative symptoms of schizophrenic patients who do not respond well to traditional neuroleptic drugs.

Electrochemical studies on clozapine have been carried out [149] at CPE modified with TiO_2_ nanoparticles by AdSDPV in solution of pH 9. The presence of the TiO_2_ NPs in the carbon paste increases the available surface area of the electrode and improves its sensitivity by enhancing electron transfer kinetics. The authors extended their work by carrying out simultaneous determination of CLO and thioridazine. Measurement of CLO in the presence of other antipsychotics drugs such as chlordiazepoxide, olanzapine, and quetiapine was also carried out without any reported interference. The LOD is 61 nM. However, ascorbic acid, which is present in large concentrations in biological fluids, interfered if present in 20-fold excess. Thus this method would not be of preferable choice when analysis of CLO needs to be carried out in blood samples, which generally has a large concentration of ascorbic acid.

Shahrokhian’s group worked out a method for electrochemical determination of CLO using a CPE modified with polypyrrole (PPy) that was doped with MWCNTs and New Coccine (NC) using DPV. The peak current for CLO was higher at the PPy/MWCNT/GCE compared to a bare GCE. This improvement in the peak current was said to be due to the increased active surface area of the electrode due to polymerization and strong adsorption of the analyte on the surface of the modified electrode. The oxidation of monomers led to the formation of cation radicals, which repeatedly underwent electropolymerization to form the PPy film. To preserve charge neutrality of the resulting film, some of the counter anions (anionic dopants) present in the electrolyte solution were incorporated into the growing polymer film. NC, with an aromatic structure and effective π-π interactions with the PPy polymer, and also the MWCNT backbone serve as an anionic dopant in the electrodeposition process. This improves polymer loading on the surface of electrode and leads to the formation of a stable and adhesive polymer film. The limit of detection obtained by this method was 3 nM, more sensitive than th at of ref.[149]. Authors also tested the effects of excipients that often accompany CLO in pharmaceutical preparations. Under optimal experimental conditions, a 100-fold molar excess of azathioprine, citalopram hydrobromide, tramadol hydrochloride, methimazole, penicillamine, risperidone, levamisole hydrochloride and chloramphenicol did not disturb the voltammetric signal of CLO, indicating that the method is fairly selective.

#### Risperidone

Risperidone (RISP) is a potent antipsychotic drug that is mainly used to treat schizophrenia (including adolescent schizophrenia), schizoaffective disorder, the mixed and manic states associated with bipolar disorder, and irritability in people with autism. Side effects of risperidone can include significant weight gain and metabolic problems such as diabetes mellitus, as well as tardive dyskinesia and neuroleptic malignant syndrome. Risperidone and other antipsychotics also increase the risk of death in patients with dementia. Thus its analysis is of tremendous importance. Among the electrochemical methods, a CPE modified with MWCNT was presented [150] and used for determination of RISP. This method was based on adsorptive accumulation of RISP onto the paste electrode, followed by reduction of the accumulated species in a voltammetric scan. The LOD is 12 nM, which is not as low as that of the method described below [151]. Moreover, no interference studies have been carried out, which – at present – limits the method to simple matrices such as drugs or spiked serum.

A composite electrode for the determination of RISP was presented [151] that is based on a CPE coated with MWCNTs and the ionic liquid *n*-octylpyridinum hexafluorophosphate and on AdSDPV in pH 4 solution. This electrode shows very attractive electrochemical performance in terms of the electrooxidation of RISP due to the synergistic effects of ionic liquid and MWCNTs. The LOD is 6.54 nM, and the drugs lamotrigine, memantine hydrochloride, rivastigmine tartrate, atenolol, ibuprofen, enalapril maleate, citalopram hydrobromide, and chlordiazpoxide did not interfere. The reduction potentials for RISP reported in refs. [150] and [151] are -810 V and -230 mV respectively. This vast difference in the reduction potentials is probably due to the use of an ionic liquid as a binder, which decreases the resistance of the electrode.

#### Thioridazine

Thioridazine (TIR; also referred to as Mellaril, Novoridazine, Thioril) is a piperidine type of antipsychotic drug belonging to the phenothiazine drug group and was previously widely used in the treatment of schizophrenia and psychosis. Due to concerns about cardiotoxicity and retinopathy at high doses this drug is not commonly prescribed, reserved for patients who have failed to respond to, or have contraindications for, more widely used antipsychotics. A serious side effect is the potentially fatal neuroleptic malignant syndrome. It exerts its actions through a central adrenergic-blocking, a dopamine-blocking and minor anticholinergic activity.

TIR was deteremined [152] with an electrode modified with MWCNT and CoNPs by DPV in pH 7 phosphate buffer. The enhancement in the microscopic area of the electrode by the nanoparticles together with the catalytic effect of the composite modifier resulted in a 55-fold increase of the peak current and a negatively shifted oxidation potential. The LOD is 80 nM, but no interference studies were carried out. Hence, the selectivity cannot be judged.

Electrochemical studies and selective detection of TIR using a CPE modified with ZnS nanoparticles was carried out [153]. The presence of ZnS nanoparticles in the carbon paste increases the available surface area of the electrode and improves sensitivity by enhancing peak currents. In the ZnS-modified CPE, the oxidation peak current is enhanced by a factor of 1.7 (compared to a bare CPE), indicating that ZnS nanoparticles exert good catalytic activity towards the oxidation of TIR. Even though authors obtained only a 1.7-fold enhancement in peak current as compared to the method reported in ref.[152], the LOD obtained is lower (65 nM). The method was then extended to the simultaneous determination of thioridazine and olanzapine. Only a 32-fold excess of ascorbic acid did not interfere with the analysis, which indicates that the method is not fairly selective at higher concentrations of AA.

### ATC code N06: psychoanaleptics

A psychoanaleptic is a medication that produces an arousing effect. Antidepressants, psychostimulants, nootropics, and anti-dementia drugs are all psychoanaleptics. Significant details and figures of merit for code N06 drugs along with references are given in Table S9 in the ESM.

#### Caffeine

Caffeine (CAF; 1,3,7-trimethyl-purine-2,6-dione) is an alkaloid found in many food products such as coffee, tea, yerbamate, guarana berries, cola nuts and cacao beans. It promotes many physiological functions such as gastric acid secretion, diuresis, and stimulation of central nervous system. Within other formulations, it is used for the treatment of migraine and for anti-inflammatory and diuretic action. However, at higher concentration it is toxic and causes cardiovascular disease, depression and hyperactivity. Numerous electrochemical methods for its determination are known. A remarkable sensitive electrode employs a boron-doped diamond (BBD) electrode for voltammetric determination of CAF employing DPV in pH 4.5 buffer [154]. The LOD is 35 nM. Simultaneous determination of CAF and paracetamol in pharmaceutical formulations also was demonstrated, but the possible interference by ascorbic acid and uric acid, which commonly interfere in the analysis of caffeine and paracetamol in biological fluids, was not studied.

Gupta’s group [155] fabricated a GCE modified with multiwall carbon nanotubes as a voltammetric sensor for the simultaneous determination of ascorbic acid and CAF. The method was employed to the analysis of CAF in tea leaves, coffee, and cold drinks. The detection limit is 3.52 nM, which indicates that the method is very sensitive. CAF also was determined [156] by SWV at an edge-plane pyrolytic graphite electrode (EPPGE) employing pH 7.2 buffer. Electron transfer rate constants for a large variety of redox couples at edge plane pyrolytic graphite electrodes have been found to be 1000-times faster than on conventional electrodes. The method was further extended to the simultaneous determination of ascorbic acid and CAF. The detection limit is 2.6 nM, and the linear working range (from 0.02 to 100 μM) is remarkably wide. The method was employed to the analysis of CAF in human urine, pharmaceutical formulations and coffee samples. The authors observed other peaks along with that for caffeine, however, no attempts were made to identify them. It was speculated that they may be attributed to the oxidation of common urinary compounds such as xanthine, uric acid, or ascorbic acid. In any case, these methods of refs. [155] and [156] are among the electrochemical methods having the highest sensitivities for CAF.

#### Clomipramine

Clomipramine (CLOM) has been used experimentally to reduce relapses in cocaine addicts, and to repair neurotransmitter damage caused by cocaine. It being a very important tricyclic antidepressant drug, CLOM is widely used in the form of its hydrochloride to treat anxiety and obsessive-compulsive disorder. Electroanalytical methods for CLOM were described [157] that rely on a GCE that was modified with poly (aminobenzene sulfonic acid) and Pt nano-clusters. CLOM exhibited a sensitive electrochemical response in pH 8.1 phosphate buffer. The Pt nanoparticles provided a 3-D and conductive structure for the polymer immobilization while the polymer film contains electron-rich N atoms. The negatively charged sulfo group attracted the CLOM cation, and this resulted in an LOD as low as 1 nM. The method was employed to the analysis of CLOM in pharmaceutical formulations. Fujishima’s group [158] demonstrated the practical utility of diamond electrodes for the amperometric detection of CLOM in combination with flow injection analysis and HPLC, respectively. The LOD was 0.5 nM, which makes this electrode more sensitive than the one reported in [157]. However, no interference studies were performed and thus selectivity of the method cannot be commented upon.

#### Desipramine, imipramine and trimipramine

The tricyclic antidepressants imipramine (IMI), trimipramine (TRI) and desipramine (DES) are active ingredients of psychiatric drugs widely used in the treatment of depressive disorders, such as major depressive disorder, dysthymia, and bipolar disorder, especially of the treatment-resistant variants. However, their overdose is fatal to the central nervous system and may result in drowsiness, convulsions, respiratory disorders, ophthalmoplegia and, finally, coma. Hence, their determination in pharmaceutical formulations, urine and blood serum is of tremendous importance.

Among the electrochemical methods, a GCPE was reported that was modified with an Amberlite (XAD2) and titanium dioxide nanoparticles (TNPs) and used for the determination of IMI, TRI and DES by Sanghavi and Srivastava [83]. In order to understand the whole electrochemical mechanism involved in the reaction of IMI, the voltammetric process was recorded in two repetitive cycles. In the first cycle, only one irreversible peak (O1) was obtained at +0.878 V, which was attributed to the electro-oxidation of IMI. The electrochemical oxidation of these three drugs occurs at the nitrogen atom in the cyclohexane ring, which resulted in the formation of a radical cation. This is comparable to the oxidation of methyliminobibenzyl. On the reverse sweep from +1.2 to -0.4 V, a reduction peak (R2) at 0.104 V was observed due to the formation of a dimer [Fig. 5γ], which gets subsequently oxidized (O2) in the second cycle at 0.163 V.

The appearance of the oxidation peak (O1) is due to a 2-electron and 1-proton irreversible oxidation of the radicals. The R2/O2 redox peak is caused by the reversible 1-electron and 1-proton oxidation and reduction of the dimer. The reactions are depicted in Table S2 in the Electronic Supporting Material. XAD2, a nonionic resin, promotes accumulation of the three drugs onto the electrode surface and to the titanium dioxide NPs due to their high surface area/volume ratio, which further enhances the peak current during CV. Thus, the synergistic effect of both additives increases the sensitivity for IMI, TRI and DES. The LODs obtained for IMI, TRI and DES are 0.393, 0.351 and 0.453 nM respectively. Ascorbic acid and uric acid did not interfere, which makes the method quite selective.

A boron-doped diamond electrode (BDDE) was applied [158] to the determination of IMI and DES. The LOD obtained was 3 nM, which is higher than that of the Sanghavi and Srivastava method [83]. Moreover, the BDDE was coupled to HPLC. This renders the method much more selective but this also includes a substantial additional effort and increases the time for analysis. In order to remain selective in case of complexed and unknown (that is, not just spiked serum samples), HPLC prior to electrochemical detection probably is mandatory. The authors did not carry out interference studies with respect to the response of the BDDE to cysteine, ascorbic acid, and uric acid.

#### Trazodone

Trazodone, (TRZ) is a weak inhibitor of monoamine reuptake. Its major mechanism of action seems to be the antagonism at certain serotonin receptors. TRZ is used for the treatment of major depression, sometimes in conjunction with selective serotonin reuptake inhibitors like fluoxetine. The main side effects associated with TRZ administration include nausea, insomnia, agitation, dry mouth, constipation, headache, hypotension, blurred vision and confusion.

Among the electrochemical methods based on the use of carbon nanomaterials, a GCE modified with multi-walled carbon nanotubes was employed [159] in the DPV mode for the determination of TRZ. The LOD was 24 nM. Ascorbic acid interfered with the voltammetric analysis of TRZ in pharmaceuticals and biological fluids containing high amounts of ascorbic acid, indicating that the method is not quite selective in nature. Voltammetric analysis of TRZ also was carried out [160] by employing DPV in pH 10 B.R. buffer. The LOD was 2.45 μM, which makes the method less sensitive as compared to the previous one. Also, metallic mercury is quite toxic and thus a sensor using it as an electrode material is not viable in terms of real-time sensing of blood. Moreover, authors did not investigate the selectivity of their electrode.

#### Venlafaxine and desvenlafaxine

Venlafaxine (VLF) is an arylalkanolamine serotonin–norepinephrine reuptake inhibitor and primarily used for the treatment of major depression in adults. Desvenlafaxine (DVF), also known as O-desmethyl-VLF, is another antidepressant and the synthetic form of the major active metabolite of VLF. It is also being targeted as the first non-hormonal based treatment for menopause. However, an overdose of VLF or DVF results in symptoms such as depression, serotonin toxicity, seizure, or cardiac conduction abnormalities. Hence, their determination is of tremendous importance.

Adsorptive stripping DPV was reported [143] to be a useful approach for detecting VLF and DVF individually by employing a GCE modified with a nafion–carbon nanotube composite. VLF has a pK_a_ value of 9.4. When pH 7.0 buffer is employed as the supporting electrolyte, VLF is present in cationic form. This is also the case for DVF in pH 5.0 buffer. Positively charged VLF and DVF can exchange protons of nafion, which is cationic polymer, and this causes their accumulation on the electrode surface. The LODs for VLF and DVF are 12.8 and 21.1 nM, respectively. Ascorbic acid and uric acid do not interfere. Both a hanging mercury dropping electrode [161] and a mercury film electrode [162] were also applied to the determination of VLF but they are not as sensitive as the one of ref. [163]. Moreover, the use of mercury as an electrode material makes the method less attractive. It is interesting to note that there is no other electrochemical method available for voltammetric determination of DVF.

While preparing this review article, we also noted that except for caffeine, other drugs of code N06 have not been studied vastly by voltammetry. In fact, a recent review mainly covers electrochemical methods for caffeine determination [164]. While caffeine is most important and thus deserves exhaustive studies, we believe that novel sensors for drugs such as tricyclic antidepressants, venlafaxine, and desvenlafaxine are urgently needed.

### ATC code N07: other nervous system drugs

Nervous system drugs not categorized under ATC codes N01-N06 fall into a special category referred to as ATC code N07 drugs. Figures of merit of assays for code N07 drugs along with references are given in Table S10 in the Electronic Supporting Material and will be discussed in more detail in the following.

#### Cinnarizine

Cinnarizine [CNR; 1-(diphenylmethyl)-4-(3-phenyl-2-propenyl) piperazine] is a piperazine derivative that blocks the histamine H_1_-receptor and calcium channels. It improves the cerebral blood flow and is widely used orally for the treatment of cerebral apoplexy, cerebral arteriosclerosis and post-traumatic cerebral symptoms. It is also used for the control of nausea and vomiting. CNR is highly effective against motion sickness and, in contrast to other drugs, has fewer side effects. It is also used in the treatment of cerebral and peripheral vascular disorders.

Electrochemical determination of CNR was carried out [165] on an unmodified GCE. An LOD of 9 nM is reported. Even though this method is sensitive, the authors have not studied the effect of ascorbic acid and uric acid. Others have described [166] the voltammetric determination of CNR using a GCE modified with MWCNTs. The LOD is 2.6 nM, lower than the electrode described in ref.[165]. The response to CNR was investigated at pH 2.5, which is way below the physiological pH. Thus, these methods cannot be employed for analysis of CNR in-vivo.

#### Dextromethorphan

Dextromethorphan [DEX; (+)-3-methoxy-17-methyl-(9α, 13α, 14α)-morphinan) is an over-the-counter cough suppressant commonly found in cold medications. It is a safe and orally administered antitussive that has a central action at the cough center in the medulla. It is an innocuous non-narcotic cough suppressant and mainly used for the relief of nonproductive cough and the treatment of respiratory disorder. Other medical uses of DEX include the temporary relief of sinus congestion, runny nose, cough, sneezing, itching of the nose and throat, and watery eyes caused by hay fever, allergies, cold, or influenza.

Electro-oxidation of DEX on a GCE modified with carbon nanotubes and an ionic liquid was studied [167] using amperometry and a pH 7.4 phosphate buffer. The LOD is 8.81 μM, which limits the method to the determination of DEX in pharmaceutical formulations. The working potential is +0.9 V so that ascorbic acid, uric acid and dopamine will interfere if present.

#### Naltrexone

Naltrexone (NTX), a cyclopropyl derivative of oxymorphone opiate, is a potent, long acting and orally effective opiate antagonist with few side effects. It is used for the treatment of opioid and alcohol dependence. The blockade of opioid receptors is the basis behind its action in the management of opioid dependence. The mechanism of action in alcohol dependence is not fully understood, but, as an opioid-receptor antagonist, it is likely to be due to the modulation of the dopaminergic mesolimbic pathway, which ethanol is believed to activate.

Among the recent nanomaterial-based methods, the work performed by the Shahrokhian group [168] is most significant. They carried out an electrochemical study on NTX on the surface of a GCE modified with Nafion-doped carbon nanoparticles using DPV. The pK_a_ value of NTX is 8.20. Therefore, it is positively charged at this pH and can exchange with the protons of the nafion layer. As a result, the concentration of NTX near the electrode surface increases, which in turn increases the LOD. The carbon nanoparticles, due to their large surface area and high electrical conductivity, also causes an increase in the peak current. The LOD is 0.1 μM, which still is not very good. Ascorbic acid and uric acid are likely to interfere since both ascorbic (pK_a_ = 4.17) and uric acid (pK_a_ = 5.75) will exchange with H^+^ of nafion at pH 3. Thus this method is not sensitive and selective in nature.

In another work by this group [169], a GCE modified with a bilayer of MWCNT and polypyrrole doped with nitrazine yellow was used for voltammetric determination of NTX at pH 6. Nitrazine yellow (NY), an anion, was doped into polypyrrole during the electropolymerization step. The pH transition range of NY (a pH indicator), is from 6.0 to 7.0, with a sharp neutral point at pH 6.6. Therefore, NY exists in the azo form in solution of pH 6.0. The pK_a_ values for NTX are 8.20 and 9.63. The first corresponds to the dissociation of the protonated aliphatic nitrogen proton, and the second to the dissociation of the phenolic proton. Therefore, NTX exists as a cation at pH 6.0 and this is the basis for the electrostatic interaction between NTX (with its positive charge) and NY (with two negative charges). The LOD is 12 nM, which is much lower than that of the first method. Interferences from both ascorbic as well as uric acid are less likely, because they are present as anions at pH 6.0. Unlike the previous method [168] by Shahrokhian’s group, this method [169] is both sensitive and selective in nature for determination of NTX.

#### Nicotine

Nicotine, [NIC; 3-(1-methyl-2-pyrrolidinyl) pyridine], is the main alkaloid in tobacco leaves. Despite its high toxicity, recent studies suggested its therapeutic opportunities in some neurodegenerative diseases like Alzheimers and Parkinson’s NIC is the primary neuroactive alkaloid in tobacco and has been the focus of a great deal of research in recent decades that led to the discovery of an increasing number of harmful effects on human health. The appeal of electrochemical sensing/detection methods lies primarily in their low instrumental costs, relatively simple operation, and rapid detection times. NIC gives a distinct signal [170] at a pencil graphite electrode in the presence of the anionic surfactant, sodium dodecyl sulfate (SDS). The sensitivity of the surfactant-modified electrode was about 1.6 times better than that of bare electrode. In addition, the peak potential shifted to less positive values by about 80 mV. This indicated that the oxidation of NIC becomes easier in presence of a micellar system. NIC is positively charged (monoprotonated) at pH 7.0. The adsorption of anionic SDS micelles onto the surface of the electrode generates a negatively charged hydrophilic film oriented towards the water bulk phase. Thus, positively charged nicotine can accumulate in the negatively charged crown of anionic SDS micelles. This decreases the overpotential of the electrode and increases the electron transfer rate. The LOD is 2 μM, which makes this sensor not really sensitive. The electrochemical detection of NIC on a basal plane pyrolytic graphite electrode modified with layers of multi-walled carbon nanotubes was carried out [171] using AdSSWV by employing pH 7.4 buffer. The LOD is 1.5 μM which is slightly better than that of ref.[170].

## Future Challenges and Outlook

This review on nanomaterial-based electrochemical strategies for the detection of neurological drugs has thus far focused chiefly on enabling issues of surface chemistry and materials science that enhance the detection sensitivity, selectivity and versatility. In addition to this materials-oriented research, the following are some of the emerging trends.

### Multi-target detection systems

**Fig. 10:**
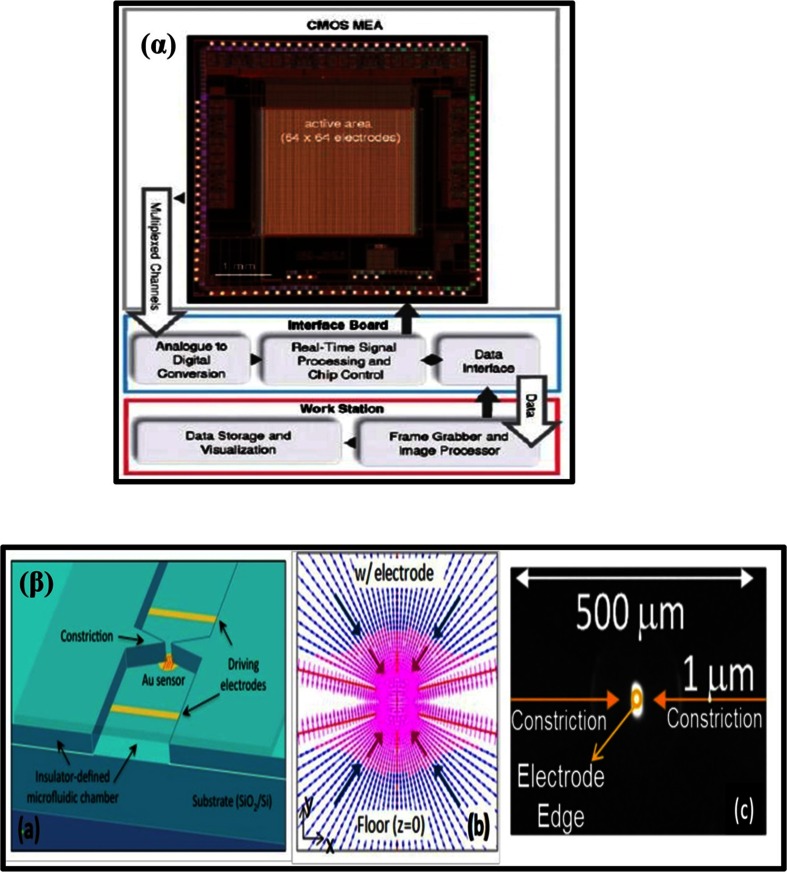
(α) An example multiplexed CMOS microelectrode array device with on-chip sensing and signal processing [174]. (β) (a) Device design for coupling dielectrophoresis with immobilized capture probes for electrochemical sensing of target analytes; (b) Coupling long-range electrothermal flow (blue) with localized dielectrophoretic trapping to enhance target diffusion lengths; (c) Directed transport of fluorescently labeled target species onto the sensor surface [177]

Electrochemical detection strategies have traditionally been composed of single working electrodes, which can be applied to individually detect several target species at may be detected one-by-one at their characteristic redox potentials. This strategy is limited by a lack of means for the simultaneous detection of multiple target species and with the spatial and temporal resolution that is necessary for monitoring neurological processes [172]. An emerging trend for this purpose is the application of multiplexed microelectrode array approaches [173] as shown in Fig. 10α [174]. Typically, these are devices with addressable metal electrode arrays connected by CMOS (Complementary Metal Oxide Semiconductor) switches, with an overlayer of electropolymerized conducting polymer to extend the potential range for electrode stability. Each electrode within the array is functionalized with a particular enzyme to enable specific detection of each neurotransmitter of interest through oxidation [175, 176]. For instance, neurotransmitters such as dopamine, norepinephrine, epinephrine, or serotonin can be specifically oxidized by the functionalized enzymes at the particular electrode in the array to produce the electroactive by-product of hydrogen peroxide due to interaction of glutamate, acetylcholine, choline, glucose, etc., with their respective oxidase. In this manner, these neurotransmitters can be simultaneously monitored in real-time.

### Micro/nanofluidics for analyte pre-concentration

Within most biosensor paradigms, gains to detection sensitivity are largely limited by slow mass transport of target analytes towards the sensing surface. This causes poor binding kinetics and delayed signal onset. Such limitations can be alleviated by microfluidic device designs that promote 3D diffusion conditions and electrokinetic means to direct the mass transport of target analytes. This was accomplished by dielectrophoresis [178] and by isotachophoresis [179]. In Fig. 10β (a), we show an example where sharp insulator constrictions within a microfluidic channel can cause the frequency-selective dielectrophoretic trapping of polarized target analytes under an applied AC field, due to sharp bending of field lines [180]. In this manner, long-range electrothermal [177] and electro-osmotic flow [181] can be combined with localized frequency-selective dielectrophoretic trapping [see Fig. 10β (b)] to direct target biomarkers towards the sensor surface [(Fig. 10β (c)] for improving sensitivity.” Another emerging trend consists in signal amplification through redox cycling within nanocavities due to near-instantaneous diffusion across the points of generation and collection of redox species within a nanofluidic device [182, 183]. On a related note, electrochemical detection within micro/nanofluidic systems will also enable high-sensitivity measurement within small sample volumes for detection, which is especially relevant for measurements at high spatial and temporal resolution, using droplets collected by microdialysis [184–186].

### Microelectrode detection systems for real-time in-vivo monitoring or in-vitro within cell cultures

The emergence of facile procedures to fabricate and integrate microelectrodes within nanostructured fluidic cells has opened several new frontiers for electrochemical analysis. First, the traditional 2D diffusional transport on electrode surfaces is extended into the third dimension by sharp-features on microelectrodes, thereby enabling improved target transport to the sensing surface. Secondly, the lowered double-layer capacitance of microelectrodes causes extremely fast charging times that depend on the RC time constant. Hence, biochemical events can be observed over sub-microsecond time scales using methods such as fast-scan cyclic voltammetry [187, 188]. Current studies of interest include measurement of cell communication through following secretions that occur through membrane exocytosis. In this manner, secreted species including catecholamines (dopamine, epinephrine, and norepinephrine), serotonin and histamine can be electrochemically monitored in real-time either in-vivo or in-vitro within cell cultures [189]. Monitoring of neurotransmitters across the synapse is another area of interest [190]. Here, sub-micron scale spatial resolution and sub-millisecond scale temporal resolution is necessary, and was accomplished by fast-scan cyclic voltammetry on carbon fiber microelectrodes. These emerging advances promise further sensitivity and selectivity gains, as well as highly integrated operation through active interfacing with biosystems [191].

### Aptamer based electrochemical biosensors

Aptamers are single stranded nucleic acid-based molecules (DNA or RNA), which are identified by high throughput in vitro selection methods to bind with particular molecules of choice [192, 193]. They have been adapted to an immobilized format without affecting their affinity for the target molecule [194]. In contrast to antibodies, which are produced by in vivo methods, the three-dimensional conformations of aptamers can be tailored via synthetic selection procedures and through modified nucleotides to have well-modulated affinities towards particular targeted molecules [195], thereby enabling competitive assays with well-defined “on” and “off” rates (k_on_ and k_off_). Aptasensors for biomolecules have been developed for different detection platforms, including fluorescence, surface-enhanced Raman spectroscopy (SERS), microgravimetric, and electrochemistry. While the application of aptamers for neurological drugs is at its incipient stage, a number of recent studies reported an electrochemical biosensor using target-specific aptamers [196–198].

**Fig. 11:**
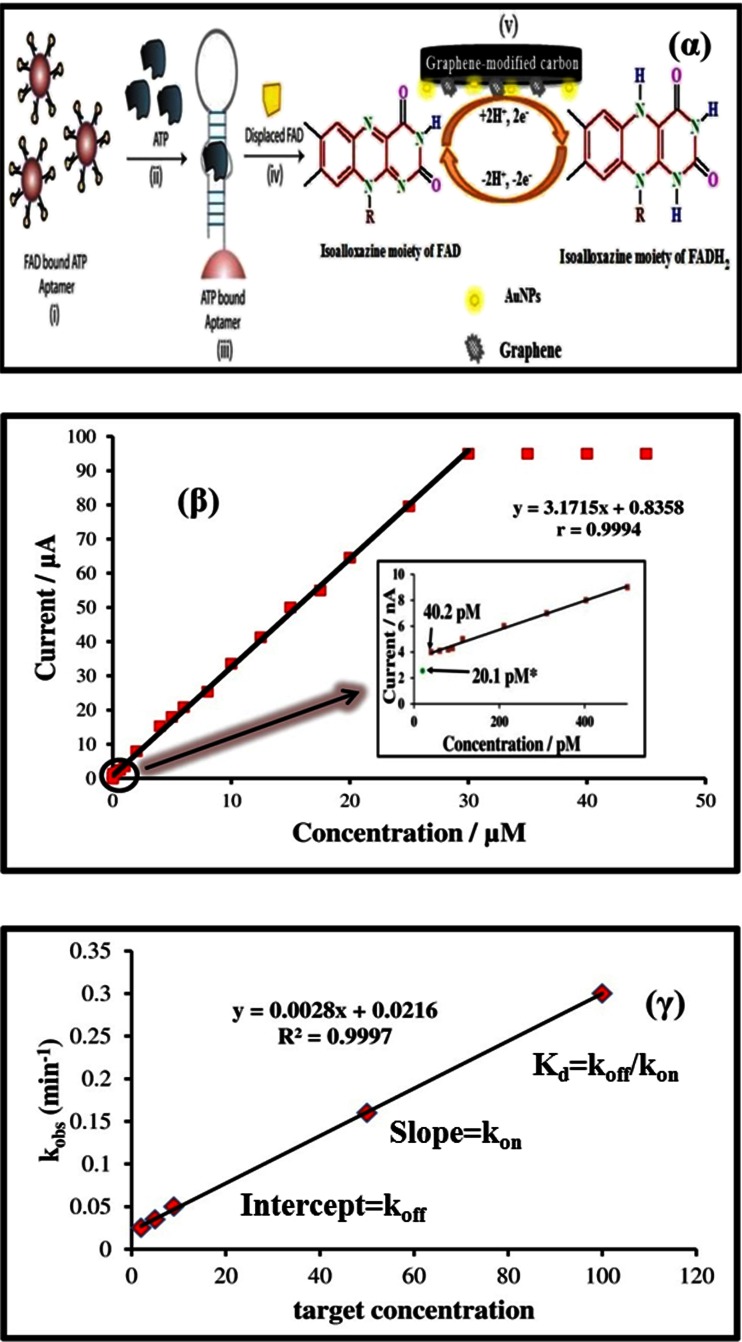
(α) Design of an ATP detection system with FAD displaced signaling via AdS-SWV. (i) FAD as a suboptimal target is bound to the ATP aptamer (ATPA) preconjugated to streptavidin-coated magnetic beads (sphere). (ii) The presence of ATP, which is the native target for ATPA, displaces the prebound FAD (iii). The displaced electroactive FAD (iv) is measured via AdS-SWV [employing a graphene − AuNP − CPE (v)] to generate a measurable signal [23]. (β) Varying the aptamer concentration in the hybridization assay [23]. (γ) Method for calculation of the kinetic (k_on_ and k_off_) and affinity (K_d_) parameters for the hybridization assay

The competitive assay feature of aptamers was recently applied for calculating the (k_on_ and k_off_) rates of the ATP aptamer to ATP using an electrochemical detection platform [23]. In this article, competitive binding of ATP to FAD pre-conjugated aptamer was applied to release FAD for subsequent detection at graphene-modified electrodes (Fig. 11α). A key aspect of this strategy is the application of a so-called “double-surface” technique-a nano-colloid of high surface area immobilized with the capture probe for target recognition and a separate graphene-modified surface for electrochemical detection. On one hand, the ensuing free 3D diffusion conditions and high capture probe concentration at the recognition surface ensure fast target binding kinetics, while on the other hand, the signaling electrode and transduction technique can be separately optimized, through surface modification, microfluidic pre-concentration and/or nanostructuring to enhance detection sensitivity. The concentration of the capture probe can be increased without limitations in terms of steric hindrance from neighboring hybridized capture probes and due to the inherently faster DNA hybridization in solution versus that on the signaling surface (see Fig. 11β). This resulted in a dynamic range that is five log orders wide a rapid hybridization kinetics even at the detection limit and thus were able to determine kinetic (k_on_ and k_off_) and affinity (K_d_) parameters of the assay. Hypothetical Fig. 11γ shows how this real-time monitoring capability can be applied to compute the kinetics (k_on_ and k_off_) and affinity (K_d_) parameters of the assay. The authors also challenged their assay on real samples, viz. blood serum, litchi fruit and banana samples.

The application of similar aptamer binding approaches for the electrochemical detection of neurological drugs and neurotransmitters can enable highly specific assays and far lower detection limits than the current state of the art. If these aptamers are combined with nanomaterial based sensors, the field of electrochemistry for neurological drugs and neurotransmitters (or any other class of drug) will make a substantial leap in the future.

While writing this critical review, we have noticed that only drugs of ATC codes N2, N4 and neurotransmitters have been preferentially studied by researchers in depth. A “*step-relation like*” treatment has been given to codes N1, N3, N5, N6 and N7 classes of ATC code drugs in the field of chemically modified electrodes since not much work has been carried out on these drugs. Literature reveals that paracetamol, caffeine, dopamine etc. have been the most favorite drugs of electrochemists around the world. We hope that after reading this critical review, researchers around the world will be motivated to enter into unknown territories of ‘*not so common*’ drugs. There is plethora of drugs whose electrochemistry is yet to be studied. We also note the preferred use of certain nanomaterials, mainly carbon nanotubes and other carbon species as well as gold nanoparticles (NPs) to prepare chemically modified electrodes, whilst NPs made from silver, platinum, palladium, or metal oxides have not been employed vastly. Employing such NPs as modifiers will predictably form a highly interesting topic for future research in the field of nanomaterial-based electrochemical sensors.

## Electronic supplementary material

Below is the link to the electronic supplementary material.

ESM 1 (PDF 438 kb)

## Acronyms used in this review

AdSV: adsorptive stripping voltammetry
ATC: anatomical therapeutic classification
AA: ascorbic acid
ASA: acetylsalicylic acid (aspirin)
BPPGE: basal plane pyrolytic graphite electrode
BEN: benorilate
BDDE: Boron doped diamond electrode
B. R.: Britton-Robinson
BUS: buspirone
CAF: caffeine
CAP: capsaicin
CBZ: carbamazepine
CD: carbidopa
CFME: Carbon fiber microelectrode
CPE: carbon paste electrode
CNS: Central nervous system
CHT: chitosan
CLZ: chlorpromazine
CNR: Cinnarizine
CLOM: clomipramine
CLO: clozapine
CoNPs: cobalt nanoparticles
COD: codeine
CV: cyclic voltammetry
DES: desipramine
DVF: desvenlafaxine
DEX: dextromethorphan
DPV: differential pulse voltammetry
DA: dopamine
EIS: electrochemical impedance spectroscopy
EPI: epinephirine
EPPGE: edge-plane pyrolytic graphite electrode
FSCV: fast scanning cyclic voltammetry
GBP: gabapentin
GCE: glassy carbon electrode
GCPE: glassy carbon paste electrode
AuNPs: gold nanoparticles
GR: graphene
GNS: graphene nanosheet
HPLC: high performance liquid chromatography
IMI: imipramine
IL: ionic liquid
LMT: lamotrigine
L-Dopa: levodopa
LOD: limit of detection
MOR: morphine
MWCNT: multiwalled carbon nanotube
NAF: nafion
NTX: naltrexone
NP: nanoparticle
NC: new coccine
NIC: nicotine
nor-EPI: norepinephrine
PCT: paracetamol
PCPE: plain carbon paste electrode
PASA: poly (amidosulfonic acid)
PPy: polypyrrole
PRO: procaine
PGE: pyrolytic graphite electrode
RISP: risperidone
SER: serotonin
AgNPs: silver nanoparticles
SWCNT: single walled carbon nanotubes
SDS: sodium dodecyl sulfate
SWV: square-wave voltammetry
SUM: sumatriptan
TIR: thioridazine
TiO_2_ NPs: titanium dioxide nanoparticles
TRA: tramadol
TRZ: trazodone
TRI: trimipramine
UA: uric acid
VF: venlafaxine
VFc: vinylferrocene
ZnO: zinc oxide.
