# Supplementary material for: Nanomaterial-based electrochemical sensing of neurological drugs and neurotransmitters
Source: Mikrochim Acta. 2014 Jul 8;182(1):1–41. doi: 10.1007/s00604-014-1308-4 (PMC4281370; doi:10.1007/s00604-014-1308-4)
Supplement: Supplementary file 1 — (PDF 438 kb) [file 604_2014_1308_MOESM1_ESM.pdf]

Electronic Supporting Material

Nanomaterial-based electrochemical sensing of neurological drugs and neurotransmitters

Bankim J. Sanghavi, Otto S. Wolfbeis, Thomas Hirsch, Nathan S. Swami

Table S1. List of reviews involving various aspects of electrochemical sensors. References are presented as footnotes below the table.

First Author	Year	Title	Ref.
**(a) Carbon based electrodes**			
van der Linden	1980	Glassy carbon as electrode material in electroanalytical chemistry	(1)
Murray	1987	Chemically modified electrodes. Molecular design for electroanalysis	(2)
Kalcher	1990	Chemically modified carbon paste electrodes in voltammetric analysis	(3)
Gorton	1992	Amperometric glucose sensors based on immobilized glucose-oxidizing enzymes and chemically modified electrodes	(4)
O'Neill	1993	Sensor-tissue interactions in neurochemical analysis with carbon paste electrodes in vivo	(5)
Kalcher	1995	Sensors based on carbon paste in electrochemical analysis: a review with particular emphasis on the period 1990-1993	(6)
Wang	1995	Remarkably selective metalized-carbon amperometric biosensors	(7)
Gorton	1995	Carbon paste electrodes modified with enzymes, tissues, and cells	(8)
Gorton	1996	Amperometric biosensors based on carbon paste electrodes chemically modified with redox-enzymes	(9)
Svancara	1999	Testing of unmodified carbon paste electrodes	(10)
Svancara	2000	Physico-chemical processes in analytical electrochemistry with carbon paste electrodes. An overview	(11)
Habermüller	2000	Electron-transfer mechanisms in amperometric biosensors	(12)
Barek	2001	Electrochemical methods for monitoring of environmental carcinogens	(13)
Ferancova	2001	Cyclodextrins as electrode modifiers	(14)
Svancara	2001	Carbon paste electrodes in modern electroanalysis	(15)
Zen	2003	Recent updates of chemically modified electrodes in analytical chemistry	(16)
O'Neill	2005	Long-term monitoring of brain dopamine metabolism in vivo with carbon paste electrodes	(17)
Banks	2005	Electrocatalysis at graphite and carbon nanotube modified electrodes: edge-plane sites and tube ends are the reactive sites	(18)
Banks	2006	New electrodes for old: from carbon nanotubes to edge plane pyrolytic graphite	(19)
Kalcher	2006	Heterogeneous carbon electrochemical sensors	(20)
Radi	2006	Applications of stripping voltammetry at carbon paste and chemically modified carbon paste electrodes to pharmaceutical analysis	(21)
Barek	2007	Nontraditional electrode materials in environmental analysis of biologically active organic compounds	(22)
Kraft	2007	Doped Diamond: A Compact Review on a New, Versatile Electrode Material	(23)

Stozhko	2008	Modified carbon-containing electrodes in stripping voltammetry of metals Part I. Glassy carbon and carbon paste electrodes	(24)
Rodriguez-Mendez	2008	Electronic tongue based on voltammetric electrodes modified with materials showing complementary electroactive properties. Applications	(25)
Barek	2008	Adsorptive stripping voltammetry of environmental carcinogens	(26)
Grieshaber	2008	Electrochemical biosensors - sensor principles and architectures	(27)
Zima	2009	Recent advances in electroanalysis of organic compounds at carbon paste electrodes	(28)
Kalcher	2009	Electrochemical sensors and biosensors based on heterogeneous carbon materials	(29)
Svancara	2009	Carbon paste electrodes in the new millennium	(30)
Svancara	2009	Carbon paste electrodes in facts, numbers, and notes: a review on the occasion of the 50-years jubilee of carbon paste in electrochemistry and electroanalysis	(31)
Vytras	2009	Carbon paste electrodes in electroanalytical chemistry	(32)
Lubert	2010	History of electroanalytical methods	(33)
Opallo	2011	A review on electrodes modified with ionic liquids	(34)
Girousi	2011	The last decade of carbon paste electrodes in DNA electrochemistry	(35)
Jose Luis	2013	Recent advances in graphite powder-based electrodes	(36)
**(b) Nanomaterials in electrochemical sensors**			
Wang	2003	Nanoparticle-based electrochemical DNA detection	(37)
Davis	2003	Chemical and biochemical sensing with modified single walled carbon nanotubes	(38)
Wang	2005	Carbon-nanotube based electrochemical biosensors: a review	(39)
Lin	2005	Carbon nanotubes (CNTs) for the development of electrochemical biosensors	(40)
Banks	2005	Edge plane pyrolytic graphite electrodes in electroanalysis: an overview	(41)
Yanez-Sedeno	2005	Gold nanoparticle-based electrochemical biosensors	(42)
Trojanowicz	2006	Analytical applications of carbon nanotubes: a review	(43)
Wildgoose	2006	Chemically Modified Carbon Nanotubes for Use in Electroanalysis	(44)
Wang	2007	Nanoparticle-based electrochemical bioassays of proteins	(45)
Zayats	2008	Design of amperometric biosensors and biofuel cells by the reconstitution of electrically contacted enzyme electrodes	(46)
de la Escosura-Muniz	2008	Electrochemical analysis with nanoparticle-based biosystems	(47)
Ward	2008	Fabrication and applications of nanoparticle-modified electrodes in stripping analysis	(48)
Pingarron	2008	Gold nanoparticle-based electrochemical biosensors	(49)
Sardar	2009	Gold nanoparticles: Past, present, and future	(50)
Huffman	2009	Carbon-fiber microelectrodes for in vivo applications	(51)
Maehashi	2009	Label-free electrical detection using carbon nanotube-based biosensors	(52)
Qureshi	2009	Review on carbon-derived, solid-state, micro and nano sensors for electrochemical sensing applications	(53)
Zagal	2009	Carbon nanotubes, phthalocyanines and porphyrins: attractive hybrid materials for electrocatalysis and electroanalysis	(54)
Toghill	2010	Metal Nanoparticle Modified Boron Doped Diamond Electrodes for Use in Electroanalysis	(55)
Jacobs	2010	Carbon nanotube based electrochemical sensors for biomolecules	(56)
Henstridge	2010	Voltammetric selectivity conferred by the modification of electrodes	(57)

		using conductive porous layers or films: The oxidation of dopamine on glassy carbon electrodes modified with multiwalled carbon nanotubes	
Mazloum-Ardakani	2011	Carbon nanotubes in electrochemical sensors	(58)
Musameh	2012	Application of Carbon Nanotubes in the Extraction and Electrochemical Detection of Organophosphate Pesticides: A Review	(59)
Pumera	2012	Voltammetry of carbon nanotubes and graphenes: excitement, disappointment, and reality	(60)
Dey	2013	Nanomaterial-based functional scaffolds for amperometric sensing of bioanalytes	(61)
**(c) Voltammetry of drugs**			
Patriarche	1986	New trends on modified electrodes: applications to drug analysis	(62)
Vire	1989	Adsorptive stripping voltammetry applied to drug analysis: a powerful tool	(63)
Kauffmann	1993	Pharmaceutical and biomedical applications of electoanalysis. A critical review	(64)
Hart	1994	Screen-printed voltammetric and amperometric electrochemical sensors for decentralized testing	(65)
Abu Zuhri	1998	Applications of adsorptive stripping voltammetry for the trace analysis of metals, pharmaceuticals, and biomolecules	(66)
Garrido	2004	Electrochemical Analysis of Opiates. An Overview	(67)
Radi	2006	Applications of stripping voltammetry at carbon paste and chemically modified carbon paste electrodes to pharmaceutical analysis	(68)
Ozkan	2007	Electroanalytical application of carbon based electrodes to the pharmaceuticals	(69)
Felix	2010	Fast and accurate analysis of drugs using amperometry associated with flow injection analysis	(70)
Gupta	2011	Voltammetric techniques for the assay of pharmaceuticals. A review	(71)
Gumustas	2011	The role of and the place of method validation in drug analysis using electroanalytical techniques	(72)
Svorc	2013	Determination of caffeine: A comprehensive review on electrochemical methods	(73)

References

(1) Anal. Chim. Acta 119 (1980) 1-24
(2) Anal. Chem. 59 (1987) 379A-390A
(3) Electroanalysis 2 (1990) 419-433
(4) Anal. Chim. Acta 249 (1991) 43-54
(5) Analyst 118 (1993) 433-438
(6) Electroanalysis 7 (1995) 5-22
(7) Anal. Chim. Acta 305 (1995) 3-7
(8) Electroanalysis 7 (1995) 23-45
(9) Adv. Mol. Cell Biol. 15B (1996) 421-450
(10) Chem. Listy 93 (1999) 490-499
(11) Chemija 11 (2000) 18-27
(12) Fres. J. Anal. Chem. 366 (2000) 560-568
(13) Fres. J. of Anal. Chem. 369 (2001) 556-562
(14) Fres. J. of Anal. Chem. 370 (2001) 1-10
(15) Crit. Rev. Anal. Chem. 31 (2001) 311-345
(16) Electroanalysis 15 (2003) 1073-1085
(17) Sensors 5 (2005) 317-342
(18) Chem. Commun. (2005) 829–841
(19) Analyst, 131 (2006) 15–21
(20) Encyclopedia Sens. 4 (2006) 283-429

(21) Curr. Pharm. Anal. 2 (2006) 1-8
(22) Electroanalysis 19 (2007) 2003-2014
(23) Int. J. Electrochem. Sci. 2 (2007) 355 - 385
(24) J. Solid State Electrochem. 12(2008) 1185-1204
(25) Microchim. Acta 163 (2008) 23-31
(26) Curr. Anal. Chem. 4 (2008) 242-249
(27) Sensors 8 (2008) 1400-1458
(28) Crit. Rev. Anal. Chem. 39 (2009) 204-227
(29) Monatsh. Chemie 140 (2009) 861-889
(30) Cent. Eur. J. Chem. 7 (2009) 598-656
(31) Electroanalysis 21 (2009) 7-28
(32) J. Serb. Chem. Soc. 74 (2009) 1021-1033
(33) Electroanalysis 22 (2010) 1937-1946.
(34) J. Electroanal. Chem. 656 (2011) 2-16
(35) Curr. Anal. Chem. 7 (2011) 80-100
(36) Anal. Bioanal. Chem. 405 (2013) 3525-3539
(37) Anal. Chim. Acta 500 (2003) 247-257
(38) Chem. - A Eur. J. 9 (2003) 3732-3739
(39) Electroanalysis 17 (2005) 7-14
(40) Front. Biosci. 10 (2005) 492-505
(41) Anal. Sci. 21 (2005) 1263-1268
(42) Anal. Bioanal. Chem. 382 (2005) 884-886
(43) TrAC, Trends in Anal. Chem. 25 (2006) 480-489
(44) Microchim. Acta 152 (2006)187–214
(45) Electroanalysis 19 (2007) 769-776
(46) Electroanalysis 20 (2008) 583-601
(47) TrAC, Trends in Anal. Chem. 27 (2008) 568-584
(48) Curr. Anal. Chem. 4 (2008) 177-182
(49) Electrochim. Acta 53 (2008) 5848-5866
(50) Langmuir 25 (2009) 13840-13851
(51) Analyst 134 (2009) 18-24
(52) Sensors 9 (2009) 5368-5378
(53) Dia. Rel. Mat. 18 (2009) 1401-1420
(54) J. Nanosci. Nanotechnol. 9 (2009) 2201-2214
(55) Electroanalysis 22 (2010) 1947-1956
(56) Anal. Chim. Acta 662 (2010) 105-127
(57) Sens. Actuators, B: Chemical 145 (2010) 417-427
(58) Carbon Nanotubes (2011) 395-412
(59) Anal. Lett. 45 (2012) 783-803
(60) Chem. Rec. 12 (2012) 201-213
(61) Anal. Bioanal. Chem. 405 (2013) 3431-3448
(62) J. Pharm. Biomed. Anal. 4 (1986) 789-797
(63) J. Pharm. Biomed. Anal. 7 (1989) 1323-1335
(64) Anal. Chim. Acta 273 (1993) 329-334
(65) Electroanalysis 6 (1994) 617-24
(66) Fres. J. Anal. Chem. 360 (1998) 1-9
(67) Anal. Lett. 37 (2004) 831-844
(68) Curr. Pharm. Anal. 2 (2006) 1-8
(69) Anal. Lett. 40 (2007) 817-853
(70) J. Pharm. Sci. 99 (2010) 4784-4804
(71) Anal. Biochem. 408 (2011) 179-196
(72) Open Anal. Chem. J. 5 (2011) 1-21
(73) Int. J. Electrochem. Sci. 8 (2013) 5755-5773

Table S2. List of ATC code N drugs, their pK_a values and in-vivo half lifetime

**Drug**	**ATC code**	**pK_a value**	**In-vivo half life**
ATC code N01			
Procaine	N01BX04	8.05	40-84 s
CAP	N01Bx04	9.76	1.64 h
ATC code N02			
Paracetamol	N02BE01	9.5	2-3 h
Aspirin	N02BA01	3.5	3.2 h
Morphine	N02AA02	8.2 and 9.9	2-3 h
Tramadol	N02AX03	9.41	5.5-7 h
Sumatriptan	N02CC01	9.63 and >12	2.5 h
Codeine	N02AA59	8.2	2.5-3 h
Benorilate	N02BA10	converted to aspirin and paracetamol	2-10 min
ATC code N03			
Carbamazepine	N02AF01	13.9	25-65 h
Gabapentin	N03AX12	3.68 and 10.70	5-7 h
Lamotrigine	N03AX09	5.5	24-34
ATC code N04 and Neurotransmitters			
Levodopa	N04BA02	2.32, 8.72, 9.72 and 13.4	50 min
Carbidopa	N04BA02	2.3 and 7.8	1.5 h
Dopamine	C01CA04	8.93 and 10.41	2 min
Epinephrine	C01CA24	8.55	2 min
Norepinephrine	C01CA03	9.57	2 min
Serotonin	---	9.97 and 10.73	3 h
ATC code N05			
Buspirone	N05BE01	1.22 and 7.32	2-3 h
Chlorpromazine	N05AA02	9.15	16-30 h
Clozapine	N05AH02	3.7 and 7.6	14.2 h
Risperidone	N05AX10	8.24	20 h
Thioridazine	N05AC02	9.5	7-13 h
ATC code N06			
Caffeine	N06BC01	0.7	5 h
Clomipramine	N06AA04	9.5	32 h
Desipramine	N06AA01	1.5 and 10.2	21-23 h
Imipramine	N06AA02	9.5	11-25 h
Trimipramine	N06AA06	8.0	11-23 h
Trazodone	N06AX05	6.74	3-6 h
ATC code N07			
Cinnarizine	N07CA02	2.59 and 7.88	3-4 h
Dextromethorphan	N07XX59	8.3	3-5 h
Naltrexone	N07BB04	820 and 9.63	4 h
Nicotine	N07BA01	3.12 and 8.02	2h

Table S3. Electrochemical reaction mechanisms and electron transfer processes of the drugs included in this review

**Drug**	**Reaction mechanism**
ATC code N01	
Procaine	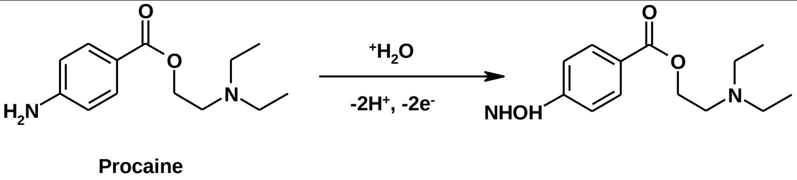
Capsaicin	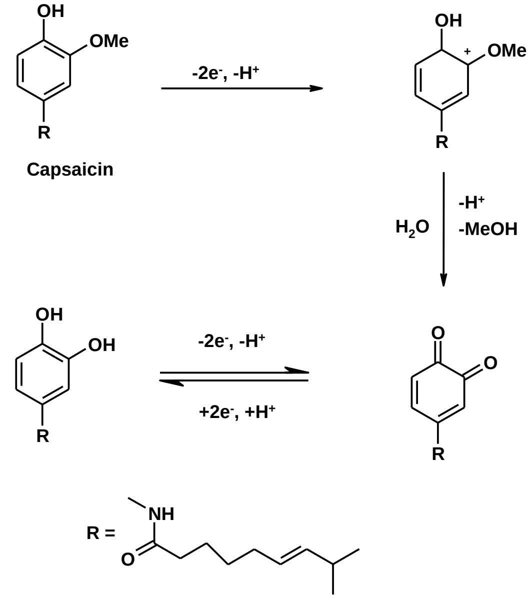

ATC code N02	
Paracetamol	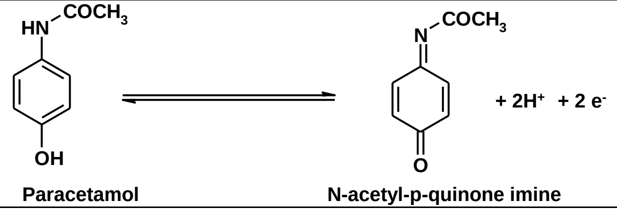
Aspirin	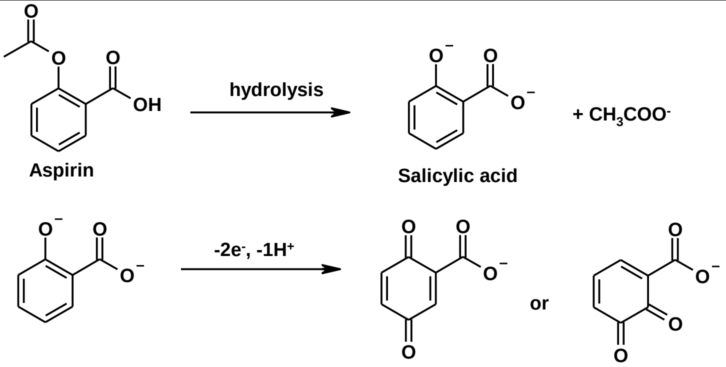

Morphine	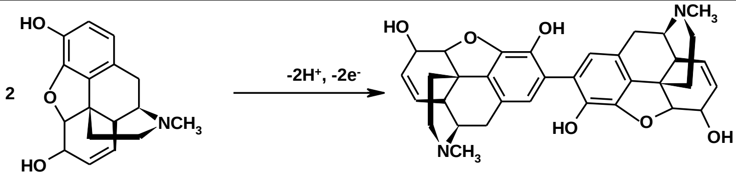 Morphine
Tramadol	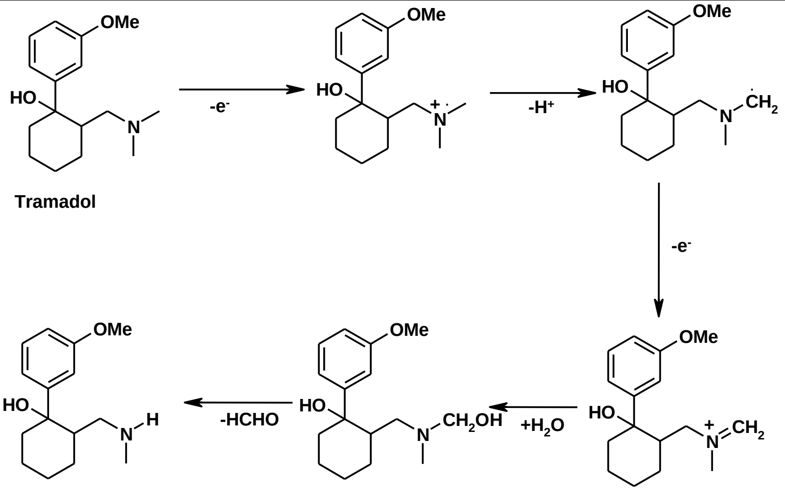
Sumatriptan	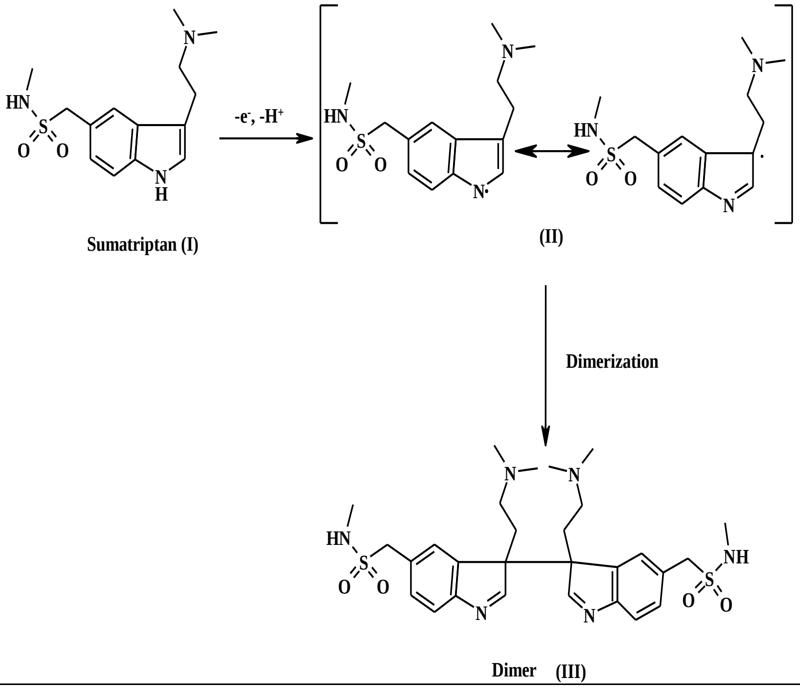
Codeine	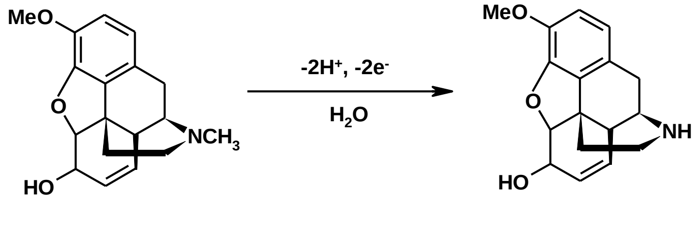 Codeine Norcodeine

<table>
<tr><td>Benorilate</td><td>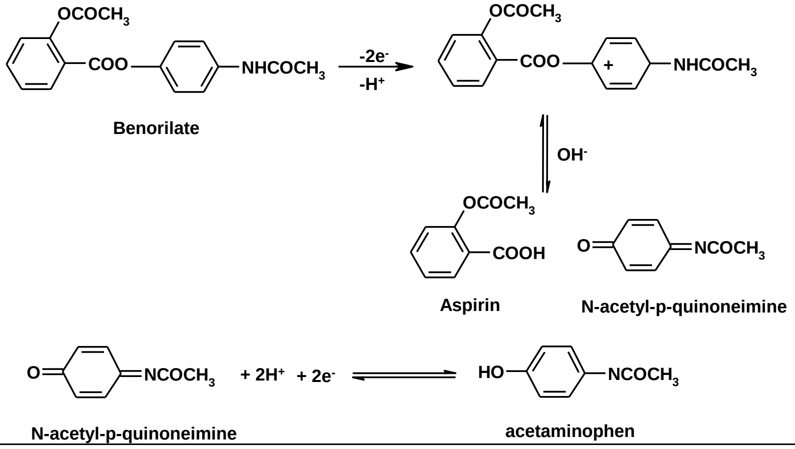
</td></tr>
</table>

<table>
<tr><td colspan="2">ATC code N03</td></tr>
<tr><td>Carbam-azepine</td><td>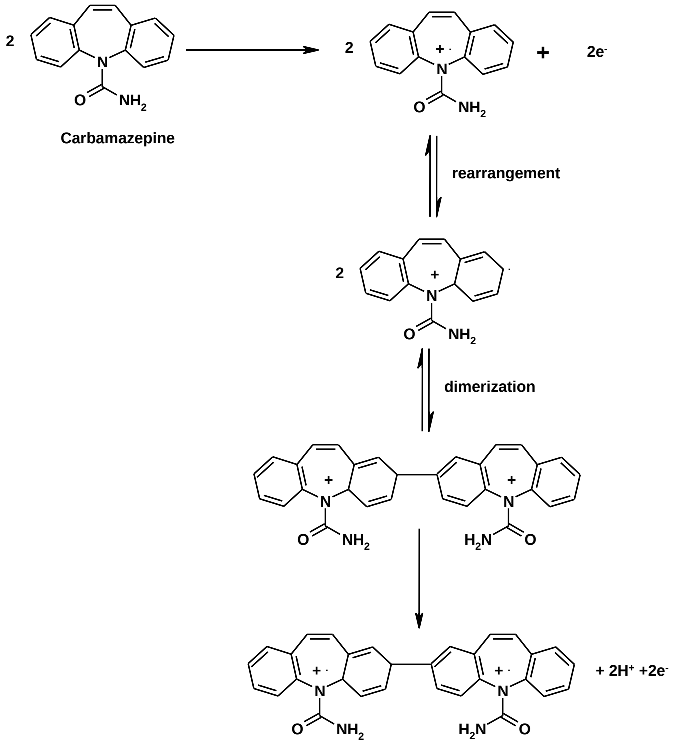
</td></tr>
<tr><td>Gabapentin</td><td>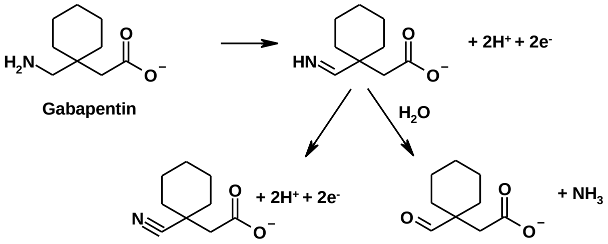
</td></tr>
</table>

Lamotrigine	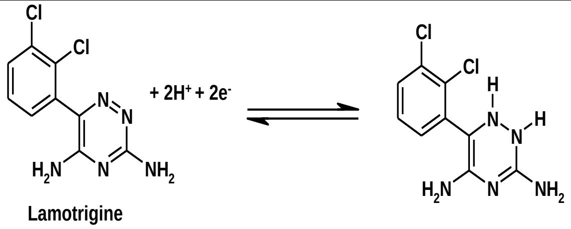

ATC code N04	
Levodopa, Dopamine, Norepi-nephrine	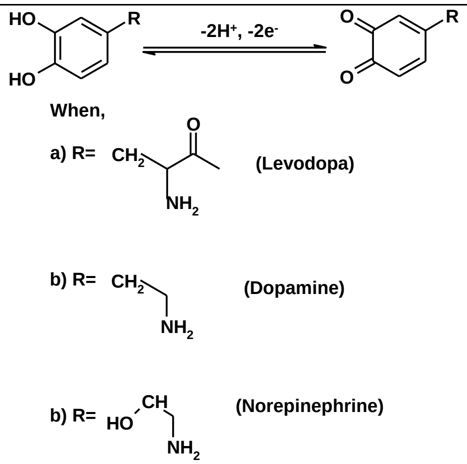
Carbidopa, Epinephrine	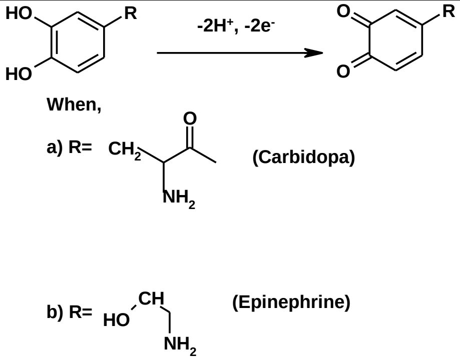

<table>
<tr><td>Catechol-amines (in detailed mechanism)</td><td>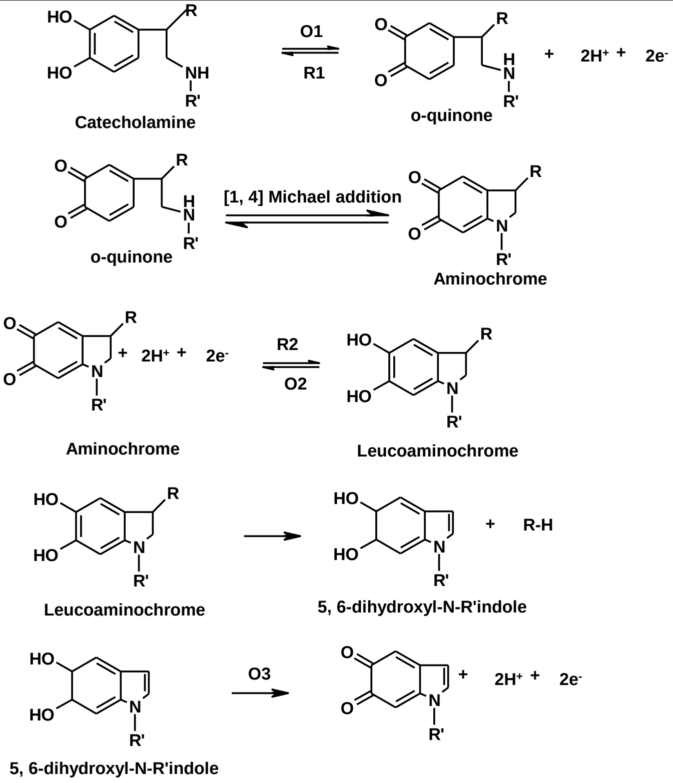

In the above catecholamines, R and R’ are, respectively:
Dopamine: H, H; L-dopa: H, H (has a –COOH group in its structure); epinephrine: OH, CH3; norepinephrine: OH, H.</td></tr>
<tr><td>Serotonin</td><td>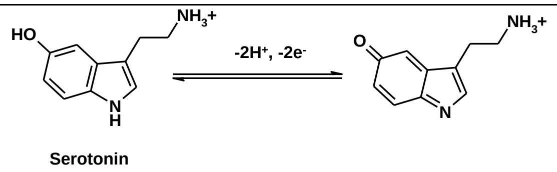
</td></tr>
</table>

<table>
<tr><td colspan="2">ATC Code N05</td></tr>
<tr><td>Buspirone</td><td>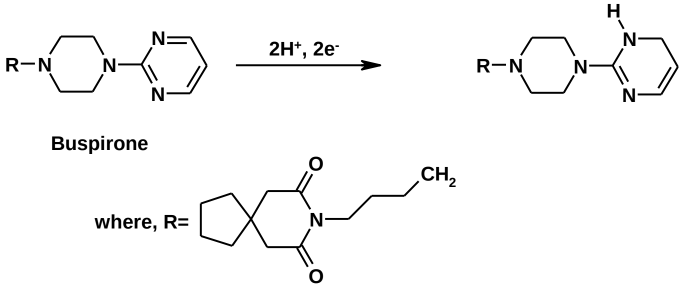
</td></tr>
</table>

<table>
<tr><td>Chlor-promazine</td><td>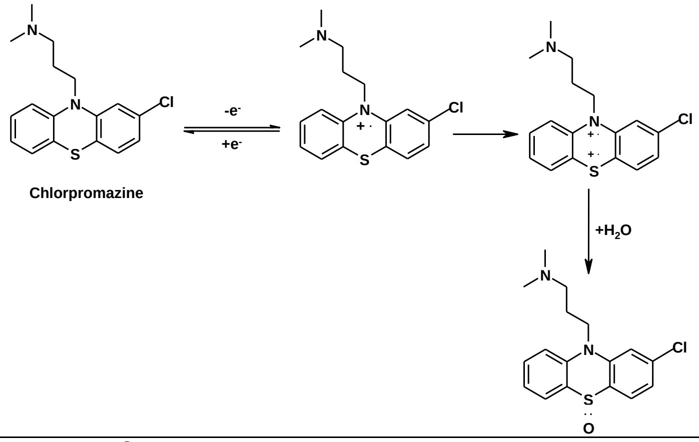
</td></tr>
<tr><td>Thioridazine</td><td>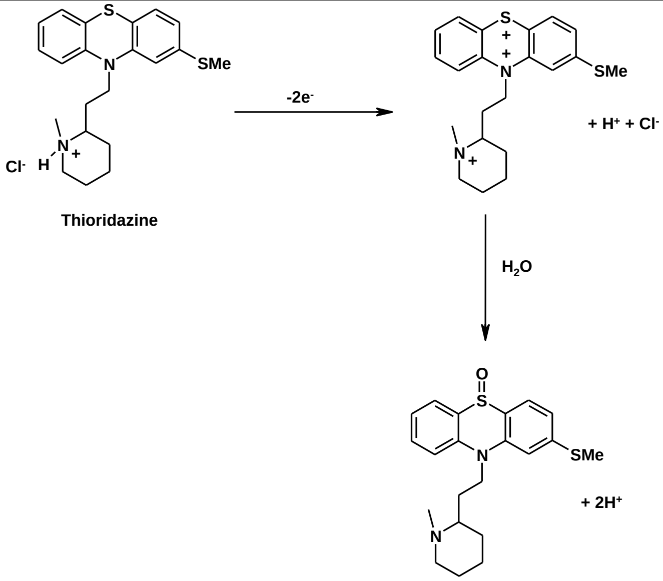
</td></tr>
</table>

<table>
<tr><td colspan="2">ATC code N06</td></tr>
<tr><td>Caffeine</td><td>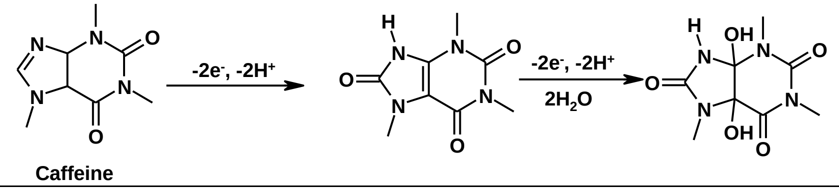
</td></tr>
</table>

<table>
<tr>
<td>Imipramine, trimipramine, desipramine, clomipramine</td>
<td>
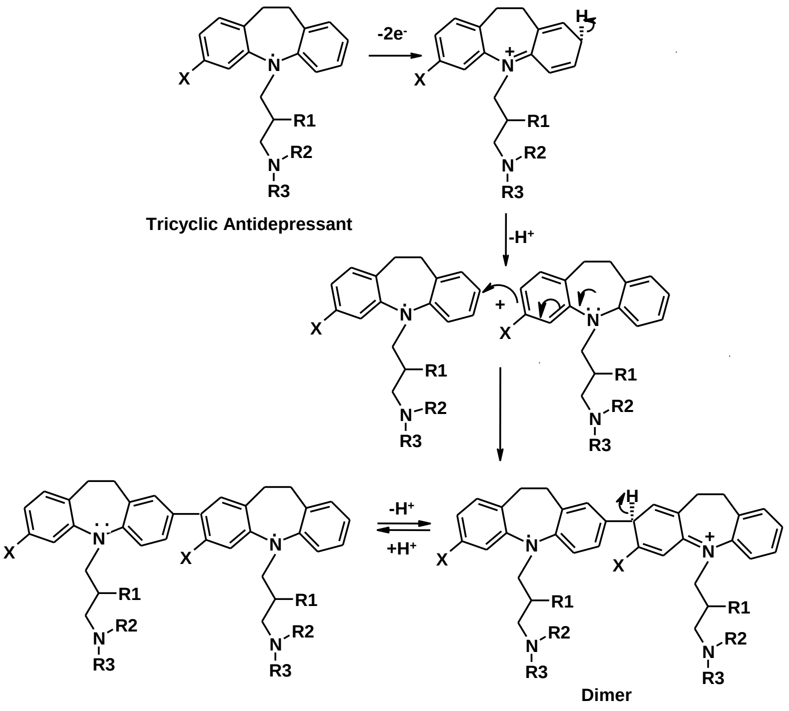

Where,

<table>
<tr><th></th><th>R1</th><th>R2</th><th>R3</th><th>X</th><th></th></tr>
<tr><td>a)</td><td>H</td><td>H</td><td>CH_3</td><td>H</td><td>(Desipramine)</td></tr>
<tr><td>b)</td><td>H</td><td>CH_3</td><td>CH_3</td><td>H</td><td>(Imipramine)</td></tr>
<tr><td>c)</td><td>CH_3</td><td>CH_3</td><td>CH_3</td><td>H</td><td>(Trimipramine)</td></tr>
<tr><td>d)</td><td>H</td><td>CH_3</td><td>CH_3</td><td>Cl</td><td>(Clomipramine)</td></tr>
</table>
</td>
</tr>
</table>

Trazodone	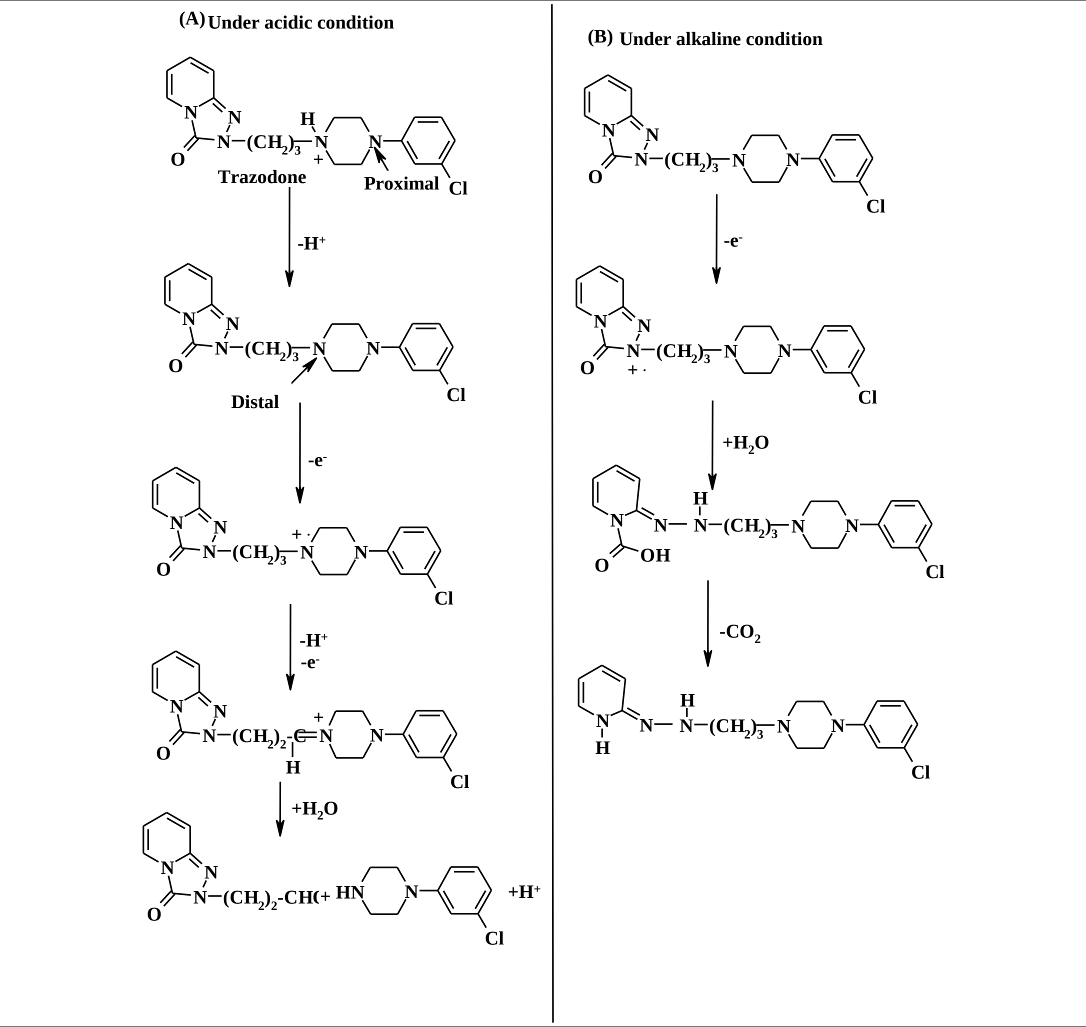
Venlafxine and Desvenlafaxine	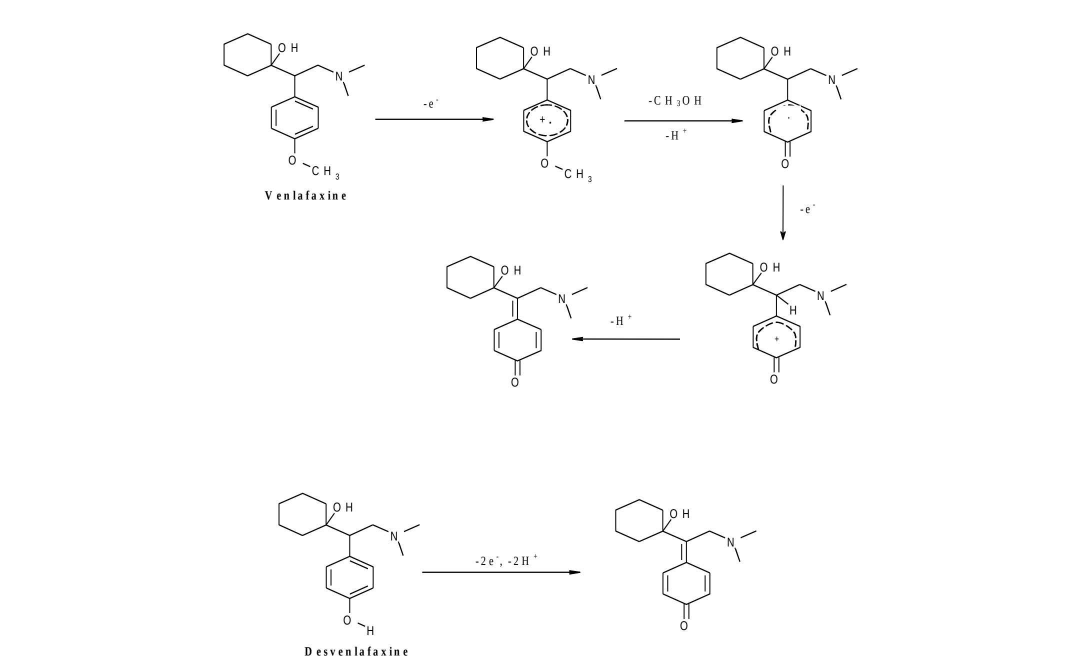

ATC code N07	
Cinnarizine	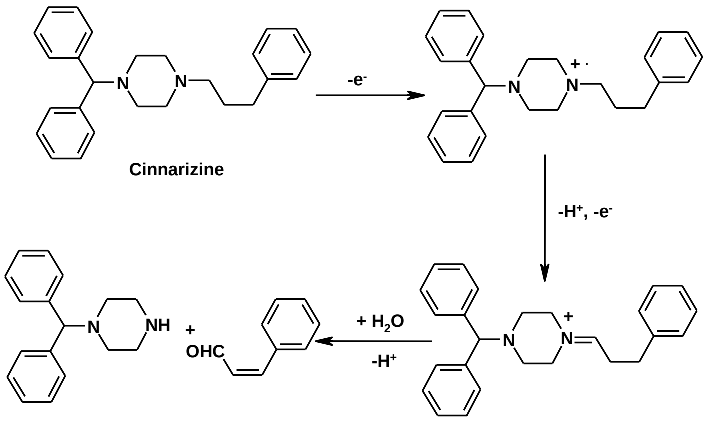
Dextro-methorphan	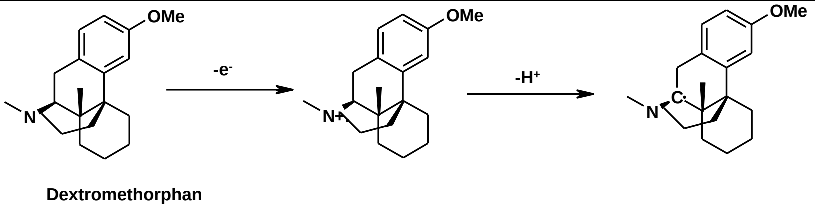
Naltrexone	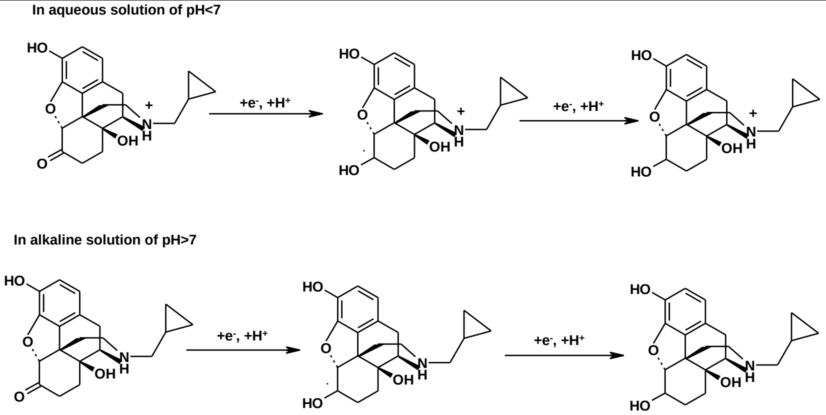
Nicotine	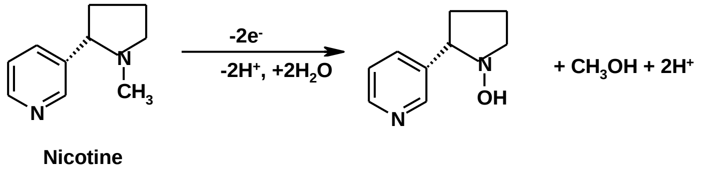

Table S4. Electroanalytical figures of merit for ATC code N01 drugs. M: method; EM: electrode material; LOD: limit of detection; LWR: linear working range; P: peak potential; SE: supporting electrolyte. References are presented as footnotes below the table.

Drug (Analyte)	*Significant Details and Figures of Merit*	*Ref.*
Procaine	M: LSV; EM: MWCNT film modified glassy carbon electrode; LWR: 0.5-100 µM; LOD: 0.2 µM; SE: pH 7.0 phosphate buffer; applied to analysis of procaine in injection	(1)
Procaine	M: DPV; EM: poly(amidosulfonic acid) and MWCNT/GCE ; LWR: 0.08-1 µM; LOD: 25 nM; SE: pH 7.0 phosphate buffer; applied to analysis of procaine in pharmaceutical formulations	(2)
Capsaicin	M: AdSV; EM: MWCNT/BPPGE; LWR: 0.5 to 15 µM and 15 to 60 µM; LOD: 0.31 µM; SE: pH 1.0 B. R. buffer; P: 0.47 V; applied to determination of capsaicin in hot pepper sauces	(3)
Capsaicin	M: AdSSWV; EM: pencil graphite electrode; LWR: 0.016-0.32 µM; LOD: 3.7 nM; SE: pH 9.0; B. R. buffer; P: 0.42 V; applied analysis of capsaicin in pepper flakes	(4)

References

(1) Bioelectrochemistry 68 (2006) 144 – 149
(2) Microchim. Acta 169 (2010)153–159
(3) Analyst 133 (2008) 888-895
(4) Talanta 112 (2013) 11-19.

Table S5. Electroanalytical figures of merit for ATC code N02 drugs. M: method; EM: electrode material; LOD: limit of detection; LWR: linear working range; P: peak potential; SE: supporting electrolyte. References for Table S5 are present as footnotes below the table.

Drug (Analyte)	*Significant Details and Figures of Merit*	*Ref.*
Paracetamol	M: AdSDPV; EM: BPPGE modified with MWCNT; LWR: 0.01 µM to 2 µM and 2 µM to 20 µM; LOD: 10 nM; SE: pH 7.5 ; P: 0.3 V; applied to determination in pharmaceutical formulations	(1)
Paracetamol	M: DPV; EM: CPE modified with cetylpyridinium bromide and MWCNT; LWR: 5 µM to 92.6 µM; LOD: 0.57 µM; SE: pH 7.7 ; P:0.4 V; applied to simultaneous determination of paracetamol and uric acid in urine	(2)
Paracetamol	M: DPV; EM: CPE modified with ZrO_2 nanoparticles; LWR: 1 µM to 2500 µM; LOD:0.91 µM ; SE: pH 7.0 ; P: 0.47 V; applied to simultaneous determination of paracetamol, nor epinephrine and folic acid in injection fluids	(3)
Paracetamol	M: DPV; EM: GCE modified with poly (4-vinylpyridine) and multiwalled carbon nanotubes; LWR: 0.02–450 µM; LOD: 1.69 nM ;SE: pH 7.0 ; P: 0.40 V; applied determination of paracetamol in tablets and urine	(4)
Paracetamol	M: DPV; EM: GCE modified with Nafion/TiO_2 NPs and graphene composite; LWR: 1–100 µM; LOD: 0.21 µM ;SE: pH 7.0 ; P:0.47 V; applied determination of paracetamol in tablets and serum	(5)
Paracetamol	M: DPV; EM:MWCNTs-GNS/GCE; LWR: 0.8–110 µM; LOD:0.1 µM ; SE: pH 7.5 ; P:0.33 V; applied to simultaneous determination of paracetamol and tyrosine in tablets and serum	(6)
Paracetamol	M: DPV; EM: $LaNi_{0.5}$-$Ti_{0.5}O_3$/$CoFe_2O_4$ nanoparticle-modified electrode (LNT–CFO / GCE) ; LWR: 0.5–901 µM; LOD: 0.19 µM ; SE: pH 7.0 ; applied to determination of paracetamol in tablets	(7)
Paracetamol	M: DPV; EM: MWCNT dispersed in polyhistidine on GCE; LWR: 0.25–10 µM; LOD: 32 nM ; SE: pH 7.4 ; simultaneous determination of paracetamol and ascorbic acid; applied to determination of paracetamol in tablets	(8)
Paracetamol	M: DPV; EM: Fe_3O_4 nanoparticles-coated poly(diallyldimethylammonium chloride)-functionalized graphene nanocomposite film on GCE; LWR: 0.1–100 µM; LOD: 37	(9)

	nM; SE: pH 7.0 B.R. buffer; simultaneous determination with ascorbic acid; applied to determination of paracetamol in tablets, urine and blood serum	
Paracetamol	M:amperometry; EM: Cobalt hydroxide nanoparticles/GCE; LWR: 50-550 µM ; LOD: 1.83 µM ; SE: 0.1 N NaOH; analysis of aspirin in pharmaceutical formulations and urine	(10)
Paracetamol	M: AdSDPV; EM: in situ surfactant (Triton X100) modified MWCNT paste electrode; LWR: 0.291–100 µM; LOD: 25.8 nM; SE: pH 7.0 ; simultaneous determination of paracetamol, aspirin and caffeine ; applied to determination of paracetamol in tablets, urine and blood serum	(11)
Aspirin	M: SWV; EM: BDDE; LWR: 2.5-105 µM; LOD: 2.0 µM; SE: 0.01M sulfuric acid; analysis of aspirin in pharmaceutical formulations	(12)
Aspirin	M: AdSSWV; EM: Montmorillonite modified GCE; LWR: 0.22 to 1.67 µM; LOD: 0.11 µM; SE: pH 1.0; simultaneous determination of aspirin, paracetamol and dipyrone ; analysis of aspirin in formulations and urine	(13)
Aspirin	M:AdSSWV; EM: Boron doped diamond electrode ;LWR: 11.1 to 1500 µM ; LOD: 0.23 µM; SE: 0.1 M sulfuric acid; analysis of aspirin in pharmaceutical formulations and urine	(14)
Aspirin	M: amperometry; EM: Cobalt hydroxide nanoparticles/GCE; LWR: 100-700 µM; LOD: 1.88 µM; SE: 0.1 M NaOH; analysis of aspirin in pharmaceutical formulations and urine	(15)
Aspirin	M: SWV; EM: multiwalled carbon nanotube-aluminacoated silica nanocomposite modified glassy carbon electrode; LWR: 15-65 µM; LOD: 3.77 µM ; SE: pH 1.0 sulfuric acid; analysis of aspirin in pharmaceutical formulations	(16)
Aspririn	M: CV; EM: NiO nanoparticles on BPPGE; LWR: 15-65 µM; LOD: 30 µM; SE: pH 1.0 sulfuric acid; analysis of aspirin in pharmaceutical formulations	(17)
Aspirin	M: AdSDPV; EM: in situ surfactant (Triton X100) modified MWCNT paste electrode; LWR: 0.291-100 µM; LOD: 84.7 nM; SE: pH 7.0 ; simultaneous determination of paracetamol, aspirin and caffeine ; applied to determination of aspirin in tablets, urine and blood serum	(18)
Morphine	M: DPV; EM: AuNPs electrodeposited onto Nafion coated GCE; LWR: 0.2-260 µM; LOD: 1.33 nM; SE: pH 7.4, B.R. buffer; P: 0.42 V; analysis of morphine in urine	(19)
Morphine	M: DPV; EM: AuNPs modified CPE; LWR: 0.4-200 µM; LOD: 4.21 nM; SE: pH 7.4, B.R. buffer; P: 0.48 V; analysis of morphine in urine	(20)
Morphine	M: DPV; EM: AuNPs/ferrocene modified CPE; LWR: 1-1800 µM; LOD: 3.51 nM; SE: pH 7.4, B.R. buffer; P: 0.40 V; analysis of morphine in urine	(21)
Morphine	M: DPV; EM: multiwall carbon nanotubes and carbon ionic liquid (1-butyl-3-methylimidazolium hexafluoro phosphate) modified CPE; LWR: 0.45-450 µM; LOD: 0.14 µM; SE: pH 8.0 ; P: 0.52 V; analysis of morphine in ampoule and urine	(22)
Morphine	M: DPV; EM: 4-hydroxy-2-(triphenylphosphonio) phenolate and multi-wall carbon nanotubes modified CPE; LWR: 1-950 µM; LOD: 0.066 µM; SE: pH 7.0; P: 0.15 V; analysis of morphine in formulations and urine	(23)
Morphine	M: DPV;EM: A carbon paste electrode modified with multiwall carbon nanotubes and n-hexyl-3-methylimidazolium hexafluoro phosphate; LWR: 0.6-600 µM; LOD: 0.02 µM; SE: pH 7.0, ; P: 0.48 V; analysis of morphine in ampoules and urine	(24)
Morphine	M:SWV; EM: ZnO/CNT nanocomposite and 1-methyl-3-butylimidazolium bromide modified carbon paste electrode; LWR: 0.1-700 µM; LOD: 0.06 µM; SE: pH 8.0 ; P: 0.52 V; analysis of morphine in ampoules and urine	(25)
Morphine	M: SWV; EM: vinylferrocene /multiwall carbon nanotubes paste electrode; LWR: 0.2-250 µM; LOD: 0.09 µM; SE: pH 7.0, ; P: 0.40 V; analysis of morphine in formulations and urine	(26)
Morphine	M: DPV; EM: AuNPs/ferrocene modified CPE; LWR: 1-240 µM; LOD: 0.65 µM; SE: pH 7.0 ; P: 0.40 V; analysis of morphine in serum and urine	(27)
Morphine	M: DPV; EM: graphene nanosheet-modified GCE; LWR: 1-65 µM; LOD: 0.40 µM; SE: pH 8.0 ; P: 0.37 V; simultaneous determination of morphine, noscapine and heroin	(28)

Morphine	M: amperometry; EM: MWCNT modified preheated GCE; LWR: 0.5-150 µM; LOD: 0.2 µM; SE: pH 7.0 ; P: 0.52 V; analysis of morphine *and* codeine;	(29)
Tramadol	M: SWV; EM: nano-molecularly imprinted polymer and MWCNT modified CPE; LWR: 0.01-20 µM; LOD: 4 nM; SE: pH 7.0 B.R. buffer; P: 0.80 V; analysis of tramadol in tablets and urine	(30)
Tramadol	M: DPV; EM: carbon nanoparticles modified glassy carbon electrode; LWR: 10-1000 µM; LOD: 1.0 µM; SE: pH 7.0 ; P: 0.61 V; analysis of tramadol in tablets and blood plasma; simultaneous determination of tramadol and paracetamol	(31)
Tramadol	M: DPV; EM: glassy carbon electrode modified with electrodeposited gold nanoparticles onto carbon nanotubes; LWR: 10-450 µM and 600-1000 µM; LOD: 0.068 µM; SE: pH 7.0 ; P: 0.75 V; analysis of tramadol in tablets	(32)
Tramadol	M: DPV; EM: multi-walled carbon nanotube-modified glassy carbon electrode; LWR: 2-300 µM; LOD: 0.361 µM; SE: pH 7.5 ; P:0.65 V; analysis of tramadol in tablets, urine and blood serum; simultaneous determination of paracetamol and TRA	(33)
Tramadol	M: AdSSWV; EM: glassy carbon paste electrode modified with Dowex50wx2 and gold nanoparticles; LWR: 0.033-42.2 µM; LOD: 0.011 µM; SE: pH 6.0 acetate buffer; P: 0.75 V; simultaneous determination of tramadol and praracetamol in tablet serum and urine	(34)
Codeine	M: DPV; EM: unmodified boron-doped diamond film electrode; LWR: 0.1-60 µM; LOD: 0.08 µM; SE: pH 7.0 B.R. buffer; P: 1.0 V; analysis of codeine in tablets and urine	(35)
Codeine	M: DPV; EM: multi-walled carbon nanotube modified glassy carbon electrode; LWR: 5-240 µM; LOD: 0.2 µM; SE: pH 7.0 ; analysis of codeine in formulation, urine and serum	(36)
Benorilate	M: DPV;EM: carbon paste electrode; LWR: 0.2-450 µM; LOD: 0.05 µM; SE: pH 6.88 ; P: 1.0 V; analysis of benorilate in tablets	(37)
Benorilate	M: DPV; EM: AgNPs modified CPE; LWR: 0.1-250 µM; LOD: 0.01 µM; SE: pH 6.88 ; P: 0.97 V; analysis of benorilate in tablets and urine	(38)
Sumatriptan	M: LSV; EM: new coccine as dopant anion on the surface of the multi-walled carbon nanotubes pre-coated glassy carbon electrode; LWR: 0.02-10 µM; LOD: 6 nM; SE: pH 7.4 B. R. buffer; P: 0.73 V; analysis of sumatriptan in formulation and blood serum	(39)
Sumatriptan	M: DPV; EM: multi-walled carbon nanotube and silver nanoparticles modified pyrolytic graphite electrode; LWR: 0.08-100 µM; LOD: 0.04 µM; SE: pH 7.4 B. R. buffer; P: 0.72 V; analysis of sumatriptan in tablets	(40)

References

(1) Anal. Chim. Acta 618 (2008) 54-60
(2) J. Electroanal. Chem. 696 (2013) 52–58
(3) Sens. Actuators, B 151 (2010) 243–249
(4) Anal. Chim. Acta 765 (2013) 70–76
(5) Colloids Surf., B: Biointerfaces 85 (2011) 289–292
(6) Colloids Surf., B: Biointerfaces 103 (2013) 84–93
(7) J. Solid State Electrochem. (2012) 16:1635–1642
(8) Sens. Actuators, B 173 (2012) 732–736
(9) Talanta 88 (2012) 181-186
(10) J. Solid State Electrochem. 12 (2008) 1117–1128
(11) Electrochim. Acta 55 (2010) 8638–8648
(12) J. Braz. Chem. Soc. 20 (2009) 360-366
(13) Appl. Clay Sci. 42 (2008) 206–213
(14) Electroanalysis 24 (2012) 1141–1146
(15) J. Solid State Electrochem. 12 (2008) 1117–1128
(16) Sens. Actuators, B 148 (2010) 590-594
(17) Electrochem. Commun. 10 (2008) 1129–1131
(18) Electrochim. Acta 55 (2010) 8638–8648
(19) Analyst 136 (2011) 4682-4691

(20) Int. J. Electrochem. Sci. 6 (2011) 5066-5081
(21) Int. J. Electrochem. Sci. 7 (2012) 10501-10518
(22) Ionics 17 (2011) 659–668
(23) J. Electroanal. Chem. 665 (2012) 45–51
(24) J. Mol. Liq. 174 (2012) 42–47
(25) J. Mol. Liq. 181 (2013) 8–13
(26) Sens. Actuators, B 169 (2012) 96-105
(27) Anal. Methods 3 (2011) 2400-2405
(28) Biosens. Bioelectron. 31 (2012) 205– 211
(29) Electroanalysis17 (2005) 873-879
(30) Biosens. Bioelectron. 44 (2013) 34–40
(31) Electrochim. Acta 55 (2010) 2752–2759
(32) Electroanalysis 24 (2012) 2135–2146
(33) J. Braz. Chem. Soc., 22 (2011) 1549-1558
(34) Anal. Chim. Acta 706 (2011) 246– 254
(35) Electrochim. Acta 87 (2013) 503– 510
(36) Sens.Lett. 10 (2012) 1039-1046
(37) Anal. Lett. 38 (2005) 893–905
(38) Talanta 67 (2005) 625–633
(39) Electrochim. Acta 56 (2011) 10032–10038
(40) Talanta 80 (2009) 31–38

Table S6. Electroanalytical figures of merit for ATC code N03 drugs. M: method; EM: electrode material; LOD: limit of detection; LWR: linear working range; P: peak potential; SE: supporting electrolyte. References are given as footnotes below the table.

Drug (Analyte)	*Significant Details and Figures of Merit*	*Ref.*
Carbamazepine	M: LSV; EM: MWCNT and dihexadecyl hydrogen phosphate/GCE; LWR: 0.13-1.60 µM; LOD: 40 nM; SE: pH 6.9 ; P:1.1 V; applied to analysis of carbamazepine in pharmaceutical formulations and waste water .	(1)
Carbamazepine	M: DPV; EM: fullerene-C60 modified GCE; LWR:0.09-10 µM ; LOD:17 nM ; SE: P:1.2 V; applied to the determination of CBZ in pharmaceutical formulations, spiked human serum and urine .	(2)
Gabapentin	M: SWV; EM: AgNP and MWCNT modified CPE ; LWR: 0.0031 to 29000 µM ; LOD:0.56 nM ; SE: pH 10 citrate buffer; P: -0.1 V; applied to determination in formulations and plasma	(3)
Gabapentin	M: amperometry; EM:glassy carbon electrode modified with $LaFeO_3$ nanoparticles; LWR: 2.4-50 µM; LOD: 0.3 µM ; SE: 100 mM NaOH; analysis of GBP in urine and blood serum	(4)
Lamotrigine	M: CV; EM: pyrolytic graphite electrode ; LWR: 0.1-100 µM; LOD:0.08 µM ; SE: pH 7.0 ; P:0.1 V; applied to determination of lamotrigine in pharmaceutical formulations and blood plasma	(5)
Lamotrigine	M: AdSDPV; EM: silver nanoparticle-modified carbon screen-printed electrodes ; LWR: 0.5–15µM and 0.33–1.5 µM; LOD: 0.372 µM ; SE: pH 5.5 B.R. Buffer; P:-1.12 V; applied to determination of lamotrigine in tablets	(6)

References

(1) Anal. Chim. Acta 674 (2010) 182–189
(2) Electrochim. Acta 56 (2011) 5295–5301
(3) Electroanalysis 23 (2011) 2949 – 2954
(4) J. Solid State Electrochem. 16 (2012) 45–52
(5) Bioelectrochemistry 84 (2012) 38–43
(6) Talanta 74 (2007) 59–64

Table S7. Electroanalytical figures of merit for ATC code N04 drugs. M: method; EM: electrode material; LOD: limit of detection; LWR: linear working range; P: peak potential; SE: supporting electrolyte. References for Table S7 are present as footnotes below the table.

Drug (Analyte)	***Significant Details and Figures of Merit***	***Ref.***
Dopamine	M: FSCV; EM: carbon micro fiber electrode; LWR: 0.1 to 10 µM; LOD: 24 nM; SE: pH 7.4 ; P: 0.8 V; applied to analysis of DA in vivo in male Sprague-Dawley rats	(1)
Dopamine	M: FSCV; EM: carbon micro fiber electrode modified with MWCNT; LOD: 17 nM; SE: P: 0.5 V; applied to determination of DA in Drosophilia	(2)
Dopamine	M: DPV; EM: Au-nanoclusters incorporated 3-amino-5-mercapto-1,2,4-triazole film modified GCE; LWR: 0.6 to 340 µM; LOD: 0.05 µM; SE: pH 4.0; P: 0.29 V; applied to simultaneous determination of ascorbic acid, dopamine, uric acid and nitrite in urine and serum	(3)
Dopamine	M: DPV; EM: CPE modified with N,N´(2,3-dihydroxy-benzylidene) - 1,4-phenylenediamine and TiO_2 nanoparticles; LWR: 0.08 to 20 µM; LOD: 31.4 nM; SE: pH 8.0 ; P: 0.16 V; applied to determination in injection fluids	(4)
Dopamine	M: DPV; EM: glassy carbon electrode modified with $LaFeO_3$ nanoparticles; LWR: 0.15–800 µM; LOD: 30 nM; SE: pH 7.0 ; P:0.1 V; analysis of dopamine in injections and urine ; simultaneous determination of dopamine, uric acid and ascorbic acid	(5)
Dopamine	M: DPV; EM: 1-butyl-3-methylimidazolium tetraflouroborate and multi walled carbon nanotubes modified carbon-ceramic electrode ; LWR: 3–130 µM; LOD: 0.87 µM; SE: pH 7.0 ; P: 0.1 V; applied to determination of dopamine in pharmaceutical formulations and blood serum	(6)
Dopamine	M: DPV; EM: glassy carbon electrode modified with palladium nanoparticles, graphene, and chitosan; LWR: 0.5–15 µM and 20–200 µM; LOD: 0.1 µM; SE: pH 4.0 ; P: 0.3 V; applied to simultaneous determination of dopamine, ascorbic acid and uric acid	(7)
Dopamine	M: DPV; EM: composite made from dodecyl sulfate, graphene nanosheets, and SnO_2 nanoparticles; LWR: 0.1–10 µM; LOD: 80 nM ; SE: pH 7.0 ; P: 0.17 V; applied to determination of dopamine in urine	(8)
Dopamine	M: DPV; EM: basal plane pyrolytic graphite electrode modified with 1,4-naphthoquinone/MWCNT; LWR:40-280 µM; LOD:1.9 µM; SE: pH 7.5 ; P: 0.145 V; simultaneous determination with ascorbic acid and uric acid; applied to determination in urine	(9)
Dopamine	M: DPV; EM: nano-Au/DNA/nano-Au/poly safranin T modified GCE; LWR:8-1100 nM; LOD: 0.2 nM; SE: pH 5.0 ; P: 0.2 V; analysis of dopamine in urine and blood serum ; simultaneous determination of dopamine, uric acid, guanine, and adenine	(10)
Dopamine	M: DPV; EM: Nafion/$Ni(OH)_2$ nanoparticles-carbon nanotube composite modified glassy carbon electrode; LWR: 0.05-25 µM; LOD: 0.015 µM; SE: pH 7.0 ; P: 0.15 V; analysis of DA and serotonin in blood serum	(11)
Dopamine	M: DPV; EM: stacked graphene platelet nanofibers /ionic liquid /chitosan modified GCE; LWR: 0.05-240 µM; LOD: 0.05 µM; SE: pH 6 ; P: 0.05 V; simultaneous determination of DA, AA and UA in urine .	(12)
Dopamine	M: anodic stripping DPV; EM: Sol-gel derived multiwalled carbon nanotubes ceramic electrode modified with molecularly imprinted polymer; LWR: 6.5-547 nM; LOD:1 nM; SE: pH 4.8 ; P: 0.17 V; analysis of dopamine in blood serum, cerebrospinal fluid and pharmaceutical	(13)
Dopamine	M: DPV; EM: CuO nanoparticles modified CPE; LWR: 0.4-40 µM; LOD: 55 nM; SE: pH 6.0 ; analysis of DA in presence of AA simultaneously	(14)
Dopamine	M: AdSSWV; EM: Copper(II) complex and silver nanoparticle modified glassy carbon paste electrode; LWR: 2.81-8300 nM; LOD: 0.852 nM; SE: pH 6 acetate buffer; P: 0.15 V; analysis of DA in pharmaceutical formulations, urine and blood	(15)

	serum	
Epinephrine	M: DPV; EM: a polymeric brilliant cresyl blue and dihexadecyl phosphate dispersed multi-walled carbon nanotubes composite film modified glass carbon electrode; LWR: 0.05-10 µM ; LOD: 10 nM; SE: pH 6.0 ; P: 0.25 V; analysis of EP in injections	(16)
Epinephrine	M: DPV; EM: Carbon nanoparticles and derivative of hydroquinone (2,2'-[1,2-ethanediylbis(nitrilomethylidyne)]-bishydroquinone) modified carbon paste electrode ; LWR: 0.5 to 20 µM and 20 to 600 µM; LOD: 1.0 µM; SE: pH 7.0 ; P: 0.2 V; simultaneous determination of epinephrine and acetaminophen	(17)
Epinephrine	M: SWV; EM: edge plane pyrolytic graphite electrode modified with multi-walled carbon nanotubes; LWR: 0.5-100 µM; LOD: 0.15 nM; SE: pH 7.2 ; P: 0.15 V; simultaneous determination of EP, NEP and DA; analysis of EP in urine and serum of smokers	(18)
Epinephrine	M: anodic stripping DPV; EM: CNT-mer dispersed MIP-modified PGE; LWR: 0.49 - 32.2 nM; LOD: 0.1 nM; SE: pH 6.8; P:0.1 V; simultaneous determination of epinephrine, norepinephrine and dopamine; analysis of epinephrine in injections and serum	(19)
Epinephrine	M: DPV; EM: GCE modified with nickel hydroxide nanoparticles / multiwalled carbon nanotubes; LWR: 1-220 µM ; LOD: 0.29 µM; SE: pH 8.0 ammonia buffer; P: 0.03 V; simultaneous determination of epinephrine and piroxicam; analysis of epinephrine in urine	(20)
Epinephrine	M: DPV; EM: over-oxidized polypyrrole/multi-walled carbon Nanotube/GCE ; LWR: 0.1-8 µM and 10-100 µM; LOD: 40 nM; SE: pH 6.0 ; P: 0.23V; determination of epinephrine in presence of ascorbic and uric acid; analysis of epinephrine in pharmaceutical and serum	(21)
Epinephrine	M: DPV; EM: ferrocenedicarboxylic acid and MWCNT modified CPE; LWR: 0.05-450 nM ; LOD: 35 nM; SE: pH 5.0 B. R. buffer; P: 0.32 V; analysis of epinephrine in injection	(22)
Epinephrine	M: CV; EM: GCE modified with graphene/gold nanocomposite ; LWR: 0.05-8.0 µM; LOD: 7 nM ; SE: pH 7.0 ; P: 0.18 V; analysis of epinephrine in injections	(23)
Epinephrine	M: DPV; EM: 1-methyl-3-butylimidazolium bromide modified carbon nanotube paste electrode ; LWR: 1-1800 µM; LOD: 0.09 µM ; SE: pH 7.0 ; P: 0.20 V; simultaneous determination with acetaminophen; analysis of epinephrine in pharmaceutical formulations, urine and blood serum	(24)
Epinephrine	M: AdSSWV; EM: Copper(II) complex and silver nanoparticle modified glassy carbon paste electrode; LWR: 1.32-8250 nM; LOD: 0.396 nM; SE: pH 7 phosphate buffer; P: -0.12 V; analysis of NEP in pharmaceutical formulations, urine and blood serum .	(25)
Norepinephrine	M: DPV; EM: carbon-coated nickel magnetic nanoparticles modified GCE; LWR: 0.2-80 µM ; LOD: 0.06 µM; SE: pH 7.0 ; P: 0.18 V; no analyzed	(26)
Norepinephrine	M: DPV; EM: MWNTs modified edge plane pyrolytic graphite electrode; LWR: 0.5-100 nM; LOD: 0.09 nM; SE: pH 7.2 ; P: 0.15 V; analysis of NEP in serum of smokers and athletes; simultaneous determination of epinephrine and norepinephrine	(27)
Norepinephrine	M: DPV; EM: screen printed carbon electrode modified with polyacrylic acid-coated multi-wall carbon nanotubes; LWR: 0-10 µM; LOD: 0.131 µM ; SE: pH 7.5 ; P:0.15 V; Simultaneous determination of norepinephrine, ascorbic acid and uric acid; analysis of norepinephrine in serum and urine	(28)
Norepinephrine	M: SWV; EM: carbon paste electrode modified with multiwall carbon nanotubes and 1-methyl-3-butylimidazolium chloride; LWR: 0.2-500µM; LOD: 0.08 µM; SE: pH 7.0 ; P: 0.36 V; analysis of norepinephrine in ampoule, urine and blood serum	(29)

Norepinephrine	M: DPV; EM: molybdenum(VI) complex and MWCNT modified CPE; LWR: 0.08-30 µM and 30-700 µM; LOD: 43 nM; SE: pH 7.0 ; P: 0.20 V; simultaneous determination of norepinephrine, folic acid and acetaminophen; analysis of NEP in ampoule and urine .	(30)
Norepinephrine	M: DPV; EM: Ferrocene/MWCNT modified CPE; LWR:0.47-500 µM; LOD: 0.21 µM; SE: pH 7.0, ; P: 0.40 V; simultaneous determination of norepinephrine, acetaminophen and tryptophan; analysis of norepinephrine in ampoule and urine	(31)
Norepinephrine	M: DPV; EM: nanostructured polyaniline doped with tungstophosphoric acid modified CPE; LWR: 0.08-2000 µM; LOD: 50 nM; SE: pH 7.0 ; P: 0.20 V; simultaneous determination of norepinephrine, acetaminophen and folic acid; analysis of NEP in pharmaceutical formulations and blood serum .	(32)
Norepinephrine	M: CV; EM: AgNP doped poly glycine/GCE; LWR: 0.01-20 µM ; LOD: 0.12 µM; SE: pH 6.0 ; P: 0.20 V; analysis of NEP in injection	(33)
Norepinephrine	M: DPV; EM: nanostructured MCM-41 modified CPE; LWR: 0.07-2000 µM; LOD: 0.04 µM; SE: pH 7.0 ; P: 0.21 V; simultaneous determination of norepinephrine, acetaminophen and folic acid in urine	(34)
Norepinephrine	M: AdSSWV; EM: copper(II) complex and silver nanoparticle modified glassy carbon paste electrode; LWR: 1.18 to 8820 nM; LOD: 0.354 nM; SE: pH 7.0 ; P: 0.21 V; analysis of norepinephrine in pharmaceutical formulations, urine and blood serum .	(35)
Serotonin	M: DPV; EM: methylimidazolium hexafluorophosphate and multi-wall carbon nanotubes modified GCE; LWR: 20 to 7000 nM; LOD: 8 nM; SE: pH 7 ; P: 0.35 V; simultaneous determination of dopamine and serotonin ; analysis of serotonin in blood serum	(36)
Serotonin	M:DPV; EM: palladium-functionalized, multi-walled carbon nano-tubes with electrochemical deposition of poly 3,4-ethylenedioxy pyrrole/GCE; LWR: 0.1 to 200 µM; LOD: 5 nM; SE: pH 7.4 ; P: 0.355 V; simultaneous determination of DA and serotonin	(37)
Serotonin	M: DPV; EM: carbon nanofibers; LWR: 1 to 10 µM; LOD: 250 nM; SE: pH 7.4 tris buffer; P: 0.70 V; simultaneous determination of ascorbic acid, dopamine and serotonin	(38)
Serotonin	M: DPV; EM: carbon nanotube/chitosan modified GCE; LWR: 0.5 to 130 µM; LOD: 80 nM; SE: pH 7 ; P: 0.34 V; simultaneous determination of L-dopa and serotonin in urine and blood serum	(39)
Serotonin	M: DPV; EM: carbon ionic liquid electrode modified with $Co(OH)_2$ nanoparticles, multi-walled carbon nanotubes and nafion; LWR: 0.05 to 75 µM; LOD:0.023 µM; SE: pH 7.5 ; P: 0.34 V; simultaneous determination of L-dopa and serotonin in blood serum	(40)
Serotonin	M: DPV; EM: Nafion/$Ni(OH)_2$ nanoparticles-carbon nanotube composite modified GCE; LWR: 0.008-10 µM; LOD: 3 nM; SE: pH 7.0 ; P: 0.34 V; simultaneous voltammetric determination of dopamine and serotonin in blood serum	(41)
Levodopa	M: DPV; EM: Nafion/$Ni(OH)_2$ nanoparticles-carbon nanotube composite modified glassy carbon electrode; LWR: 1-672 µM; LOD: 0.017 µM; SE: pH 7.0 ; P: 0.10 V; simultaneous voltammetric determination of L-dopa and acetaminophen in urine	(42)
Levodopa	M: DPV; EM: meso-tetrakis(3-methylphenyl) cobalt porphyrin (CP) and TiO2 nanoparticles modified CPE; LWR: 0.1-100 µM; LOD: 69 nM; SE: pH 7.0 ; P: 0.45 V; simultaneous voltammetric determination of L-dopa and carbidopa in water, urine and blood serum	(43)
Levodopa	M: DPV; EM: 2, 2'-[1,2-ethanediylbis (nitriloethylidyne)]-bis-hydroquinone and carbon nanotubes modified CPE; LWR: 0.2-35µM and 35-700 µM; LOD: 0.094 µM; SE: pH 7.0 ; P: 0.22 V; simultaneous voltammetric determination of L-dopa, carbidopa and tryptophan in water and urine	(44)
Levodopa	M: SWV; EM: L-Dopa based on $Co(DMG)_2ClPy$/multi-walled carbon nanotubes	(45)

	composite immobilized on basal plane pyrolytic graphite electrode; LWR: 3-100 µM; LOD: 0.86 µM; SE: pH 6.4 ; P: 0.18 V; determination of L-dopa in pharmaceutical formulations	
Levodopa	M: DPV; EM: electrodeposition of quercetin at a multi-walled carbon nanotubes modified GCE; LWR: 0.9-85 µM; LOD: 0.381 µM; SE: pH 7.0 ; P: 0.15 V; simultaneous voltammetric determination of L-dopa, uric acid and tyrosine in urine	(46)
Levodopa	M: DPV; EM: ferrocenedicarboxylic acid and MWCNT modified CPE; LWR: 0.1-1100 µM; LOD:12 nM; SE: pH 5.0 ; P: 0.15 V; simultaneous voltammetric determination of L-dopa, NADH and tryptophan in water and urine	(47)
Levodopa	M: DPV; EM: gold nanoparticle self-assembled carbon nanotube-modified pyrolytic graphite electrode; LWR: 0.1-150 µM; LOD: 50 nM; SE: pH 7.0 ; P: 0.22 V; simultaneous voltammetric determination of L-dopa, ascorbic acid and uric acid in pharmaceutical formulations and urine	(48)
Levodopa	M: DPV; EM: GCE modified with multi-walled carbon nanotube and poly-pyrrole doped with tiron; LWR: 1-100 µM; LOD: 0.1µM; SE: pH 7.0 ; P: 0.18 V; voltammetric determination of L-dopa in blood serum	(49)
Levodopa	M: DPV; EM: gold nanoparticles and nafion/CPE; LWR: 0.2- 20µM and 50-3000 µM; LOD: 1.45 nM; SE: pH 7.4 B. R. buffer; P: 0.18 V; simultaneous voltammetric determination of L-dopa and acetaminophen in urine and blood serum	(50)
Levodopa	M: AdSSWV; EM: Copper(II) complex and silver nanoparticle modified glassy carbon paste electrode; LWR: 7.77-9120 nM; LOD: 2.41 nM; SE: pH 6.0 acetate buffer; P: 0.22 V; analysis of L-dopa in pharmaceutical formulations, urine and blood serum .	(51)
Carbidopa	M: SWV; EM: carbon nanotubes paste electrode modified with 1-methyl-3-butylimidazolium bromide; LWR: 0.1-420 µM; LOD: 60 nM; SE: pH 7.0 ; P: 0.555 V; determination of carbidopa in urine and blood serum	(52)
Carbidopa	M: DPV; EM: ferrocene-modified carbon nanotube paste electrode; LWR: 5-600 µM; LOD: 3.6 µM; SE: pH 7.0 ; P: 0.35 V; determination of carbidopa in urine	(53)
Carbidopa	M: DPV; EM: MWCNT and poly(Nile blue A)/GCE ; LWR: 10- 100 µM; LOD: 1.17 µM; SE: pH 5.0 ; P: 0.21 V	(54)
Carbidopa	M: SWV; EM: ferrocene dicarboxylic acid and MWCNT modified CPE; LWR: 0.1-700 µM; LOD: 65 nM; SE: pH 5.0 ; P: 0.42 V; simultaneous voltammetric determination of carbidopa, folic acid and tryptophan in urine	(55)
Carbidopa	M: SWV; EM: chloranilic acid and MWCNT modified CPE; LWR: 0.6-100 µM; LOD: 0.4 µM; SE: pH 9.0 ; P: 0.1 V; CD deteremined in urine	(56)

References

(1) Sens. Actuators, B 182 (2013) 652– 658
(2) Anal. Chem. 84 (2012) 7816−7822
(3) Biosens. Bioelectron.30 (2011) 315–319
(4) Int. J. Electrochem. Sci. 5 (2010) 147 - 157
(5) Microchim. Acta 164 (2009) 357–362
(6) Cent. Eur. J. Chem. 11 (2013) 1172-1186
(7) J. Electroanal. Chem.695 (2013) 10–16
(8) J. Mater. Chem. B, 1 (2013) 1804–1811
(9) Electroanalysis 25 (2013) 723 – 731
(10) Sens. Actuators, B 178 (2013) 10– 18
(11) Sens. Actuators, B 176 (2013) 543– 551
(12) Talanta 99 (2012) 984–988
(13) Electrochim. Acta 56 (2011) 7202– 7211
(14) Electrochim. Acta 61 (2012) 78– 86
(15) Biosens. Bioelectron. 39 (2013) 124–132
(16) Electroanalysis 20 (2008) 1143–1146
(17) Anal. Methods 4 (2012) 2127-2133
(18) Electrochim. Acta 56 (2011) 2717–2724
(19) Biosens. Bioelectron. 45 (2013) 114–122

(20) Electroanalysis 24 (2012) 2387–2394
(21) Electrochim. Acta 57 (2011) 132–138
(22) Int. J. Electrochem. Sci. 6 (2011) 1307-1316
(23) J. Electroanal. Chem.669 (2012) 35–41
(24) J. Mol. Liq.168 (2012) 69–74
(25) Biosens. Bioelectron.39 (2013) 124–132
(26) Bioelectrochemistry 79 (2010) 1–5
(27) Talanta 84 (2011) 78–83
(28) Biosens. Bioelectron.25 (2010) 2351–2355
(29) Int. J. Electrochem. Sci. 8 (2013) 1938-1948
(30) J. Electroanal. Chem. 661 (2011) 336–342
(31) Anal. Methods 4 (2012) 259-264
(32) J. Mol. Liq.178 (2013) 63–69
(33) J. Solid State Electrochem. 17 (2013) 661–665
(34) Sens. Actuators, B 171–172 (2012) 380–386
(35) Biosens. Bioelectron.39 (2013) 124–132
(36) Microchim. Acta 165 (2009)373–379
(37) J. Nanosci. Nanotechnol. 12 (2012) 1903-1909
(38) Biosens. Bioelectron.42 (2013) 434–438
(39) Electroanalysis 23 (2011) 1726 – 1735
(40) Electrochim. Acta 90 (2013) 317– 325
(41) Sens. Actuators, B 176 (2013) 543– 551
(42) J. Electroanal. Chem.698 (2013) 45–51
(43) Biosens. Bioelectron.35 (2012) 75– 81
(44) Electrochim. Acta 56 (2011) 9113– 9120
(45) Bioelectrochemistry 86 (2012) 22–29
(46) Sens. Actuators, B 166–167 (2012) 508–518
(47) J. Solid State Electrochem. 15 (2011) 845–853
(48) Electrochim. Acta 55 (2010) 4711–4716
(49) J. Electroanal. Chem.636 (2009) 40–46
(50) J. Electrochem. Soc. 159 (2012) H765-H771
(51) Biosens. Bioelectron. 39 (2013) 124–132
(52) J. Mol. Liq. 173 (2012) 137–143
(53) J. Serb. Chem. Soc. 74 (2009) 1443–1453
(54) Int. J. Electrochem. (2011) Article ID 185864, 7 pages
(55) J. Anal. Met. Chem. (2012) Article ID 305872, 8 pages
(56) Int. J. Electrochem. Sci. 7 (2012) 2430-2439

Table S8. Electroanalytical figures of merit for ATC code N05 drugs. M: method; EM: electrode material; LOD: limit of detection; LWR: linear working range; P: peak potential; SE: supporting electrolyte. References are presented as footnotes below the table.

Drug (Analyte)	***Significant Details and Figures of Merit***	***Ref.***
Buspirone	M: DPV; EM: Nanosilver-DNA Hybrid modified glassy carbon electrode ; LWR: 2 to 70 nM; LOD:1.1 nM	(1)
Chlorpromazine	M: DPV; EM: multiwalled carbon nanotube-polyethyleneimine composite modified glassy carbon electrode ; LWR: 0.019 to 9.2 µM; LOD:10 nM ; SE: pH 7.0 phosphate buffer; P:0.685 V; applied to determination of chlorpromazine in tablets	(2)
Chlorpromazine	M: DPV; EM:Cobalt nanoparticles modified CPE; LWR: 0.002–1 µM; LOD:0.6 nM ; SE: pH 4.0 phosphate buffer; P:0.78 V; applied to determination of chlorpromazine in urine and serum	(3)
Clozapine	M: LSV; EM: MWCNTs/New Coccine doped PPY modified GCE ; LWR: 0.01–0.4 and 0.4-5.0 µM; LOD: 3 nM; SE: pH 5.44 B. R. buffer ; P:0.52 V; applied to determination of clozapine in pharmaceutical formulations and plasma	(4)

Clozapine	M: AdSDPV; EM: TiO_2 nanoparticles modified CPE; LWR: 0.5–45 µM; LOD:0.008 µM ; SE: pH 7.2 ; P: 1.34 V; applied to determination of clozapine in pharmaceuticals products; simultaneous determination of clozapine with thiordazine	(5)
Risperidone	M: DPV; EM: MWCNT modified CPE; LWR: 0.04–10 µM; LOD:0.012 µM ; SE: pH 8.0 B. R. buffer; P: -0.8 V; applied to determination of risperidone in pharmaceutical formulations and human serum	(6)
Risperidone	M: AdSDPV; EM: MWCNT- *n*-octylpyridinum hexafluorophosphate ionic liquid composite modified GCE; LWR: 10–200 nM; LOD:6.54 nM ; SE: pH 4.0 B. R. buffer; P: 0.23 V; simultaneous determination of risperidone and clozapine ; applied to determination of risperidone in pharmaceutical formulations and human serum	(7)
Thioridazine	M: DPV; EM: MWCNT with immobilized cobalt nanoparticles modified GCE; LWR: 0.5–100 µM; LOD:50 nM ; S. E.: pH 7.0 ; P: 0.52 V; analysis of thioridazine in pharmaceutical formulations and blood serum	(8)
Thioridazine	M: DPV; EM: nitrogen-doped carbon nanotubes/gold composites modified GCE; LWR:12–850 µM; LOD: 1.3 µM ; SE: pH 6.0 phosphate buffer; P: 0.47 V	(9)
Thioridazine	M: DPV; EM: CPE modified with ZnS nanoparticles; LWR: 0.1 to 36 µM; LOD: 65 nM; SE: pH 7.0 phosphate buffer; P: 0.62 V; simultaneous determination of thioridazine and olanzapine in urine	(10)
Thioridazine	M: AdSDPV; EM: Nanodiamond_graphite decorated with Ag nanoparticles modified pyrolytic graphite electrode; LWR: 0.08 to 100 µM; LOD: 80 nM; SE: pH 7.0 phosphate buffer; P: 0.65 V; analysis of thioridazine in pharmaceutical formulations and blood serum	(11)

References

(1) Sens. Lett., Volume 10 (2012) 814-820
(2) Int. J. Electrochem. Sci., 7 (2012) 11414-11425
(3) J. Electroanal. Chem. 683 (2012) 31–36
(4) Bioelectrochemistry 90 (2013) 36–43
(5) Electrochim. Acta 87 (2013) 816– 823
(6) Anal. Methods, 4 (2012) 1415-1420
(7) J. Chin. Chem. Soc. 60 (2013) 63-72
(8) Biosens. Bioelectron. 24 (2009) 3235–3241
(9) J. Solid State Electrochem. 16 (2012) 2691–2698
(10) Electroanalysis 24 (2012) 2193-2202
(11) Electroanalysis 25 (2013) 417-425

Table S9. Electroanalytical figures of merit for ATC code N06 drugs. M: method; EM: electrode material; LOD: limit of detection; LWR: linear working range; P: peak potential; SE: supporting electrolyte. References for Table S8 are present as footnotes below the table.

Drug (Analyte)	*Significant Details and Figures of Merit*	*Ref.*
Caffeine	M: SWV; EM: Multiwall carbon nanotube modified glassy carbon electrode ; LWR:10 µM to 500 µM; LOD:3.52 nM; SE: pH 7.2 ; P: 1.103 V; simultaneous determination of ascorbic acid and caffeine ; applied to analysis of AA and CAF in tea leaves, coffee, cold drink, pharmaceutical formulations and urine	(1)
Caffeine	M: DPV; EM: Pt/carbon nanotubes composite modified glassy carbon electrode ; LWR: 1 µM to 100 µM; LOD:0.57 µM ; SE: 0.01 M sulfuric acid	(2)
Caffeine	M: DPV; EM: single-walled carbon nanotubes on carbon-ceramic electrode ; LWR: 0.25 µM to 100µM; LOD:0.12 µM ; SE: pH 1.7 sulfuric acid; P:1.38 V; applied to determination of caffeine in mineral water	(3)
Caffeine	M: AdSDPV; EM:GCE modified Nafion and MWCNT; LWR: 2.945–377 µM and 377–2356 µM; LOD:0.513 µM ; SE: pH 4.1 B. R. buffer; P:1.34 V; applied to	(4)

	determination of caffeine in cola, energy drinks and green tea	
Caffeine	M: AdSDPV; EM:GCE modified Nafion and MWCNT; LWR: 0.6–400 µM; LOD:0.23 µM; SE: pH 2.0 sulfuric acid ; P:1.33 V; applied to determination of caffeine in tablets and cola	(5)
Caffeine	M: SWV; EM: EPPGE; LWR: 0.02–100 µM; LOD:0.008 µM ; SE: pH 7.2 ; P: 1.34 V; applied to determination of CF in pharmaceuticals products, human urine and coffee and tea beverages	(6)
Caffeine	M: DPV; EM: BDDE; LWR: 0.5–83 µM; LOD:0.035 µM ; SE: pH 4.5 acetate buffer; P: 1.32 V; simultaneous determination of paracetamol and caffeine ; applied to determination of CF in pharmaceuticals products	(7)
Clomipramine	M: DPV; EM:poly-aminobenzene sulfonic acid/Pt nanoclusters modified GCE; LWR: 2.5–105 µM; LOD:1 nM; S. E.: pH 8.1 ; P: 0.72 V; analysis of clomipramine in pharmaceutical formulations	(8)
Trazodone	M: DPV; EM: MWCNT modified GCE ; LWR:0.2–10 µM; LOD: 24 nM ; SE: pH 7.0 phosphate buffer; P: 0.68 V; Ascorbic acid interfered with the analysis; analysis of Trazodone in pharmaceutical formulations	(9)
Imipramine	M: AdSDPV; EM: Amberlite XAD-2 and titanium dioxide nanoparticles modified glassy carbon paste electrode; LWR: 1.30 to 6230 nM; LOD: 0.393 nM; SE: pH6 phosphate buffer; P: 0.82 V; analysis of imipramine in pharmaceutical formulations, urine and blood serum	(10)
Trimipramine	M: AdSDPV; EM: Amberlite XAD-2 and titanium dioxide nanoparticles modified glassy carbon paste electrode; LWR: 1.16 to 6870 nM; LOD: 0.351 nM; SE: pH6 phosphate buffer; P: 0.79 V; analysis of trimipramine in pharmaceutical formulations, urine and blood serum	(10)
Desipramine	M: AdSDPV; EM: Amberlite XAD-2 and titanium dioxide nanoparticles modified glassy carbon paste electrode; LWR: 1.43 to 5680 nM; LOD: 0.435 nM; SE: pH6 phosphate buffer; P: 0.85 V; analysis of desipramine in pharmaceutical formulations, urine and blood serum	(10)
Venlafaxine	M:AdSDPV; EM: Nafion-carbon nanotube-modified glassy carbon electrode; LWR: 0.038-62.2 µM; LOD: 12.4 nM; SE: pH 7.0 B. R. buffer; analysis of venlafaxine in pharmaceutical formulations, urine and blood serum	(11)
Desvenlafaxine	M:AdSDPV; EM: Nafion-carbon nanotube-modified glassy carbon electrode; LWR: 0.053-35.8 µM; LOD: 21.1 nM; SE: pH 5.0 acetate buffer; analysis of desvenlafaxine in pharmaceutical formulations, urine and serum	(11)

References

(1) Electrochim. Acta 93 (2013) 248– 253
(2) Chemia Analityczna 54 (2009) 607-617
(3) Chin. J. Catal., 33 (2012) 1783–1790
(4) Int. J. Electrochem. Sci. 6 (2011) 997-1006
(5) J. Electroanal. Chem. 639 (2010) 77–82
(6) J. Electroanal. Chem. 655 (2011) 97–102
(7) Talanta 78 (2009) 748–752
(8) Anal. Lett. 40 (2007) 3392–3404
(9) Talanta 79 (2009) 361–368
(10) Analyst 138 (2013) 1395-1404
(11) Electrochim. Acta 56 (2011) 4188–4196

Table S10. Electroanalytical figures of merit for ATC code N07 drugs. M: method; EM: electrode material; LOD: limit of detection; LWR: linear working range; P: peak potential; SE: supporting electrolyte. References for Table S10 are present as footnotes below the table.

Drug (Analyte)	***Significant Details and Figures of Merit***	***Ref.***
Cinnarizine	M: DPV; EM:MWCNT /GCE; LWR:0.09 to 6.0 µM; LOD: 2.58 nM; SE: pH 2.5 B. R. buffer; P:1.2V; applied to analysis of cinnarizine in pharmaceutical formulations and urine .	(1)
Dextro-methorphan	M: amperometry; EM: carbon nanotube–carbon microparticle–ionic liquid composite; LWR:0.25-3.3 mM ; LOD:8.81 µM ; SE: pH 7.4 phosphate buffer; applied to the determination of dextromethorphan in pharmaceutical formulations	(2)
Naltrexone	M: LSV; EM:Glassy Carbon Electrode Modified with a Bilayer of Multiwalled Carbon Nanotube and Polypyrrole Doped with Nitrazine Yellow; LWR: 0.04 to 10 µM; LOD:12 nM; SE: pH 6.0 B. R. buffer; P: 0.61V; applied to determination of naltrexone in pharmaceutical formulation and serum	(3)
Naltrexone	M: DPV; EM: glassy carbon electrode modified with Nafion-doped carbon nanoparticles ; LWR: 1 to 10 µM and 10 to 100 µM; LOD:0.1 µM ; SE: pH 3.0 ; P: 0.7 V; applied to determination of naltrexone in formulations and plasma	(4)
Nicotine	M: amperometry; EM: MWCNT–alumina-coated silica nanocomposite modified glassy carbon electrode ; LWR: 2.4–50 µM; LOD: 1.42 µM ; SE: pH 8.0 phosphate buffer; applied analysis of nicotine in urine and blood serum	(5)
Nicotine	M: amperometry; EM: MWCNT/BPPGE ; LOD:1.5 µM ; SE: pH 8.0 B. R. buffer; P: 0.7 V	(6)

References

(1) Colloids Surf., B: Biointerfaces 72 (2009) 259–265
(2) J. Solid State Electrochem. 14 (2010) 1515–1523
(3) Electroanalysis 23 (2011) 2925 – 2934
(4) J. Electroanal. Chem. 638 (2010) 212–217
(5) Electrochem. Commun. 11 (2009) 733–735
(6) Sens. Actuators, B 144 (2010) 153–158
